# The safety, reactogenicity, and immunogenicity of the self-amplifying mRNA COVID-19 vaccine GRT-R910 as a booster in healthy adults

**DOI:** 10.1016/j.vaccine.2026.128358

**Published:** 2026-02-25

**Authors:** Jennifer A. Whitaker, Paulina A. Rebolledo, Getahun Abate, Tara M. Babu, Nadine G. Rouphael, Anna Wald, Hana M. El Sahly, Karin Jooss, Meghan G. Hart, Mat Makowski, Jinjian Mu, Andrea Carmack, Janet I. Archer, Paul C. Roberts, Mamodikoe Makhene, Christine M. Posavad, M. Juliana McElrath, Stephen C. De Rosa, Rhea Coler, David Montefiori, Amanda Eaton, Mehul S. Suthar, Robert L. Atmar, Daniel F. Hoft, DMID 20-0034 Study Team

**Affiliations:** a Departments of Molecular Virology and Microbiology and Medicine, Baylor College of Medicine, Houston, TX, USA; b Hope Clinic, Division of Infectious Diseases, Emory University School of Medicine, Atlanta, GA, USA; c Hubert Department of Global Health, Rollins School of Public Health, Emory University, Atlanta, GA, USA; d Center for Vaccine Development, Division of Infectious Diseases, Allergy and Immunology, Departments of Internal Medicine, Saint Louis University, Saint Louis, MO, USA; e Division of Allergy and Infectious Diseases, Department of Medicine, University of Washington, Seattle, WA, USA; f Department of Epidemiology, University of Washington, Seattle, WA, USA; g Department of Laboratory Medicine and Pathology, University of Washington, Seattle, WA, USA; h Vaccine and Infectious Diseases Division, Fred Hutchinson Cancer Center, Seattle, WA, USA; i Gritstone bio, Inc. Emeryville, CA, USA; j The Emmes Company, LLC, Rockville, MD, USA,; k Division of Microbiology and Infectious Diseases, NIAID, NIH, Rockville, MD, USA,; l Seattle Children’s Research Institute, Center for Global Infectious Disease Research, Seattle, WA, USA; m Department of Surgery, Duke University Medical Center, Durham, NC, USA; n Duke Human Vaccine Institute, Duke University Medical Center, Durham, NC, USA; o Center for Childhood Infections and Vaccines, Children’s Healthcare of Atlanta, Division of Infectious Diseases, Department of Pediatrics, Emory University School of Medicine, Atlanta, GA 30329, USA; p Department of Medicine, Section of Infectious Diseases, Baylor College of Medicine, Houston, TX, USA; q Department of Molecular Microbiology & Immunology, Saint Louis University, Saint Louis, MO, USA; r FHI 360, Durham, NC, USA

**Keywords:** COVID-19, Vaccine, Self-amplifying, mRNA, GRT-R10

## Abstract

**Background:**

GRT-R910 (Gritstone bio, Inc), a self-amplifying mRNA vaccine expressing SARS CoV-2 (D614G) spike protein and T-cell epitopes, was evaluated as a booster vaccine in a phase 1 study in 2021–2022.

**Methods:**

This open-label, dose escalation study enrolled healthy adults ≥112 days after completion of primary COVID-19 vaccination, booster of approved mRNA COVID-19 vaccine, or known SARS-CoV-2 infection. Persons aged 18–60 years received single doses of 3 or 6 μg GRT-R910 (*n* = 10/group). Persons >60 years of age received GRT-R910 at 3, 6, or 10 μg (*n* = 8–10/group). Safety and immunogenicity responses were assessed for 1 year after vaccination.

**Results:**

We enrolled 48 participants. Most participants developed mild-to-moderate systemic reactions and/or injection site tenderness. Eight of 48 (17%) had severe systemic reactions. Pseudovirus neutralizing antibody geometric mean fold rise (GMFR) responses against SARS-CoV-2 (D614G) at Day 29 and Day 181, respectively, among those ≤60 years were 5.2 (95% CI 2.1, 13.3) and 6.1 (2.8, 13.2) after 3 μg, and 3.6 (1.3, 10.4) and 2.4 (0.2, 33.0) after 6 μg. The GMFR responses among those aged >60 years were 8.1 (2.1, 31.4) and 8.2 (1.2, 57.5) after 3 μg, 2.7 (1.1, 6.7) and 1.8 (0.5, 6.7) after 6 μg, 3.3 (1.5, 7.4) and 3.3 (1.3, 8.0) after 10 μg. The 6 μg dose group in those ≤60 years, 6 μg and 10 μg dose groups in those aged >60 years had higher baseline geometric mean titers (GMTs), which, in turn may have lowered the GMFR for those groups.

GMFR persistence until Day 181 in most groups indicated these boosts were associated with durable increases in GMFR. Neutralizing antibody titers assessed via focus reduction neutralization test against SARS-CoV-2 D614G largely mirrored the PsVNA findings.

**Conclusions:**

GRT-R910 was safe but reactogenic when administered to previously vaccinated or infected adults and boosted anti-SARS-CoV-2 neutralizing antibody responses in most participants with responses that appeared durable for up to 6 months.

**Clinical trial registration:**

https://clinicaltrials.gov/study/NCT04776317.

ClinicalTrials.gov ID NCT04776317.

## 1. Introduction

The coronavirus disease 2019 (COVID-19) pandemic, caused by Severe Acute Respiratory Syndrome Coronavirus 2 (SARS-CoV-2), resulted in over 777 million cases and 7 million deaths by January 2025 [1]. The authorized and approved COVID-19 vaccines were successful in preventing severe COVID-19 and saving millions of lives [2]. However, there is a need for COVID-19 vaccines that provide long-term protection against infection and disease across emerging variants, especially in vulnerable populations [3]. Repeated waves of infection caused by SARS-CoV-2 variants have prompted booster vaccination campaigns [3]. Currently approved and authorized COVID-19 vaccines target the SARS-CoV-2 Spike (S) protein, which is prone to mutate, leading to immune escape as new variants emerge. While neutralizing antibodies and binding antibodies against S protein from the ancestral strain have correlated with protection against COVID-19 in vaccine efficacy trials [4,5], T cell responses also have an important role in reducing disease severity and controlling infection [6–11]. While viral surface antigens containing B cell epitopes tend to mutate at rates higher than internal proteins, more conserved viral genes containing T cell epitopes (TCEs) that are relevant against all viral strains can be targeted [12]. A vaccine that includes highly conserved SARS-CoV-2-specific TCEs may enhance the breadth and durability of protection against COVID-19 [12].

Self-amplifying mRNA (SAM) vaccines tend to elicit higher and more durable transgene expression in comparison to non-amplifying mRNA vaccines [13], resulting in robust and longer-lasting neutralizing antibody (nAb) responses in animal models, while also being dose-sparing [14–17]. Clinical trials have evaluated the safety and immunogenicity of SAM COVID-19 vaccines in persons who previously received authorized COVID-19 vaccines [15,18–23]. However, some studies of SAM COVID-19 vaccines have reported dose-limiting reactogenicity [18,19,23]. GRT-R910 is an investigational vaccine that utilizes a synthetic Venezuelan Equine Encephalitis Virus (VEEV) based SAM vector formulated into lipid nanoparticles. GRT-R910 encodes for full-length, stabilized prefusion S derived from the Wuhan Hu-1 strain as well as a cassette of highly conserved non-spike TCE sequences derived from an HLA-based peptide binding algorithm [24] and previously validated epitopes [25,26]. The epitopes were selected to provide broad HLA-coverage and include conserved regions across coronaviruses [21]. Here, we report the results of the safety, reactogenicity, and immunogenicity of GRT-R910 administered as a booster vaccine to adults aged 18–60 years and > 60 years who previously completed an approved mRNA COVID-19 vaccine series. Some of the later enrolled groups in the trial also received an approved mRNA COVID-19 booster prior to GRT-R910.

## 2. Materials and methods

### 2.1. Study design

This was a multi-center, open-label, dose-escalation, non-randomized study in healthy adults of the safety and reactogenicity (primary endpoint) and immunogenicity (secondary endpoint) of several investigational COVID-19 vaccines produced by Gritstone (NCT04776317) (Supplementary Table 1). Single-dose boosters of GRT-R910 were evaluated among persons aged 18–60 years (Groups 5 and 6) and over 60 years (Groups 9–11) who had previously completed an approved mRNA COVID-19 vaccine series (Table 1). In this report, we describe the findings of Groups 5, 6, 9, 10, and 11 who received GRT-R910. Healthy, non-pregnant persons aged ≥18 years of age who had completed a primary approved mRNA COVID-19 vaccine series, with or without receipt of a prior COVID-19 mRNA booster, at least 112 days before vaccination were enrolled in these groups. Groups 5 and 6 enrolled participants aged 18–60 years to receive GRT-R910 at 3 μg and 6 μg, respectively, and Groups 9, 10, and 11 enrolled persons >60 years of age to receive 3 μg, 6 μg, and 10 μg, respectively, of GRT-R910 (Table 1). No participants in groups 5, 6, or 9 received additional approved COVID-19 booster vaccines beyond the primary COVID-19 vaccine series. All participants in groups 10 and 11 received an additional approved mRNA COVID-19 vaccine booster at least 112 days before enrollment. Participants and site staff were unblinded to participants’ group assignments. Initially, the protocol excluded participants who had a history of prior SARS-CoV-2 infection, but due to the increasing prevalence of infection observed during the study, the protocol was amended to allow enrollment of previously infected persons into Groups 10 and 11 as long as the infection occurred ≥112 days prior to study vaccination. The study used a sentinel approach for enrollment that consisted of observation of the first three participants in each group for 72 h before enrolling the remaining 7 participants in the group. Prior to moving to the next dosage group, the aggregate safety data through Day 8 post study vaccination for at least seven participants/group, including the Day 8 post first study vaccination safety labs, reactogenicity, and any adverse events (AEs), were reviewed by the Study Steering Committee.

### 2.2. Ethical considerations

This multi-center study utilized a single IRB of record, Vanderbilt University IRB. Participants provided written informed consent prior to any study procedure. An independent data and safety monitoring board oversaw the study.

### 2.3. Study vaccine

The GRT-R910 vector encodes the VEEV proteins as well as the 5′ and 3′ RNA sequences required for self-amplification but encodes no structural proteins, so no infectious viral particles are formed. The first open reading frame (Orf) in the cassette encodes validated or predicted CD8+ TCEs from SARS-CoV-2 antigens, Orf3a, nucleocapsid (N), and membrane (M) [24–26]. The second cassette expresses the prefusion-stabilized Spike protein from the Wuhan-Hu1 strain and includes the D614G mutation, 2 proline substitutions at amino acid positions 986 and 987 and mutation at the furin cleavage site. GRT-R910 is a dispersion of nucleic acid encapsulated in lipid nanoparticles (LNPs) composed of an ionizable amino lipid, a phosphatidylcholine, cholesterol, and a polyethylene glycol-based coat lipid. GRT-R910 was stored at −60 degrees Celsius or colder, and it was thawed to ambient temperature and mixed gently prior to administration. The vaccine was administered as a single 0.25 mL (for the 3 and 6 μg doses) or 0.5 mL (for the 10 μg dose) intramuscular injection in the deltoid muscle.

### 2.4. Study procedures

Following vaccination (Day 1), participants recorded pre-specified solicited injection site and systemic reactions for 7 days. The study team assessed unsolicited AEs for 28 days after vaccination. AEs and serious AEs (SAEs) were graded utilizing the Food and Drug Administration (FDA)’s “Toxicity Grading Scale for Healthy Adult and Adolescent Volunteers Enrolled in Preventative Vaccine Clinical Trials [27],” as follows: mild (1; no interference with normal activities), moderate (2; some interference with normal activities), or severe (3; prevented normal activities). New-onset chronic medical conditions (NOCMCs), medically attended adverse events (MAAEs), including adverse events of special interest (AESI), and SAEs were recorded from the time of study vaccination through approximately 12 months after vaccination. AESIs included serologically or virologically confirmed SARS-CoV-2 infection, severe COVID-19 disease, myocarditis/pericarditis, Guillain-Barré syndrome, thrombocytopenia with thrombosis syndrome/vaccine-induced thrombotic thrombocytopenia. Clinical laboratory safety evaluations (white blood cell count, hemoglobin, and platelet count, alanine aminotransferase, aspartate aminotransferase, alkaline phosphatase, total bilirubin, creatine kinase, serum creatinine, and prothrombin time/partial thromboplastin time) were performed at screening and 7 days after the study vaccinations. COVID-19 infection was assessed during the study by participant self-report and measurement of N-protein antibody binding assay at Days 1, 85, and 181.

### 2.5. Immunogenicity laboratory assays.

Laboratory methods are described in the Supplemental materials. ELISAs measured IgG binding antibody to SARS-CoV-2 S-2P and receptor binding domain (RBD) antigens and were performed using a qualified high throughput assay as previously described [28]. Serum neutralizing antibodies were measured in a validated pseudovirus-based assay as a function of reductions in luciferase reporter gene expression after a single round of infection with either SARS-CoV-2.D614G, SARS-CoV-2.BA1.1.529, or SARS-CoV-2.BA.4/BA.5 spike-pseudotyped virus in 293 T/ACE2 cells (293 T cell line stably overexpressing the human ACE2 cell surface receptor protein as previously described) [29]. Serum neutralization assays using live SARS-CoV-2.D614G, SARS-CoV-2. BA1.1.529, and SARS-CoV-2.BA.4/BA.5 were conducted via focus reduction neutralization test (FRNT) as previously described [30–34].

Ex vivo ELISpot assays for the detection of IFN-γ-producing T cells were performed as described in the Supplemental materials. Data were presented as spot forming units (SFU) per million cells. Ex vivo ELISpot limit of detection (LOD) was determined at 30 SFU/10^6^ PBMCs. T cell epitope (TCE) ELISpot responses were calculated by the sum of responses to overlapping peptide (OLP) pools covering N, M and ORF3a regions of the TCE cassette. Responses below LOD were converted to half the value of LOD. If vehicle responses were above LOD, positive responses were determined at ≥2-fold increase compared to vehicle control. A participant was considered a responder if the baseline sample mean was <LOD and the post-vaccination test sample mean was ≥2-fold of LOD or the baseline sample mean was ≥ LOD and the post-vaccination test sample mean was ≥2-fold of baseline mean. The definition of a responder on ELISpot was for each individual peptide. Because the “Spike” outcome was the sum of 4 peptide values used, if any of the 4 peptide responses met the definition of a responder then the participant was considered as a “responder” for the overall “Spike” response. A similar rule was used for the other summed outcome of overlapping peptides covering N, M, and ORF3a regions of the TCE cassette. If any of the peptide responses met the definition of a “responder,” then the participant was considered a responder for the TCE peptides. Detection of secreted IL-2, TNF-α, IL-4, IL-10, and IL-13 in ex vivo ELISpot supernatants was performed in a subset of participants using an MSD U-PLEX Biomarker assay.

Flow cytometry was used to examine SARS-CoV-2-specific CD4+ and CD8+ T-cell responses using a validated intracellular cytokine staining (ICS) assay as described in the Supplemental materials [35,36].

### 2.6. Outcome measures and statistical analyses

The primary safety endpoints were the occurrence of solicited injection site and systemic reactogenicity signs and symptoms, occurrence of unsolicited AEs, change of clinical safety laboratory parameters, and occurrence of SAES, AESIs, including potential immune-mediated medical conditions (PIMMCs), medically attended adverse events (MAAEs), and NOCMCs. The secondary humoral immunogenicity endpoints were the response rate and magnitude of SARS-CoV-2 specific antibody binding and neutralization titers assessed via assays measuring total Spike-specific IgG (ELISA-based) and function (neutralization, receptor binding domain (RBD)-binding) on Days 1, 15, 29, 85, 181, and 366. The secondary T cell immunogenicity endpoints were the response rate, magnitude, and functional profiling of SARS-CoV-2 specific T cells stimulated with overlapping peptides (OLPs), consisting of 15mers covering Spike and TCE regions, pre- and post-vaccination using interferon-γ (IFN-γ) enzyme-linked immunosorbent spot (ELISpot) analysis of all participants at Days 1, 15, 29, 85, 181, and 366, and using intracellular cytokine staining (ICS) assays at Days 1 and 15. A subset of participant samples were assessed for Th1/Th2 cytokine balance of T cell response at 4-weeks post-vaccination by measuring interleukin-2 (IL-2), tumor necrosis factor-α (TNF-α), IL-4, IL-10, and IL-13 using ELISPOT analysis of supernatants.

The safety analyses were conducted on the safety population defined as all participants who received study vaccine. The modified intention-to-treat (mITT) population included all participants who received study vaccination and contributed both pre- and one post dose blood sample(s) for immunogenicity testing for which valid results were reported. The per-protocol population (PP) included all participants in the mITT population with the following exclusions: data from all visits for which participants were found to be ineligible at baseline, data from all visits after protocol deviations were considered to affect the science, data for all visits after receipt of an out-of-study COVID-19 vaccine, data from all visits after post-baseline SARS-CoV-2 infection was determined by N-protein antibody testing during the study (Days 1, 85 and 181) or by participant self-report, and data from any visit that occurs substantially out of window. All analyses of the immunogenicity data were completed for the mITT population and the per-protocol populations.

Antibody titers (binding and nAb) are reported by group and time point using GMT and GMFR, including 95% confidence intervals based on the t-distribution. The GMFR is defined as the geometric mean of the ratio of the result at a time point divided by the result at Day 1. The geometric mean ratio (GMR), which is the geometric mean of the ratio defined as the titer of a variant of concern divided by the comparable result against D614G, is reported for BA.1 and BA.4/5 neutralization titers. The BA.1 and BA.4/5 strains were selected because they were the dominant circulating strains at the time of laboratory testing. The seropositivity rate is the percentage of participants with results above the lower limit of detection (LLOD). Undetectable titers were imputed as one-half of the lower limit of quantitation (LLOQ) and the number and proportion of participants with undetectable titers at each time point is reported. ICS endpoints are presented as the percentage of cells expressing various cytokines or combinations of cytokines, including 95% confidence intervals by group, peptide pool/stimulant, and time point. For the ICS assay, results below 0.001 were imputed as 0.001. ELISpot results were summarized by cytokine, group, peptide pool/stimulant, and time point using summary statistics and 95% confidence intervals.

## 3. Results

### 3.1. Study population

**Fig. 1.**
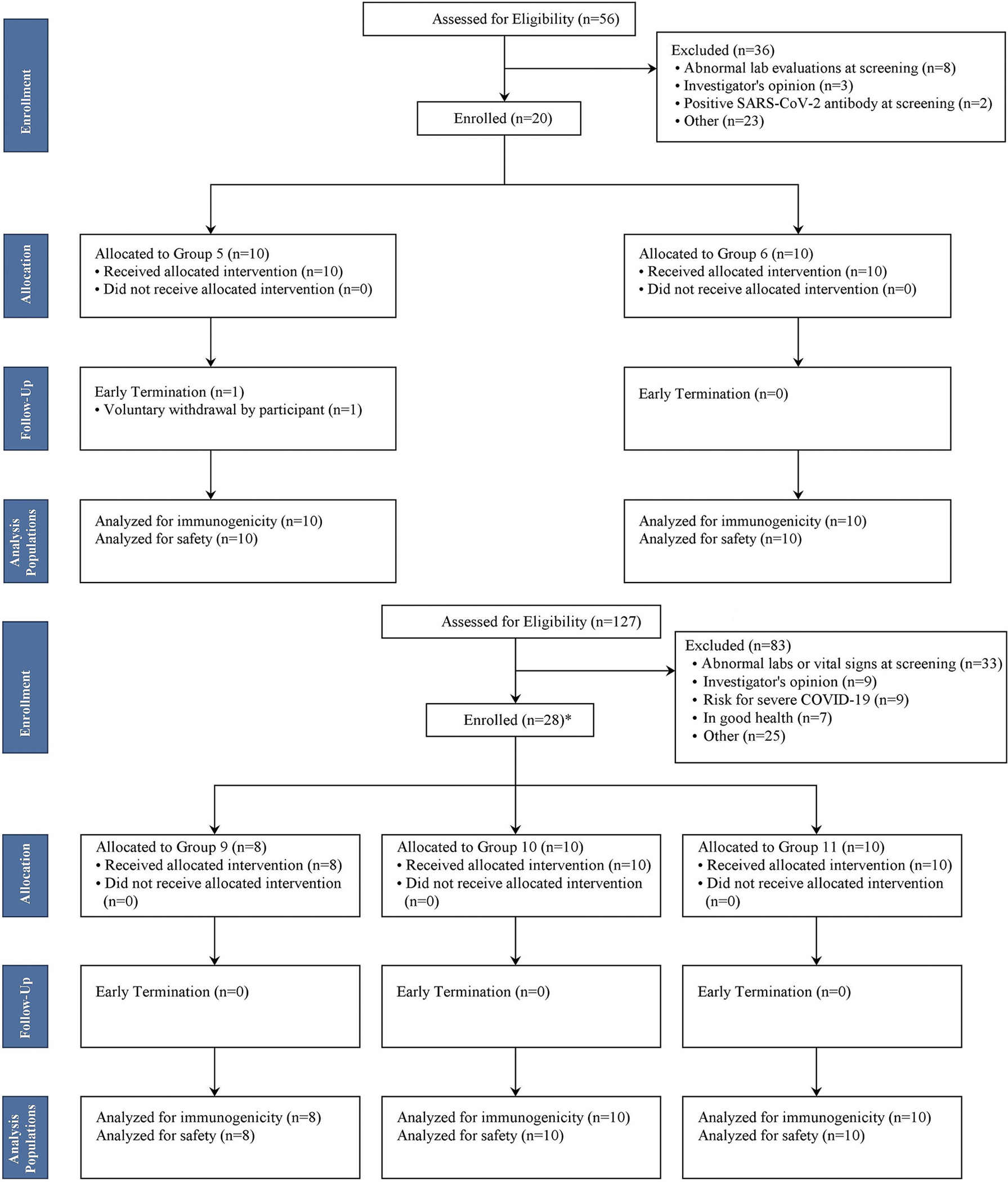
(a) Disposition of study participants 18–60 years of age. (b) Disposition of study participants >60 years of age.

Enrollment occurred between September 2021 and August 2022. Of 56 participants screened in Groups 5 and 6, 20 participants were enrolled and received the allocated vaccination. Overall, 19 (95%) of 20 enrolled participants in Groups 5 and 6 completed the study (Fig. 1), and one participant from group 5 voluntarily withdrew. Of 127 persons over 60 years of age screened for participation, 8, 10, and 10 were enrolled into groups 9, 10, and 11, respectively; all 28 enrolled participants in this age group completed the study (Fig. 1). Table 1 outlines the participant characteristics by vaccine group at baseline. Two participants (20%) each in Groups 10 and 11 had a history of prior SARS-CoV-2 infection concordant with positive N-protein antibodies at baseline. No other participants had positive N-protein antibodies at baseline. No participants in groups 5, 6, or 9 received additional approved COVID-19 booster vaccines. All participants in groups 10 and 11 received an additional approved mRNA COVID-19 vaccine booster at least 112 days before enrollment.

The numbers of participants included in the safety, mITT, and PP populations are depicted in Table 2. Twenty-seven of the 48 participants developed breakthrough infection during the course of this study as determined by conversion of N-protein antibody from negative at baseline to positive or by self-report. Seventeen reported COVID-19 during the study; none were severe. The numbers of participants with positive N-protein antibody tests at Days 1, 85, and 181 are depicted in Supplementary Table 3. Four participants (two in group 10 and two in group 11) tested positive for N-antibody at baseline. Eighteen developed a positive N-protein antibody after enrollment. Seventeen had positive N-antibodies at Day 181. N-protein antibody testing was not conducted at Day 366.

### 3.2. Vaccine safety and tolerability

**Fig. 2.**
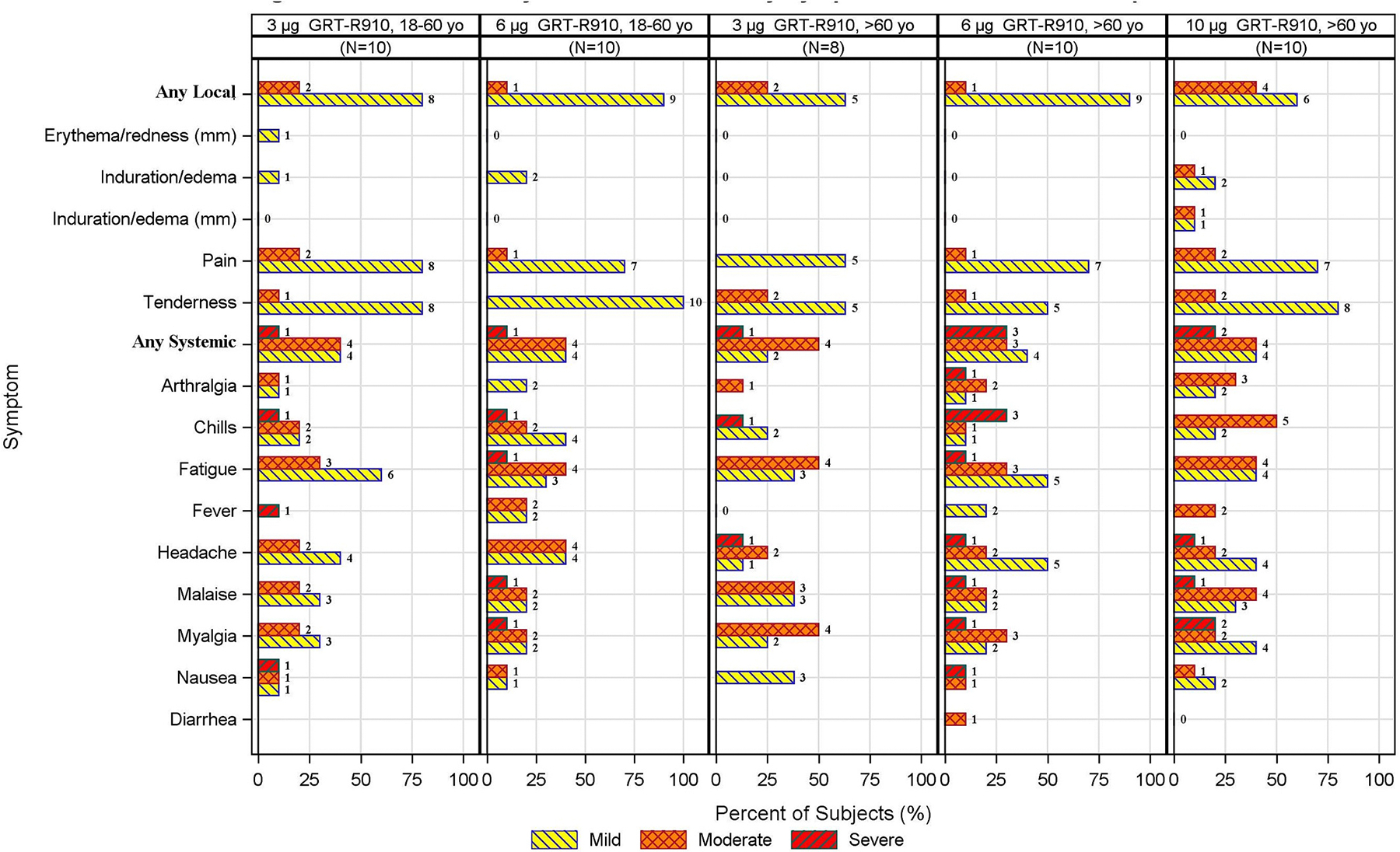
Maximum severity of solicited events by symptom and vaccination group.

Of the 48 participants enrolled, most developed fatigue (85%), headache (69%), myalgia (63%), malaise (60%), chills (56%), injection site pain (83%) and injection site tenderness (88%), including 8 (17%) participants with severe systemic reactogenicity (Fig. 2). In younger adult Group 5 (3 μg dose), four (40%) reported mild, four (40%) moderate, and one (10%) severe solicited systemic symptom which was fever. Most common solicited systemic symptoms were fatigue (90%), headache (60%), myalgia (50%), malaise (50%), chills (50%), and nausea (30%). In younger adult Group 6 (6 μg dose), four (40%) reported mild, four (40%) moderate, and one (10%) severe (chills) systemic symptoms. Four (40%) reported mild/moderate fever. Most common solicited systemic symptoms were fatigue (80%), headache (80%), chills (70%), malaise (50%), and myalgia (50%). All severe symptoms in these groups occurred for a maximum duration of one day.

In older adult Group 9 (3 μg dose), two (25%) reported mild, four (50%) moderate, and one (13%) severe solicited systemic symptoms. None had fever. Most common solicited systemic reactions were fatigue (88%), myalgia (75%), malaise (75%), headache (50%), and nausea (38%). In older adult Group 10 (6 μg dose), four (40%) reported mild, three (30%) moderate, and three (30%) severe solicited systemic symptoms. Two (20%) had mild fever. Most common solicited systemic symptoms were fatigue (90%), headache (80%), myalgia (60%), malaise (50%), chills (50%), and arthralgia (40%). In older adult Group 11 (10 μg dose), four (40%) reported mild, four (40%) moderate, and two (20%) severe solicited systemic symptoms. Two (20%) had moderate fever. Most common solicited systemic symptoms were fatigue (80%), malaise (80%), headache (70%), chills (70%), myalgia (60%), arthralgia (50%), and nausea (30%). All severe graded solicited symptoms in these groups lasted for less than 2 days.

Severe injection site reactions did not occur in any younger or older adult group, and most injection site reactions were mild (Fig. 2).

Within 28 days of vaccination, among Groups 5 and 6, 7 participants (35%) experienced at least one unsolicited AE assessed as related to the study vaccine; two were severe. Both severe unsolicited events were diarrhea, beginning at day 3 after vaccination and lasting 8 days in a participant from Group 5 and the other beginning at day 2 after vaccination and lasting 1 day in a participant from Group 6. The same participant from Group 6 also reported two days of severe headache, fatigue, and myalgias that began 15 days after vaccination. Among Groups 9, 10, and 11, 8 participants (29%) experienced at least one unsolicited AE assessed as related to the study vaccine; two were severe. One of the severe AEs occurred in a participant in Group 9 who had symptoms of a viral-like illness with severe myalgia and arthralgia at Day 21 after vaccination, lasting 3 days and associated with moderate chills and headache. The other severe AE was in a participant in Group 11 who developed severe neutropenia (absolute neutrophil count 500–999 cells/mm^3^) at day 8 after vaccination. Laboratory testing was not repeated; therefore, the duration of the neutropenia was not determined.

There were no graded chemistry abnormalities at Day 8 after study vaccination in the groups in this report. Most hematology laboratory abnormalities after baseline were mild or moderate and related to decreases in hemoglobin (Supplementary Figs. 1 and 2).

There were no SAEs reported during the study. All MAAES were assessed as unrelated to study product in these groups, except for one MAAE that was assessed as related to study vaccine and occurred in a participant from Group 10 who received a 6 μg dose of GRT-R910. This participant experienced palpitations that began 19 days after study vaccination. Evaluations included EKG, cardiac enzymes, Holter monitor, transthoracic echocardiogram and cardiac CT; no signs of myocarditis or pericarditis were found. The participant was assessed by a cardiologist as having self-limited palpitations with transient premature ventricular contractions identified by cardiac workup. All MAAEs were assessed as mild or moderate. One participant in Group 6 was diagnosed with an AESI and PIMMC of urticaria that began 12 days after vaccination with 6 μg GRT-R910, resolved within 7 days, and was assessed to be study vaccine-related. All NOCMCs were assessed as mild or moderate and as not related to the study treatment (Supplementary Table 4).

### 3.3. Humoral immunogenicity

#### 3.3.1. SARS-CoV-2 neutralization responses

**Fig. 3.**
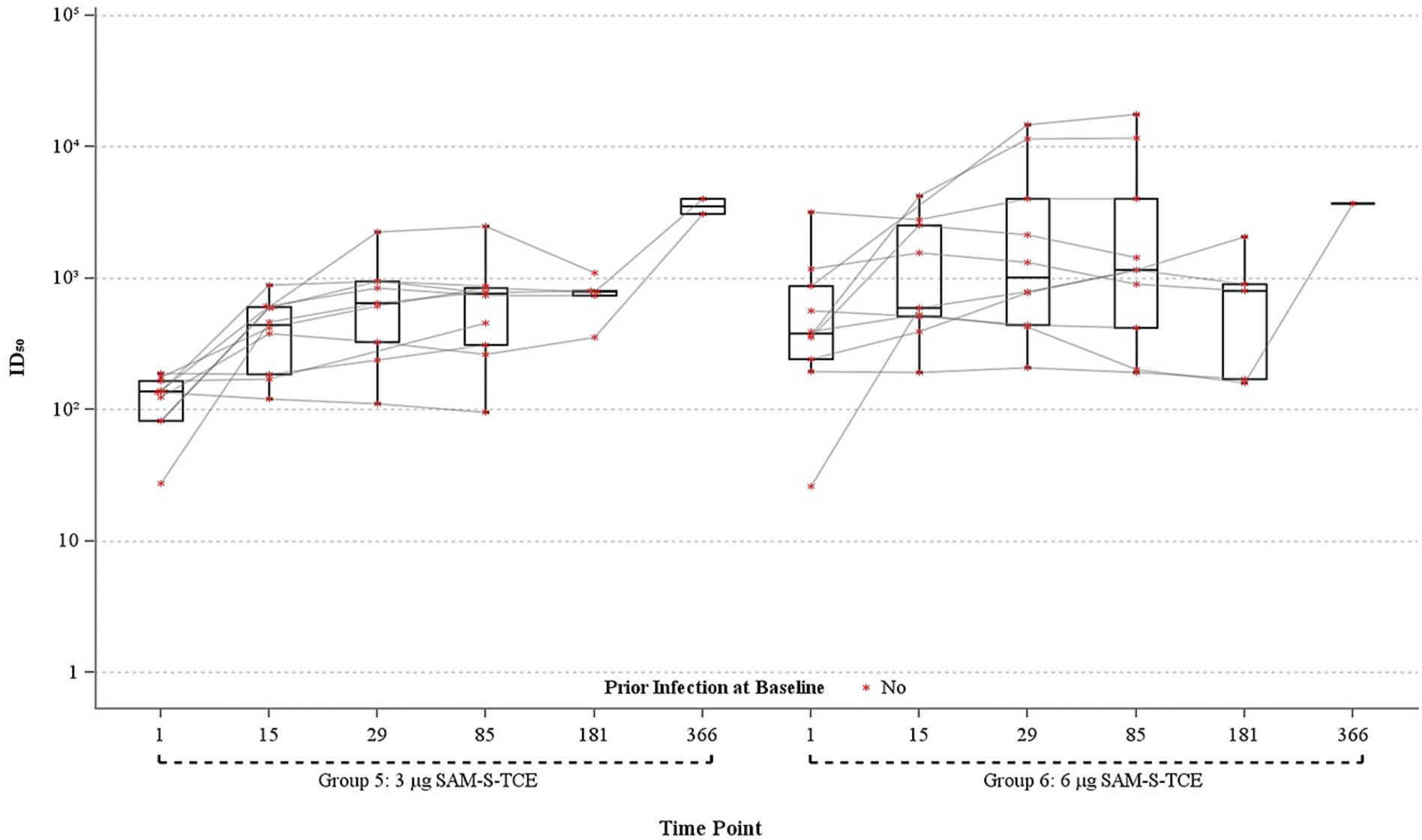
Distribution of pseudovirus neutralizing antibody ID_50_ against SARS-CoV-2 D614G, ages 18–60 Years, per-protocol population.

**Fig. 4.**
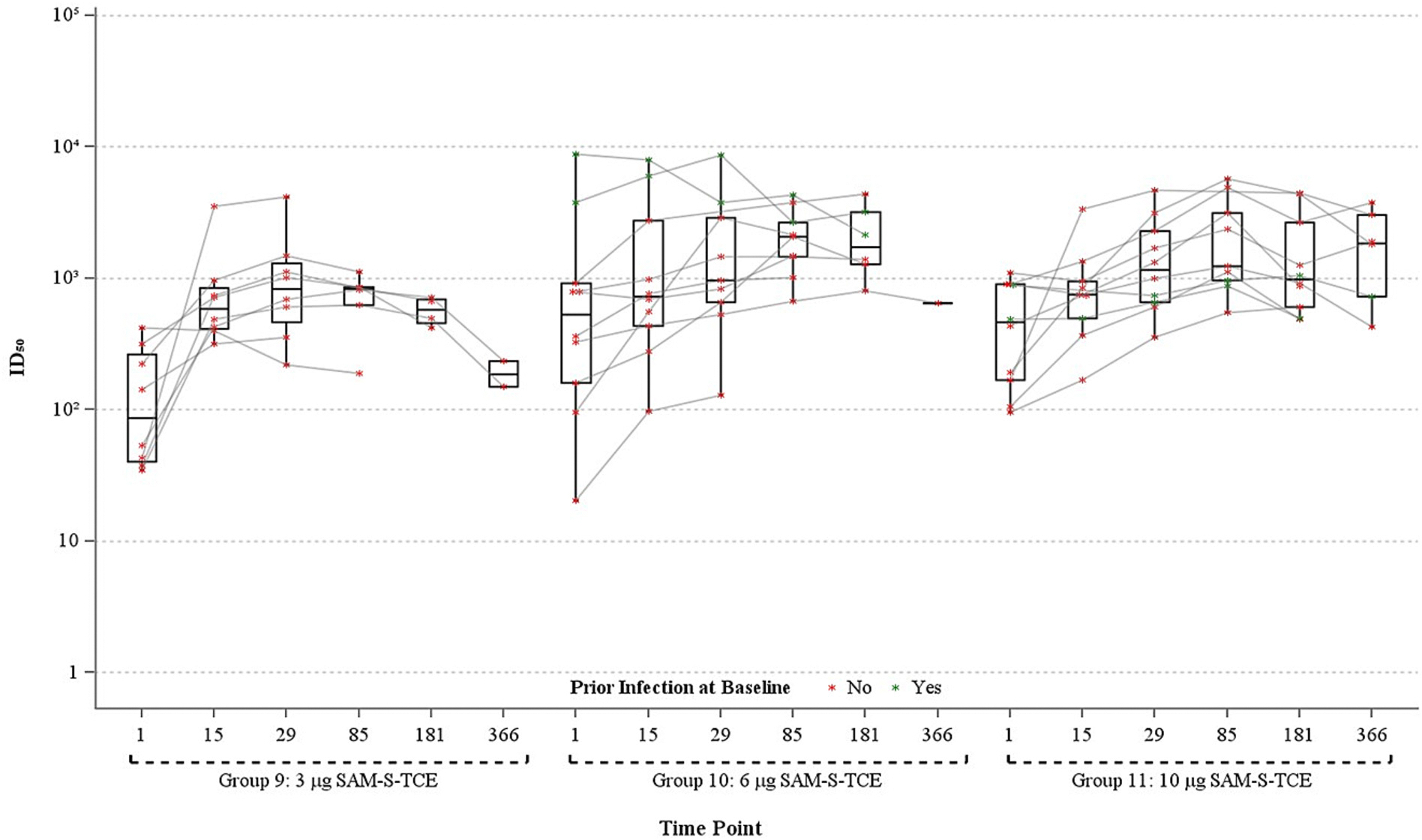
Distribution of pseudovirus neutralizing antibody ID_50_ against SARS-CoV-2 D614G, ages >60 years, per-protocol

Baseline pseudovirus nAb (PsVNA) ID_50_ GMTs against SARS-CoV-2 D614G were higher in Group 6 (405.4, 95% CI: 163.7, 1003.9) than Group 5 (112.8, 95% CI 75.0, 169.8) among participants aged 18–60, and higher in Groups 10 (482. 9, 95% CI: 137.4, 1697.1) and 11 (375.5, 95% CI: 190.3, 740.9) than Group 9 (102.8, 95% CI: 43.7, 242.0) among those >60 years (Fig. 3, Table 3). Elevated titers in Groups 10 and 11 were anticipated due to prior booster vaccination and previous SARS-CoV-2 infection in some participants (Fig. 4, Table 3). All groups demonstrated increases in PsVNA ID_50_ against SARS-CoV-2 at Day 29 with GMTs as follows: 565.2 (95% CI: 285.5, 119.2) [Group 5], 1468.7 (95% CI: 530.3, 4067.8) [Group 6], 829.8 (95% CI: 390.3, 1764.0) [Group 9], 1192.6 (95% CI: 461.7, 3080.4) [Group 10], and 1229.7 (95% CI: 688.4, 2196.6) [Group 11] (Table 3). GMFR against SARS-CoV-2 (D614G) increased for all groups at Day 29 as follows: 5.2 (95% CI: 2.1, 13.3) [Group 5], 3.6 (95% CI: 1.3, 10.4) [Group 6], 8.1 (95% CI: 2.1, 31.4) [Group 9], 2.7 (95% CI: 1.1, 6.7) [Group 10], and 3.3 (95% CI: 1.5, 7.4) [Group 11] (Table 3). The lower GMFRs occurred in groups with higher GMTs at baseline. The PSVNA ID_50_ GMFRs against the D614G variant demonstrated durable responses at Day 85 for all groups and at Day 181 for groups 5, 9, and 11 (Table 3, Figs. 3 and 4). The small sample sizes at Day 366 for each of the groups and the lack of N-protein antibody data at 366 limits the ability to make immunogenicity inferences at Day 366. The marked increases in PsVNA GMTs for groups 5 and 6 between Days 181 and 366 likely indicate an intercurrent SARS-CoV-2 infection (Fig. 4). FRNT results at ID_50_ against SARS-CoV-2 D614G largely mirror the PsVNA findings (Table 4, Supplementary Figs. 3 and 4).

PsVNA and FRNT ID_50_ results for the BA.1 variant demonstrated diminished antibody titer results compared to responses to the vaccine strain. Both PsVNA and FRNT ID_50_ GMFR’s demonstrated durable responses for all groups at Day 85 and at Day 181 for Groups 5, 9, and 11 (Supplementary Tables 5 and 7). PsVNA and FRNT ID_50_ results for the BA.4/5 variant were detected but were low in magnitude (Supplementary Tables 6 and 8).

#### 3.3.2. SARS-CoV-2 binding antibody responses

Minimal increases above baseline were observed in ELISA IgG antibody responses against SARS-CoV-2 S-2P or RBD at post-vaccination time points for Groups 5, 6, 9, 10, and 11 for the mITT or PP populations (Supplementary Tables 9 and 10, Supplementary Figs. 5–8).

#### 3.3.3. SARS-CoV-2T cell responses

T cells expressing IFN-γ after stimulation by spike peptides as measured by ELISpot assays post-vaccination showed that 3/9 (Group 5), 1/9 (Group 6), 2/8 (Group 9), 2/10 (Group 10), and 3/9 (Group 11) participants met the definition of response after vaccination at Day 15 (Supplementary Figs. 9–10). T cells expressing IFN-γ after stimulation to non-spike peptides (spanning membrane, nucleocapsid, and open reading frame 3a) were low or non-detectable in ELISpot assays, with only 1/9 (Group 5) and 1/9 (Group 9) meeting the definition of response at Day 15 after vaccination (Supplementary Figs. 11–12.) By ICS, CD4 T cell responses were observed after stimulation with Spike peptides for most participants with a few showing increases from baseline after vaccination (Supplementary Figs. 13–14). For Groups 5 and 6, changes in the percentages of participants with positive spike-specific CD4 cells expressing IFN-γ and/or IL-2 comparing baseline and Day 15 were, 7/10 (70%) to 8/9 (89%) and 8/10 (80%) to 7/9 (78%), respectively (Supplementary Fig. 13). For Groups 9, 10, and 11, changes from baseline response to Day 15 response were: 5/8 (63%) to 8/8 (100%), 3/8 (38%) to 5/9 (55%), and 7/10 (70%) to 7/9 (78%), respectively (Supplementary Fig. 14). CD4 T cell responses expressing IFN-γ and/or IL-2 after stimulation with TCE epitopes from membrane, nucleocapsid and the open reading frame 3a were only detected in one participant at baseline among groups 5, 6, 9, 10, and 11 with increase from baseline in one participant from group 9 (1/8) and one participant in group 11 (1/9) (Supplementary Figs. 15 and 16). CD8 T cell responses expressing IFN-γ and/or IL-2 after stimulation with TCE epitopes from membrane, nucleocapsid and the open reading frame 3a were only detected in a few participants with only one participant from group 9 experiencing an increase in the response rate from baseline to Day 15 for groups 5, 6, 9, 10, or 11 (Supplementary Figs. 17 and 18). The spike-specific CD4+ T cells did not express or only minimally expressed Th2 cytokines (IL-4, IL-5, and/or IL-13) (Supplementary Figs. 19–20). ELISpot supernatants were used to study a subset of volunteer time points and failed to identify evidence that any Th2-type responses were induced (data not shown). Thus, both IFN-γ ELISPOT and ICS assays demonstrated that the T cell responses were associated with the production of Th1-and not Th2-type of cytokine responses.

## 4. Discussion

We evaluated the safety, reactogenicity, and immunogenicity of the SAM vaccine GRT-R910 as a COVID-19 booster at 3 and 6 μg doses among healthy adults aged 18–60 years and 3, 6, and 10 μg doses among adults aged >60 years. Eight of 48 (17%) participants in the groups in this report experienced severe solicited systemic reactogenicity events in the first 7 days after vaccination. In our study, there did not appear to be a dose response for the observed reactogenicity. Other studies of lipid nanoparticle encapsulated SAM COVID-19 vaccines have reported increasing severity and frequency of solicited local and systemic adverse events with increasing vaccine doses up to 10–15 μg and dose-limiting reactogenicity [18,19,23]. In the first stage of this study when evaluating GRT-908, a SAM COVID-19 product made by Gritstone dosed at 30 μg in persons naïve to COVID-19 infection [Supplementary Table 1], we did note high frequencies of moderate to severe systemic reactogenicity in participants with this dose, which prompted us to redesign stage 2 of the study evaluating GRT-R910 as a booster vaccine starting at 3 μg with escalation to 10 μg. The severe solicited systemic reactogenicity rates we observed were higher than reported in another trial where GRT-R910 was administered as a booster at doses of 10 or 30 μg to persons older than 60 years of age who had completed a primary series with AZD1222 (Oxford Astra-Zeneca ChAdOx1 COVID-19 vaccine) [21]. Another trial evaluating GRT-R914 and R-912 in South Africa among healthy people aged 18–65 years with or without prior COVID-19 infection also reported less reactogenicity (only 6% experienced grade 3 solicited adverse events). Lower rates of severe solicited systemic adverse events also were reported after the mRNA-1273 booster vaccine [37], BNT162b2 [37], and another SAM COVID-19 vaccine (ARCT-154) [15]. It may be that priming with the approved mRNA vaccines leads to greater reactogenicity after a boost with GRT-R910 than priming with adenoviral vectored vaccines, and/or that different populations in different geographic areas may experience differences in reactogenicity due to genetic or environmental differences.

We identified induction of broad nAb responses to SARS-CoV-2 D614G, BA.1, and BA.4/5 variants by GRT-R910. The nAb titers were higher in persons with hybrid immunity. Furthermore, some groups had persistently increased nAb responses against SARS-CoV-2 D614G, BA.1, BA.4/5 lasting at least 6 months, suggesting increased durability of these responses compared to other approved vaccines. The persistence of neutralizing antibody responses at 6 months was consistent with reports of GRT-R910 administered as a booster vaccine to persons aged ≥60 years in the UK who had previously received a primary series with AZD1222 [21]. Durable nAb responses at 6 months were also reported in a preliminary report evaluating GRT-R914 and R-912 (both utilizing the SAM platform with TCE epitopes and encoding the full-length Beta Spike) in South Africa among healthy people aged 18–65 years with or without prior COVID-19 infection [38]. There did not appear to be a dose response to increasing doses of GRT-R910 in the younger or older age cohorts, although, this is difficult to assess given the small sample sizes and lower baseline antibody titers for Groups 5 and 9. Additionally, group 11 had lower baseline antibody titers than group 10, which makes it difficult to assess whether there was a dose response between the 6 μg and 10 μg doses. The durability of nAb responses needs to be confirmed with additional studies. Persons who were vaccinated with mRNA COVID-19 vaccines have been demonstrated to be less likely to develop N-protein antibodies with PCR-confirmed COVID-19 illness than those who were not previously vaccinated [39]. It is possible that some participants became infected with SARS-CoV-2 during the study but did not develop N-protein antibodies. The lack of a comparator arm of an approved mRNA vaccine limited comparison of the breadth of nAb responses induced by these vaccines to the breadth of responses induced by approved mRNA vaccines.

At baseline, spike-specific T cell responses were detectable in a majority of participants, due to previous vaccination, infection, or both. These baseline T cell responses were associated with the production of Th1-type of cytokine responses (including IFN-γ and/or IL-2 responses), and none of the assays identified any Th2-type responses (IL-4, IL-13 and IL-5). However, perhaps due to enrollment of non-naïve participants, vaccination with GRT-R910 was not associated with further increases in spike-specific T cell responses for most participants. One of the advantages of this product was intended to be the inclusion of TCE to induce TCE-specific responses. However, we did not observe significant increases in the cell mediated immune responses to overlapping peptides from the TCE cassettes spanning membrane, nucleocapsid, and open reading frame 3. In contrast, when one or two doses of GRT-R910 was administered as a booster vaccine to persons aged ≥60 years in the UK who had previously received a primary series with AZD1222, minimal changes in the magnitude of T cell responses to overlapping peptide pools assessed by IFN-γ ELISpot assay were observed [21]. It is possible that participants would have needed more than one vaccination with GRT-R910 encoding the TCE to induce optimal TCE-specific responses.

This trial experienced several limitations including small sample sizes, heterogeneity of the participants (history of SARS-CoV-2 infection, different numbers of prior approved COVID-19 vaccines doses received), half of the participants developing SARS-CoV-2 infection during the trial, and multiple participants receiving non-study COVID-19 vaccines during the trial. Small sample sizes especially in the per-protocol population diminished our ability to adequately evaluate immunogenicity at later time points, in particular, at Day 366. The lack of N-protein antibody data on Day 366 means that some participants may have developed SARS-CoV-2 infection between Days 181 and 366, and we did not capture these infections if the participants were unaware of them and did not self-report them.

Despite the limitations encountered, the results of this trial suggest that GRT-R910 can enhance pre-existing SARS-CoV-2 nAb responses in persons at the 3, 6, and 10 μg doses. These lower doses allow dose sparing, compared to the currently licensed mRNA-based vaccines. In some persons these nAb responses may persist for up to 6 months. However, in all future studies of SAM vaccines, the dose-sparing and durable immunogenicity of these products must be weighed against the potential for inducing increased rates of reactogenicity. This study and other SAM mRNA vaccine studies have demonstrated that these novel vaccines may provide the benefit of durable and broad immune responses [15,21,38,40] and the self-amplifying mRNA vaccine platform warrants ongoing development.

## Supplementary Material

MMC1

## Figures and Tables

**Table 1:** Participant characteristics at enrollment.

		Age 18–60 years		Age > 60 years		
			
		Group 5 3 μg GRT-R910 (*N* = 10)	Group 6 6 μg GRT-R910 (*N* = 10)	Group 9 3 μg GRT-R910 (*N* = 8)	Group 10 6 μg GRT-R910 (N = 10)	Group 11 10 μg GRT-R910 (N = 10)
						
Demographic Category	Characteristic	n	n	n	n	n

**Sex**	Male	4	3	3	4	3
	Female	6	7	5	6	7
**Mean (standard deviation) age in years**		32.9 (12.6)	34 (8.7)	63.4 (2.8)	67.8 (4.7)	66.0 (4.5)
**Ethnicity**	Not Hispanic or Latino	10	9	8	10	10
	Hispanic or Latino	–	1	–	–	–
**Race**	American Indian or Alaskan Native	–	1	–	–	–
	Asian	1		–	–	–
	White	9	9	8	10	10
**Previous SARS-CoV-2 Infection by serology and history**	Previous infection	–	–	–	2	2
**Previous COVID-19 vaccinations**	Primary mRNA vaccination (2 doses) only	10	10	8	–	–
	Primary mRNA vaccination (2 doses) + mRNA booster	–	–	–	10	10

**Table 2:** Analysis populations by vaccination arm.

		Group 5: 3 μg GRT-R910 (N = 10)		Group 6: 6 μg GRT-R910 (N = 10)		Group 9: 3 μg GRT-R910 (N = 8)		Group 10: 6 μg GRT-R910 (N = 10)		Group 11: 10 μg GRT-R910 (N = 10)	
					
Analysis Populations	Reason Participants Excluded	n	%	n	%	n	%	n	%	n	%

**Safety Population**	Included	10	100	10	100	8	100	10	100	10	100
	Excluded from individual time points: Any Reason	–	–	–	–	–	–	–	–	–	–
**Modified Intent-To-Treat Population**	Included	10	100	10	100	8	100	10	100	10	100
	Excluded from individual time points: Any Reason	–	–	–	–	–	–	–	–	–	–
**Per Protocol Population**	Included in at least 1 time point	10	100	10	100	8	100	10	100	10	100
	Excluded from individual time points: Any Reason	7	70	9	90	6	75	9	90	6	60
	Breakthrough COVID Infection^a, c^	5	50	7	70	5	63	6	60	1	10
	Receipt of Non-Study COVID-19 Vaccination^a^	2	20	1	10	1	13	2	20	3	30
	Visit Out of Window^b^	–	–	1	10	–	–	1	10	2	20

Note: N = Number of participants enrolled. n = Number of participants meeting the criteria.

a Participant data are excluded from the per protocol analyses at all time points that occur at or after this protocol deviation. Only one exclusion reason is included per participant.

Participant data collected up to the time the exclusionary criterion is met is eligible for analysis.

b Only data from the out of window visit are excluded from the per protocol analyses.

c Breakthrough COVID infection is defined as either self-report of COVID infection or development of N-protein antibody positivity after enrollment.

**Table 3:** Summary of pseudovirus neutralizing antibody test ID_50_ against SARS-CoV-2 D614G, per-protocol population.

Time Point	Statistic	Group 5: 3 μg GRT-R910 (*N* = 10)	Group 6: 6 μg GRT-R910 (N = 10)	Group 9: 3 μg GRT-R910 (*N* = 8)	Group 10: 6 μg GRT-R910 (N = 10)	Group 11: 10 μg GRT-R910 (N = 10)

**Day 1 (Pre-Vaccination)**	n	10	10	8	10	10
	GMT (95% CI)	112.8 (75.0, 169.8)	405.4 (163.7, 1003.9)	102.8 (43.7, 242.0)	482.9 (137.4, 1697.1)	375.5 (190.3, 740.9)
	Seropositive (95% CI)	100 (69, 100)	100 (69, 100)	100 (63, 100)	100 (69, 100)	100 (69, 100)
**Day 15 Post Vaccination**	n	10	9	8	10	9
	GMT (95% CI)	376.1 (235.0, 601.9)	940.5 (417.7, 2117.5)	682.4 (360.8, 1290.6)	921.6 (346.0, 2454.7)	734.0 (387.3, 1391.2)
	GMFR (95% CI)	3.3 (1.6, 6.9)	2.53 (1.0, 6.5)	6.6 (2.0, 22.0)	1.9 (1.2, 3.1)	2.15 (1.0, 4.7)
	Seropositive (95% CI)	100 (69, 100)	100 (66, 100)	100 (63, 100)	100 (69, 100)	100 (66, 100)
**Day 29 Post Vaccination**	n	9	10	8	9	10
	GMT (95% CI)	565.2 (285.5, 1119.2)	1468.7 (530.3, 4067.8)	829.8 (390.3, 1764.0)	1192.6 (461.7, 3080.4)	1229.7 (688.4, 2196.6)
	GMFR (95% CI)	5.2 (2.1, 13.3)	3.6 (1.3, 10.4)	8.1 (2.1, 31.4)	2.7 (1.1, 6.7)	3.3 (1.5, 7.4)
	Seropositive (95% CI)	100 (66, 100)	100 (69, 100)	100 (63, 100)	100 (66, 100)	100 (69, 100)
**Day 85 Post Vaccination**	n	10	10	6	9	9
	GMT (95% CI)	559.7 (298.3, 1050.2)	1371.6 (454.3, 4141.4)	654.8 (335.6, 1277.8)	1877.6 (1182.8, 2980.8)	1726.8 (916.0, 3255.3)
	GMFR (95% CI)	5.0 (2.1, 11.7)	3.38 (1.0, 11.3)	5.8 (1.2, 28.1)	2.7 (1.1, 7.1)	4.2 (1.9, 9.1)
	Seropositive (95% CI)	100 (69, 100)	100 (69, 100)	100 (54, 100)	100 (66, 100)	100 (66, 100)
**Day 181 Post Vaccination**	n	5	5	4	6	10
	GMT (95% CI)	709.6 (423.0, 1190.5)	527.5 (130.9, 2125.9)	562.5 (378.5, 835.9)	1867.9 (966.2, 3611.1)	1229.8 (675.5, 2238.8)
	GMFR (95% CI)	6.1 (2.8, 13.2)	2.4 (0.2, 33.0)	8.2 (1.2, 57.5)	1.8 (0.5, 6.7)	3.3 (1.3, 8.0)
	Seropositive (95% CI)	100 (48, 100)	100 (48, 100)	100 (40, 100)	100 (54, 100)	100 (69, 100)
**Day 366 Post Vaccination**	n	2	1	2	1	6
	GMT (95% CI)	3529.1 (700.1, 17790.5)	3694.8 (NE)	187.1 (11.0, 3189.0)	648.3 (NE)	1516.5 (628.5, 3659.1)
	GMFR (95% CI)	23.8 (14.2, 39.8)	9.42 (NE)	4.37 (4.02, 4.74)	1.99 (NE)	3.15 (0.8, 11.8)
	Seropositive (95% CI)	100 (16, 100)	100 (3,100)	100 (16, 100)	100 (3, 100)	100 (54, 100)

Notes: N = Number of participants enrolled.

n = Number of participants in the Per Protocol population with available results.

NE = Not Estimable.

GMT = Geometric Mean Titer, GMFR = Geometric Mean Fold Rise.

**Table 4:** Summary of focus reduction neutralization test ID_50_ against SARS-CoV-2 D614G, per protocol population.

Time Point	Statistic	Group 5: 3 μg GRT-R910 (N = 10)	Group 6: 6 μg GRT-R910 (N = 10)	Group 9: 3 μg GRT-R910 (N = 8)	Group 10: 6 μg GRT-R910 (N = 10)	Group 11: 10 μg GRT-R910 (N = 10)

**Day 1 (Pre-Vaccination)**	n	10	10	8	10	10
	GMT (95% CI)	77.3 (47.6, 125.7)	243.9 (118.5, 502.3)	84.2 (34.1, 207.7)	325.3 (95.6, 1107.1)	431.5 (209.0, 891.0)
	Seropositive (95% CI)	100 (69, 100)	100 (69, 100)	88 (47, >99)	90 (55, >99)	100 (69, 100)
**Day 15 Post Vaccination**	n	10	9	8	10	9
	GMT (95% CI)	291.9 (183.3, 465.0)	486.8 (226.4, 1046.8)	553.0 (315.9, 968.2)	560.1 (265.0, 1184.0)	784.8 (408.5, 1507.9)
	GMFR (95% CI)	3.8 (2.0, 7.3)	2.1 (0.9, 5.2)	6.6 (2.2, 20.1)	1.7 (1.0, 2.9)	2.2 (1.1, 4.1)
	Seropositive (95% CI)	100 (69, 100)	100 (66, 100)	100 (63, 100)	100 (69, 100)	100 (66, 100)
**Day 29 Post Vaccination**	n	9	10	8	9	10
	GMT (95% CI)	448.1 (219.4, 915.4)	824.4 (410.4, 1656.1)	780.3 (449.1, 1355.6)	653.2 (321.1, 1328.6)	1505.6 (897.4, 2526.0)
	GMFR (95% CI)	6.23 (2.6, 14.8)	3.4 (1.5, 7.9)	9.27 (3.0, 28.9)	2.4 (1.1, 5.0)	3.5 (1.8, 6.9)
	Seropositive (95% CI)	100 (66, 100)	100 (69, 100)	100 (63, 100)	100 (66, 100)	100 (69, 100)
**Day 85 Post Vaccination**	n	10	10	6	9	9
	GMT (95% CI)	427.1 (236.0, 772.9)	790.5 (332.2, 1880.8)	730.3 (407.0, 1310.6)	946.0 (613.9, 1457.8)	1297.8 (783.4, 2149.8)
	GMFR (95% CI)	5.5 (2.7, 11.5)	3.2 (1.2, 9.1)	8.5 (1.4, 50.4)	2.0 (0.9, 4.5)	2.86 (1.5, 5.6)
	Seropositive (95% CI)	100 (69, 100)	100 (69, 100)	100 (54, 100)	100 (66, 100)	100 (66, 100)
**Day 181 Post Vaccination**	N	5	5	4	6	10
	GMT (95% CI)	664.7 (218.0, 2026.9)	305.4 (153.8, 606.5)	509.6 (287.3, 903.7)	1102.3 (538.0, 2258.5)	1078.7 (597.9, 1946.3)
	GMFR (95% CI)	8.9 (2.5, 31.6)	1.6 (0.2, 14.0)	8.67 (0.8, 95.8)	1.5 (0.6, 3.3)	2.5 (1.2, 5.2)
	Seropositive (95% CI)	100 (48, 100)	100 (48, 100)	100 (40, 100)	100 (54, 100)	100 (69, 100)
**Day 366 Post Vaccination**	n	2	1	2	1	6
	GMT (95% CI)	2241.6 (0.2, 24507301.1)	2706.1 (NE)	313.6 (78.7, 1248.9)	1305.9 (NE)	1360.8 (545.3, 3396.3)
	GMFR (95% CI)	17.9 (0.2, 2172.1)	5.4 (NE)	7.1 (5.9, 8.6)	4.0 (NE)	2.4 (0.7, 7.9)
	Seropositive (95% CI)	100 (16, 100)	100 (3, 100)	100 (29, 100)		

Notes: N = Number of participants enrolled.

n = Number of participants in the Per Protocol population with available results.

NE = Not Estimable.

GMT = Geometric Mean Titer, GMFR = Geometric Mean Fold Rise.

## Data Availability

Data will be made available on request.

## Appendix A. Supplementary data

Supplementary data to this article can be found online at https://doi.org/10.1016/j.vaccine.2026.128358.
