# Supplementary material for: The safety, reactogenicity, and immunogenicity of the self-amplifying mRNA COVID-19 vaccine GRT-R910 as a booster in healthy adults
Source: Vaccine. Author manuscript; Available in PMC 2026 May 31. (PMC13222540; doi:10.1016/j.vaccine.2026.128358)
Supplement: MMC1 [file NIHMS2151674-supplement-MMC1.docx]

Supplemental Appendix to Manuscript Entitled

**The Safety, Reactogenicity, and Immunogenicity of the Self-amplifying mRNA COVID-19 Vaccine GRT-R910 as a Booster in Healthy Adults**

**Contents**

[**DMID 20-0034 Study Group** 4](#_Toc208934290)

[**DMID 20-0034 Study Team Members** 6](#_Toc208934291)

**Overall Study Design and** [**Subject Eligibility Criteria** 8](#_Toc208934292)

[**Laboratory Methods:** 13](#_Toc208934293)

[Enzyme-linked immunosorbent assay (ELISA) Assays 13](#_Toc208934294)

[Serum Neutralization Assays 13](#_Toc208934295)

[Pseudovirus-based Neutralization Assays 13](#_Toc208934296)

[Focus Reduction Neutralization Test (FRNT) 13](#_Toc208934297)

[**Cellular Immunogenicity Assays** 14](#_Toc208934298)

[IFNγ Enzyme-linked immunosorbent assay (ELISpot) assays 16](#_Toc208934299)

[Meso Scale Discovery (MSD) U-plex assay 17](#_Toc208934300)

[Intracellular Cytokine Staining (ICS) Assays 18](#_Toc208934301)

[**Supplementary Tables** 20](#_Toc208934302)

[Supplementary Table 1. Study Enrollment for All Study Groups 21](#_Toc208934303)

Supplementary Table 2. Reagents for Intracellular Cytokine Staining Assays 22

[Supplementary Table 3. Numbers of participants who testing positive for Nucleocapsid (N)-antibody during the course of the study 223](#_Toc208934305)

[Supplementary Table 4. New onset chronic medical conditions (NOCMC) occurring during the study 24](#_Toc208934306)

[Supplementary Table 5. Summary of pseudovirus neutralizing antibody test ID_50_ responses against SARS-CoV-2 BA.1, per-protocol population 25](#_Toc208934307)

[Supplementary Table 6. Summary of pseudovirus neutralizing test ID_50_ responses against SARS-CoV-2 BA.4/5, per-protocol population 26](#_Toc208934308)

[Supplementary Table 7. Summary of focus reduction neutralization test ID_50_ against SARS-CoV-2 BA.1, per protocol population 27](#_Toc208934309)

[Supplementary Table 8. Summary of focus reduction neutralization test ID_50_ against SARS-CoV-2 BA.4/5, per protocol population 28](#_Toc208934310)

[Supplementary Table 9. Summary of ELISA IgG antibody responses against SARS-CoV-2 S-2P, per-protocol population 29](#_Toc208934311)

[Supplementary Table 10: Summary of ELISA IgG antibody responses against SARS-CoV-2 RBD, per-protocol population 31](#_Toc208934312)

[**Supplemental Figures** 32](#_Toc208934313)

[Supplemental Figure 1: Clinical Laboratory Results by Maximum Severity Post Baseline, 18‑60 years of age 32](#_Toc208934314)

[Supplemental Figure 2: Clinical Laboratory Results by Maximum Severity Post Baseline, >60 years of age 33](#_Toc208934315)

[Supplementary Figure 3. Focus reduction neutralization test ID50 against SARS-CoV-2 D614G, 18-60 years of age, per-protocol population 34](#_Toc208934316)

[Supplementary Figure 4. Focus reduction neutralization test ID_50_ against SARS-CoV-2 D614G, >60 years of age, per-protocol population 35](#_Toc208934317)

[Supplemental Figure 5. Distribution of ELISA IgG Antibody Against SARS-CoV-2 S-2P, 18-60 years of age, per-protocol population 36](#_Toc208934318)

[Supplemental Figure 6. Distribution of ELISA IgG antibody against SARS-CoV-2 S-2P, >60 years of age, per-protocol population 37](#_Toc208934319)

[Supplemental Figure 7. Distribution of ELISA IgG antibody against SARS-CoV-2 RBD, 18-60 years of age, per-protocol population 38](#_Toc208934320)

[Supplemental Figure 8. Distribution of ELISA IgG antibody against SARS-CoV-2 RBD, >60 years of age, per-protocol population 39](#_Toc208934321)

[Supplementary Figure 9. Distribution of T Cells expressing IFNγ as measured by the ELISpot assay in spot forming units (SFU) per million, stimulated with Spike, 18-60 years of age, per-protocol population 40](#_Toc208934322)

[Supplementary Figure 10. Distribution of T Cells expressing IFNγ as measured by the ELISpot assay in spot forming units (SFU) per million, stimulated with Spike, >60 years of age, per-protocol population 41](#_Toc208934323)

[Supplementary Figure 11. Distribution of T Cells expressing IFNγ as measured by the ELISpot assay in spot forming units (SFU) per million, stimulation with 15mer OLP (overlapping peptides) spanning membrane, nucleocapsid, and open reading frame, 18-60 years of age, per-protocol population 42](#_Toc208934324)

[Supplementary Figure 12. Distribution of T Cells expressing IFNγ as measured by the ELISpot assay in spot forming units (SFU) per million, stimulation with 15mer OLP (overlapping peptides) spanning membrane, nucleocapsid, and open reading frame, >60 years of age, per-protocol population 43](#_Toc208934325)

[Supplementary Figure 13. Distribution of CD4 T Cells Expressing IFNγ and/or IL-2, stimulation with Spike peptides, 18-60 years of age, per-protocol population as measured by intracellular cytokine staining 44](#_Toc208934326)

[Supplementary Figure 14. Distribution of CD4 T Cells Expressing IFNγ and/or IL-2, stimulation with Spike peptides, >60 years of age, per-protocol population as measured by intracellular cytokine staining 45](#_Toc208934327)

[Supplemental Figure 15. Distribution of CD4 T cells expressing IFNγ and/or IL-2, stimulation with any TCE, 18-60 years of age, per-protocol population as measured by intracellular cytokine staining 46](#_Toc208934328)

[Supplemental Figure 16. Distribution of CD4 T cells expressing IFNγ and/or IL-2, stimulation with any TCE, >60 years of age, per-protocol population as measured by intracellular cytokine staining 47](#_Toc208934329)

[Supplemental Figure 17. Distribution of CD8 T cells expressing IFNγ and/or IL-2, stimulation with any TCE, 18-60 years of age, per-protocol population as measured by intracellular cytokine staining 48](#_Toc208934330)

[Supplemental Figure 18. Distribution of CD8 T cells expressing IFNγ and/or IL-2, stimulation with any TCE, >60 years of age, per-protocol population as measured by intracellular cytokine staining 49](#_Toc208934331)

[Supplementary Figure 19. Distribution of CD4 T cells expressing IL-4 or IL-5 or IL-13 and CD154, stimulation with Spike peptides, 18-60 years of age, per-protocol population as measured by intracellular cytokine staining 50](#_Toc208934332)

[Supplementary Figure 20. Distribution of CD4 T cells expressing IL-4 or IL-5 or IL-13 and CD154, stimulation with Spike peptides, >60 years of age, per-protocol population as measured by intracellular cytokine staining 51](#_Toc208934333)

[**Supplementary References** 52](#_Toc208934334)

# **DMID 20-0034 Study Group**

**St. Louis University, St. Louis, MO, Vaccine Treatment and Evaluation Unit (VTEU)**

Daniel F. Hoft, MD, PhD; Getahun Abate, MD, PhD; Tracy Montauk, RN; Sabrina DiPiazza, RN, NP; Azra Bazevic DVM, MPH

**Emory University, Atlanta, GA, Vaccine Treatment and Evaluation Unit (VTEU)**

Paulina A. Rebolledo, MD, MS; Nadine Rouphael, MD; Zanthia Wiley, MD; Srilatha Edupuganti, MD; Matthew H. Collins, MD; Daniels S. Graciaa, MD; Cassie Grimsley-Ackerley, MD; Colleen F. Kelley, MD; Varun K, Phadke, MD; Meredith Davis-Gardner; Bushra Wali, PhD; Madison Ellis; Lilin Lai, MD; Alberto Moreno, MD

**University of Washington, Seattle, WA, Vaccine Treatment and Evaluation Unit (VTEU)**

Tara M. Babu, MD, MSCI; Anna Wald, MD, MPH; David M. Koelle, MD; Kirsten Hauge, MPH; Jessica Heimonen, MPH; Jina Taub, ARNP; Dana Varon, ARNP; Britt Murphy, ARNP; Morissa Pertik, PA-C; Alyssa Braun, BS; Jessica Morena, BS; Lindsey McClellan, MPH; Amy Link, BS; Chloe Wilkens, BS; Taylor Krause, BS; Matt Seymour, MP; Lawrence Hemingway, BS; Jean Mernaugh, BS; Chris McClurkan, BS; Kerry J. Laing, PhD; Meredith Potochnic, PharmD; Joong Kim, PharmD; April Cowgil, PharmD; Bao-Chao Vo, Mark Drummond, BA, Nui Pholsena, ARNP

**Baylor College of Medicine, Houston, TX, Vaccine Treatment and Evaluation Unit (VTEU)**

Jennifer A. Whitaker, MD, MS; Hana M. El Sahly, MD; Robert L. Atmar, MD; C. Mary Healy, MD; Wendy A. Keitel, MD; Christine Akamine, MD

**Emmes Group**

Mat Makowski, PhD; Jinjian Mu, PhD; Jennifer Theusch MT(ASCP), MBS, CCRC; Andrea Carmack, MS; Kimberly Bowers

**Seattle Children’s Research Institute, Seattle, WA**

Rhea N. Coler, MSc, PhD

**Division of Microbiology and Infectious Diseases (DMID), National Institute of Allergy and Infectious Diseases (NIAID), National Institutes of Health (NIH), Bethesda, MD**

Paul C. Roberts^,^ PhD; Mamodikoe Makhene MD; Melinda Tibbals

**Duke University Laboratory**, **Durham, NC**

David Montefiori, Ph.D., Amanda Eaton, Ph.D.

**Fred Hutch Seattle, WA**

Christine M. Posavad, PhD; M. Juliana McElrath, MD, PhD; Stephen C. De Rosa, MD

**FHI 360, Durhan, NC**

Janet I. Archer, MSc

**Gritstone bio, Inc.**

Karin Jooss, PhD; Meghan Hart, ALM; Laura Veckerelli, BFA; Harshni Venkatraman, MS

# **DMID 20-0034 Study Team Members**

**St. Louis University, St. Louis, MO, Vaccine Treatment and Evaluation Unit (VTEU)**

Daniel F. Hoft, MD, PhD; Getahun Abate, MD, PhD; Tracy Montauk, RN; Sabrina DiPiazza, RN, NP; Azra Bazevic DVM, MPH

**Emory University, Atlanta, GA, Vaccine Treatment and Evaluation Unit (VTEU)**

Paulina A. Rebolledo, MD, MS; Nadine Rouphael, MD; Zanthia Wiley, MD; Srilatha Edupuganti, MD; Matthew H. Collins, MD; Daniels S. Graciaa, MD; Cassie Grimsley-Ackerley, MD; Colleen F. Kelley, MD; Varun K, Phadke, MD; Meredith Davis-Gardner; Bushra Wali, PhD; Madison Ellis; Lilin Lai, MD; Alberto Moreno, MD

**University of Washington, Seattle, WA, Vaccine Treatment and Evaluation Unit (VTEU)**

Tara M. Babu, MD, MSCI; Anna Wald, MD, MPH; David M. Koelle, MD; Kirsten Hauge, MPH; Jessica Heimonen, MPH; Jina Taub, ARNP; Dana Varon, ARNP; Britt Murphy, ARNP; Morissa Pertik, PA-C; Alyssa Braun, BS; Jessica Morena, BS; Lindsey McClellan, MPH; Amy Link, BS; Chloe Wilkens, BS; Taylor Krause, BS; Matt Seymour, MP; Lawrence Hemingway, BS; Jean Mernaugh, BS; Chris McClurkan, BS; Kerry J. Laing, PhD; Meredith Potochnic, PharmD; Joong Kim, PharmD; April Cowgil, PharmD; Bao-Chao Vo, Mark Drummond, BA, Nui Pholsena, ARNP

**Baylor College of Medicine, Houston, TX, Vaccine Treatment and Evaluation Unit (VTEU)**

Jennifer A. Whitaker, MD, MS; Hana M. El Sahly, MD; Robert L. Atmar, MD; C. Mary Healy, MD; Wendy A. Keitel, MD; Christine Akamine, MD; Janet Brown, RPH, JD; Cathy Faw, RPh; Brandie Philips, RN; Dawn Turner, RN; Chianti Wade-Bowers, RN; Jenny Patel, RN; Chanei Henry; Connie Rangel, RN; Pedro Piedra, MD; Lisreina Toro; Yolanda Rayford; Kayla Burrell; Jesus Banay; Maria Shlyapobersky

**Emmes Group**

Mat Makowski, PhD; Jinjian Mu, PhD; Jennifer Theusch MT(ASCP), MBS, CCRC; Andrea Carmack, MS; Kimberly Bowers

**Seattle Children’s Research Institute, Seattle, WA**

Rhea N. Coler, MSc, PhD

**Division of Microbiology and Infectious Diseases (DMID), National Institute of Allergy and Infectious Diseases (NIAID), National Institutes of Health (NIH), Bethesda, MD.**

Paul C. Roberts^,^ PhD; Mamodikoe Makhene MD; Melinda Tibbals

**Duke University Laboratory**, **Durham, NC**

David Montefiori, PhD, Amanda Eaton, PhD

**Fred Hutch Seattle, WA**

Christine M. Posavad, PhD; M. Juliana McElrath, MD, PhD, Stephen De Rosa, MD

**FHI 360, Durham, NC**

Janet I. Archer, MSc; Khadaijah Wiley

**Gritstone bio, Inc.**

Karin Jooss, PhD; Meghan Hart, ALM; Laura Veckerelli, BFA; Harshni Venkatraman, MS; Martina Marrali, MSc; Sonia Kounlavouth, BS; Jason Jaroslavsky, MS; Lorenzo Hernandez, BS; Enrique Podaza, PhD

# **Overall Study Design and Subject Eligibility Criteria**

**Overall Study Design:**

This was a multi-center, open-label, dose-escalation, non-randomized study in healthy adults of the safety and reactogenicity (primary endpoint) and immunogenicity (secondary endpoint) of several investigational COVID-19 vaccines produced by Gritstone (NCT04776317) (Supplemental Table 1). These vaccines included investigational chimpanzee adenoviral vector (ChAd) COVID-19 vaccines GRT-C907 (ChAd-S), GRT-C909 (ChAd-S-TCE) and SAM COVID-19 vaccines GRT-R908 (SAM-S) and GRT-R910 (SAM-S-TCE). Homologous and heterologous prime-boost vaccination schedules of GRT-C907, GRT-R908, and GRT-R910 were evaluated among SARS-CoV-2 infection- and vaccine-naïve adults in Groups 1-4 in Stage 1 (Supplementary Table 1). In Stage 1, GRT-R908 doses were found to be highly reactogenic at the 30 µg dose, which prompted re-design of the study, starting at lower doses in stage 2. Due to the ever-changing vaccine recommendations during the COVID-19 pandemic, decreased population of potential SARS-CoV-2 naïve participants, and the reactogenicity associated with the higher doses of GRT-R908 in stage 1, the study shifted to focus on these platforms as booster vaccines. In stage 2, single-dose boosters of GRT-R910 were evaluated among persons aged 18-60 years (Groups 5 and 6) and over 60 years (Groups 9-11) who had previously completed an approved mRNA COVID-19 vaccine series (Table 1). In addition, single-dose boosters of GRT-C909 (ChAd-S-TCE) were evaluated in adults over 60 years of age who had previously completed an approved mRNA COVID-19 vaccine series in Groups 13-15 (Supplementary Table 1). These vaccinations were limited to older adults due to emerging concerns of potential adverse effects including vaccine-induced immune thrombotic thrombocytopenia, observed in younger adults given other adenoviral vectored COVID-19 vaccines. Results from Stage 1 cohorts and the Stage 2 GRT-C909 cohorts can be found at <https://clinicaltrials.gov/study/NCT04776317>.

**Inclusion Criteria:**

Subjects eligible to participate in this trial must meet all of the following inclusion criteria:

1. Provide written informed consent prior to initiation of any study procedures.

2. Able and willing (in the investigator’s opinion) to comply with all study requirements.

3. Are men or non-pregnant women aged 18 years or older at enrollment.

4. Are in good health*.

**As defined by absence of clinically significant medical conditions defined by the CDC as increasing risk for severe COVID-19 disease (see exclusion criteria), or other acute or chronic medical conditions determined by medical history, physical examination (PE), screening laboratory test results, and/or clinical assessment of the investigator that are either listed as exclusion criteria below or in the opinion of the investigator would increase risk for study participation or affect the assessment of the safety of subjects. Chronic medical conditions should be stable for the last 60 days (no hospitalizations, emergency room or urgent care for condition, or invasive medical procedures). Any prescription change that is due to change of health care provider, insurance company, etc., or done for financial reasons, and in the same class of medication, will not be considered a deviation of this inclusion criterion. Any change in prescription medication due to improvement* *of a disease outcome, as determined by the participating site PI or appropriate sub- investigator, will not be considered a deviation of this inclusion criterion. Subjects may be on chronic or as needed (prn) medications if, in the opinion of the participating site PI or appropriate sub-investigator, they pose no additional risk to subject safety or assessment of reactogenicity and immunogenicity, and do not indicate a worsening of medical diagnosis/condition. Similarly, medication changes in the 60 days prior to enrollment as well as subsequent to enrollment and study vaccination are acceptable provided the change was not precipitated by deterioration in the chronic medical condition, and there is no anticipated additional risk to the subject or interference with the evaluation of responses to study vaccination.*

5. Agree to refrain from blood donation during the course of the study.

6. Plan to remain living in the area for the duration of the study.

7. Women of childbearing potential (WOCBP)* must plan to avoid pregnancy for at least 60 days after the last study vaccination and be willing to use an adequate method of contraception** consistently for 30 days prior to first study vaccine and for at least 60 days after the last study vaccine.

**Not sterilized via bilateral oophorectomy, tubal ligation/salpingectomy, hysterectomy, or successful Essure® placement (permanent, non-surgical, non-hormonal sterilization with documented radiological confirmation test at least 90 days after the procedure); still menstruating; or < 1 year has passed since the last menses if menopausal.*

***Acceptable methods of birth control include the following: oral contraceptives, injection hormonal contraceptive, implant hormonal contraceptive, hormonal patch, intrauterine device, spermicidal products and barrier methods (such as cervical sponge, diaphragm, or condom with spermicide), abstinence, monogamous with a vasectomized partner, non-male sexual relationship.*

8. Women of childbearing potential must have a negative urine or serum pregnancy test within 24 hours prior to each study vaccination.

9. Vital signs within acceptable ranges:

• Pulse >50 and ≤100 beats per minute

• Systolic blood pressure (BP) ≤140 millimeters of mercury (mmHg)

• Diastolic BP ≤90 mmHg

• Oral temperature <37.8°C (100.0°F)

10. Clinical screening lab evaluations (white blood cell (WBC), hemoglobin (HgB), platelets (PLT), alanine aminotransferase (ALT), aspartate aminotransferase (AST), alkaline phosphatase (ALP), total bilirubin (T Bili), creatine kinase (CK), serum creatinine (Cr) and prothrombin time (PT)/partial thromboplastin time (PTT)) are within acceptable normal reference ranges at the clinical lab being used*.

**With the exception that ALT, AST, ALP, and creatinine values that are below the reference range will not be exclusionary as these values below reference range are clinically insignificant. Any other screening lab value outside the reference range that is thought to be clinically insignificant by a site investigator must be discussed with the DMID Medical Officer prior to enrollment.*

11. Must agree to genetic testing and storage of samples for secondary research.

12. Received at least 2 doses of EUA/licensed mRNA vaccines or at least 1 dose of Ad26 vaccine followed by an mRNA booster, with the last COVID-19 vaccine dose given at least 112 days prior to enrollment (Stage 2 only), as confirmed via CDC vaccination card or other appropriate documentation. Subjects may or may not have been previously infected with SARS-CoV-2.*

*In version 7.0 of the protocol, we prioritize enrollments of subjects who have either received 3 EUA/licensed mRNA vaccinations (SAM-S-TCE single boosting Groups 7A, 7B, 10A, 10B, 11A, 11B, 12A and 12B, and ChAd-S-TCE single boosting Groups 13-15) or 1-2 doses of EUA/licensed Ad26 prior to 1 dose of EUA/licensed mRNA (SAM-S-TCE double boosting Groups 8A, 8B, 12A and 12B).

**Exclusion Criteria:**

Subjects eligible to participate in this trial must not meet any of the following exclusion criteria:

1. History of prior confirmed (PCR or antigen test positive) COVID-19 less than 112 days prior to enrollment.

2. Positive for anti-nucleoprotein SARS-CoV-2 specific antibody by enzyme-linked immunosorbent assay (ELISA) and had the history of upper respiratory illness (URI) compatible with COVID-19 during the 112 days prior to enrollment (seropositivity without a history of URI during the 112 days prior to enrollment will be considered remotely infected persons eligible for enrollment).

3. Positive nasal swab polymerase chain reaction (PCR) at screening.

4. Body mass index (BMI) > 30 kg/m2 for Stage 1 participants and BMI > 35 kg/m2 for Stage 2 participants.

5. Presence of medical comorbidities that would place the subject at increased risk for severe COVID-19*.

**Chronic kidney disease, chronic lung disease (including moderate-to-severe asthma), chronic heart disease (heart failure, coronary artery disease or cardiomyopathies), cerebrovascular disease, diabetes mellitus, chronic liver disease, sickle cell disease.*

6. Increased risk of occupational exposure to SARS-CoV-2 (healthcare workers and emergency response personnel)*.

**Applies to Stage 1 participants only.*

7. Prior receipt of an approved/licensed or investigational SARS-CoV-2 vaccine (including under EUA)*, approved or investigational adenovirus-vectored vaccines**, or any other approved or investigational vaccine likely to impact the interpretation of the trial data.

**Exclusion of prior receipt of EUA/licensed COVID-19 vaccines applies to Stage 1 participants only.*

***With the exception of prior receipt of EUA Johnson & Johnson/Janssen Ad 26 COVID-19 vaccine which is permitted for Groups 8 and 12.*

8. On current treatment or prevention agents with activity against SARS-CoV-2.

9. Current smoking or vaping or history of smoking or vaping in prior year*.

**Applies to Stage 1 participants only.*

10. Breastfeeding, pregnant, or planning to become pregnant during the course of the study.

11. Participation in another research study involving receipt of an investigational product in the 60 days preceding enrolment or planned use during the study period.

12. Receipt or planned receipt of any live, attenuated vaccine within 28 days before or after study vaccination.

13. Receipt or planned receipt of any subunit or killed vaccine within 14 days before or after vaccination.

14. Administration of immunoglobulins and/or any blood products within the three months preceding the planned administration of first study vaccination or at any time during the study.

15. Any confirmed or suspected immunosuppressive or immunodeficient state, including human immunodeficiency virus (HIV) infection, asplenia, recurrent, severe infections and chronic (more than 14 continuous days) immunosuppressant medication within the past 6 months (inhaled, ophthalmic, and topical steroids are allowed).

16. History of allergic disease or reactions likely to be exacerbated by any component of the vaccine, including urticaria, respiratory difficulty or abdominal pain (or any immediate allergic reaction of any severity to polysorbate due to potential cross-reactive hypersensitivity with the polyethylene glycol component of the vaccine).

17. Any history of hereditary angioedema, acquired angioedema, or idiopathic angioedema.

18. Any history of anaphylaxis, including but not limited to reaction to vaccination.

19. Any history of severe allergic drug reaction.

20. History of cancer (except basal cell carcinoma of the skin and cervical carcinoma in situ).

21. History of serious ongoing, unstable psychiatric condition that in the opinion of the investigator would interfere with study participation.

22. Seizure in the past 3 years or treatment for seizure disorder in the past 3 years.

23. Bleeding disorder (e.g., factor deficiency, coagulopathy, or platelet disorder), or prior history of significant bleeding or bruising following IM injections or venipuncture or family history of bleeding disorder.

24. Recent (within the past 3 months) surgery, immobility, chronic infection, or head trauma that could increase the risks of thrombosis.

25. Suspected or known current alcohol abuse. Suspected or known drug abuse in the 5 years preceding enrollment.

26. Seropositive for HIV, hepatitis B surface antigen (HBsAg), or seropositive for hepatitis C virus (antibodies to HCV).

27. Have an acute illness* within 72 hours prior to study vaccination.

**An acute illness which is nearly resolved with only minor residual symptoms remaining is allowable if, in the opinion of the site PI or appropriate sub-investigator, the residual symptoms will not interfere with the ability to assess safety parameters as required by the protocol.*

28. History of venous or arterial thrombosis or any known thrombophilic condition including heparin-induced thrombocytopenia (HIT) or family history of thrombosis.

29. History of myocarditis or pericarditis.

30. History of Guillain-Barré Syndrome (GBS).

31. Receiving heparin treatment or on medications associated with increased risk of bleeding or thrombosis.

32. Any other condition that in the opinion of the investigator would pose a health risk to the participant if enrolled or could interfere with evaluation of the trial vaccine or interpretation of study results.

# **Laboratory Methods:**

## **Enzyme-linked immunosorbent assay (ELISA) Assays**

ELISAs were performed using a qualified high throughput assay as previously described [1]. Sterile High Binding 384 well plates (Corning, Corning, NY) were filled with ELISA coating buffer (eBioscience, Inc. San Diego, CA) containing SARS-CoV-2 Spike or RBD antigen (Institute for Protein Design, Seattle WA) with a final concentration of 1 µg/ml. Plates were incubated for at least 2 hours at room temperature, or up to 3 days at 4°C. Plates were blocked with Blocking buffer (1% (w/v) BSA in PBS with 0.05% Tween) for at least 2 hours or wrapped in plastic wrap and incubated at 4°C overnight. Next, serum samples were diluted 1:100 in diluent (0.1% BSA in PBS with 0.05% Tween) and subsequently diluted 1:5. Plates were incubated overnight at 4°C. Plates were incubated 1 hour in the dark with HRP conjugated rec-Protein G antibodies (Invitrogen, 101223) diluted by a factor of 4000. Plates then received Tetramethylbenzidine (TMB) (SeraCare, Milford, MA) for 2.5 minutes, then the reaction was stopped with 1N H_2_SO_4_ (Sigma, St. Louis, MO). Plates were read at a wavelength of 450 nm with a reference filter set at 570 nm using a SpectraMax iD3 plate reader (Molecular Devices, San Jose, CA) and SoftMax Pro 7.1.2 analysis software. Endpoint titer (EPT) values were calculated using a minimum cutoff value set by assay standard negative controls and a 4-parameter logistic model in XL-fit software (model 208, Microsoft Excel).

## **Serum Neutralization Assays**

### **Pseudovirus-based Neutralization Assays**

Serum neutralizing antibodies were measured in a validated pseudovirus-based assay as a function of reductions in luciferase reporter gene expression after a single round of infection with either SARS-CoV-2.D614G, or SARS-CoV-2.BA.4/BA.5 spike-pseudotyped virus in 293T/ACE2 cells (293T cell line stably overexpressing the human ACE2 cell surface receptor protein, obtained from Drs. Mike Farzan and Huihui Mu at Scripps) as detailed in Shen et al. [2].

Briefly, spike-pseudotyped virus was prepared by transfection in 293T cells (human embryonic kidney cells in origin; obtained from American Type Culture Collection, cat. no. CRL-11268) using a lentivirus backbone vector, a spike-expression plasmid, a TMPRSS2 expression plasmid and a firefly Luc reporter plasmid. A pre-titrated dose of pseudovirus was incubated with eight serial 5-fold dilutions of serum samples (1:10 start dilution) in duplicate in 96-well flat-bottom poly-L-lysine-coated culture plates (Corning, cat. no. 354516) or 96-well, low evaporation, sterile, flat-bottom culture plates (Corning, cat. No. 3595) for 1 hr at 37°C prior to adding 293T/ACE2 cells. One set of eight wells received cells + virus (virus control) and another set of eight wells received cells only (background control), corresponding to technical replicates. Luminescence was measured after 66-72 hr of incubation using Promega 1X lysis buffer (Promega, cat. no. E1531) and Bright-Glo luciferase reagent (Promega, cat. no. E2650). Neutralization titers are the inhibitory dilution of serum samples at which relative luminescence units (RLUs) were reduced by either 50% (ID50) or 80% (ID80) compared to virus control wells after subtraction of background RLUs. Serum samples were heat-inactivated for 30 min at 56°C prior to assay.

***Viruses and cells***

VeroE6-TMPRSS2 cells were generated and cultured as previously described [3]. EHC-083E (B.1 lineage), contains a D614G mutation within the spike, was isolated from a residual nasopharyngeal swab from a patient in Atlanta, GA in March 2020 (SARS-CoV-2/human/USA/GA-EHC-083E/2020) [4]. We generated an infectious clone express eGFP based on the EHC-083E strain in a similar manner as previously described for nCoV/USA_WA1/2020 (WA/1) [5]. The spike gene of BA.1 and BA.5 were used to generate recombinant infectious clones-expressing eGFP. All recombinant viruses were propagated once in VeroE6-TMPRSS2 cells to generate working stocks. Viruses were deep sequenced and confirmed as previously described [6].

### **Focus Reduction Neutralization Test (FRNT)**

FRNT-egfp assays were performed in a similar manner as previously described [3, 6, 7]. Briefly, samples were diluted at 3-fold in 8 serial dilutions using DMEM in duplicates with an initial dilution of 1:10 in a total volume of 60 μl. Serially diluted samples were incubated with an equal volume of virus to achieve approximately 200 foci per well at 37^o^ C for 1 hour in a 96-well plate. The antibody-virus mixture was then added to VeroE6-TMPRSS2 cells and incubated at 37^o^C for 1 hour. Post-incubation, the antibody-virus mixture was removed, and 100 µl of pre-warmed 0.85% methylcellulose overlay was added to each well. Plates were incubated at 37^o^ C for 18 hours (D614G) to 40 hours (BA.1 and BA.5), and the methylcellulose overlay was removed and washed six times with PBS. Cells were fixed with 2% paraformaldehyde in PBS for 30 minutes. Cells were washed twice with 1x PBS and imaged on an ELISPOT reader (CTL Analyzer).

***Quantification and Statistical Analysis***

Antibody neutralization was quantified by counting the number of foci for each sample tested in duplicate using the Viridot program [8]. The average numbers of foci in each sample at the highest dilution were used to calculate the percent of neutralization as follows: 1 - (ratio of the mean number of foci in the presence of sera and foci at the highest dilution of the respective sera sample). The FRNT-egfp_50_ titers (the reciprocal dilution of sera samples that neutralizes 50% of the input virus) were interpolated using a 4-parameter nonlinear regression in GraphPad Prism 10.3.1. Samples that do not neutralize at the limit of detection at 50% are plotted at 20 and used for geometric mean and fold-change calculations.

# **Cellular Immunogenicity Assays**

***Peptides***

Custom-made, recombinant, lyophilized and overlapping (OLP) 15-mer peptides specific for Spike and TCE components were produced by Genscript (Piscataway, NJ, USA) and reconstituted at 1 or 2 mg/ml/peptide in sterile 20% (v/v) H_2_0 and 80% (v/v) DMSO (VWR International, Pittsburgh, PA, USA), aliquoted and stored at −80°C. Control peptides to assess responses to infectious disease antigens from CMV, EBV, Influenza (CEF peptide pool) were purchased from JPT Peptide Technologies (Berlin, Germany).

## **IFNγ Enzyme-linked immunosorbent assay (ELISpot) assays**

Detection of IFNγ-producing T cells was performed by ex vivo ELISpot assay. Briefly, cells were thawed, overnight rested, counted and re-suspended in media at 4 × 106 cells/ml and cultured in the presence of 20% (v/v) H_2_O and 80% (v/v) DMSO (VWR International), Phytohemagglutinin-L (PHA-L; Sigma-Aldrich, Natick, MA, USA), CEF peptide pool, or peptide pools in ELISpot Multiscreen plates (EMD Millipore) coated with anti-human IFNγ capture antibody (1:100 dilution, Mabtech, Cincinnati, OH, USA). Following 18-24 h incubation in a 5% CO_2_, 37 °C humidified incubator, supernatants were collected and membrane-bound IFNγ was detected using anti-human IFNγ detection antibody (1:500, Mabtech), Vectastain Avidin peroxidase complex (Vector Labs, Burlingame, CA, USA) and AEC Substrate (BD Biosciences, San Jose, CA, USA). ELISpot plates were allowed to dry and were imaged and enumerated on an AID iSpot plate reader (Autoimmun Diagnostika GmbH, Straßberg, Germany). Data are presented as spot forming units (SFU) per million cells. Total ex vivo Spike response is calculated by the sum of responses to OLP pools S 1-1, S 1-2, S 2-1, and S 2-2. Ex vivo ELISpot limit of detection (LOD) was determined at 30 SFU/106 PBMCs. T cell epitope (TCE) ELISpot responses are calculated by the sum of responses to OLP pools covering N, M and ORF3a regions of the TCE cassette. Responses below LOD were converted to ½*LOD. A participant was considered a responder if the baseline sample mean was < LOD and the post-vaccination test sample mean was ≥ 2-fold of LOD or the baseline sample mean was ≥ LOD and the post-vaccination test sample mean was ≥ 2-fold of baseline mean. The definition of a responder was for each individual peptide. Because the “Spike” outcome was the sum of 4 peptide values used, if any of the 4 peptide responses met the definition of a responder then the participant was considered as a “responder” for the overall “Spike” response. A similar rule was used for the other summed outcome of overlapping peptides covering N, M, and ORF3a regions of the TCE cassette. If any of the peptide responses met the definition of a “responder,” then the participant was considered a responder for the TCE peptides.

## **Meso Scale Discovery (MSD) U-plex assay**

Detection of secreted IL-2, TNF-α, IL-4, IL-10, and IL-13 in ex vivo ELISpot supernatants was performed in a subset of participants using a MSD U-PLEX Biomarker assay. Assays were performed according to the manufacturer’s instructions. Data were acquired on the MESO SECTOR S 600 instrument and DISCOVERY WORKBENCH assay analysis software. Analyte concentrations (pg ml−1) were calculated using serial dilutions of known standards for each cytokine. For graphical data representation, values below LLOQ were represented as LLOQ. LLOQ and upper limit of quantification (ULOQ) were defined as recommended by the manufacturer.

## **Intracellular Cytokine Staining (ICS) Assays**

Flow cytometry was used to examine SARS-CoV-2-specific CD4+ and CD8+ T-cell responses using a validated ICS assay. The assay was similar to a published report and the details of the staining panel are included in Supplementary Table 2 [9, 10]. Peptide pools covering the structural proteins of SARS-CoV2 were used for the six-hour stimulation. The spike peptide pools were 15 amino acids overlapping by 11 amino acids and were provided by BioSynthesis. All others were 13 amino acids overlapping by 11 amino acids and were provided by GenScript. The peptides covering the envelope (E), membrane (M) and nucleocapsid (N) were initially combined into one peptide pool, but the majority of the assays were performed using a separate pool for N and one that combined only E and M. Several of the open reading frame (ORF) peptides were combined into two pools, ORF 3a6 and ORF 7a7b8. All peptide pools were used at a final concentration of 1mg/ml for each peptide. As a negative control, cells were not stimulated, only the peptide diluent (DMSO) was included. As a positive control, cells were stimulated with a polyclonal stimulant, staphylococcal enterotoxin B (SEB). Cells expressing IFN-γ and/or IL-2 and/or CD154 was the primary immunogenicity endpoint for CD4 T cells and cells expressing IFN-γ and/or IL-2 and/or TNF-a was the primary immunogenicity endpoint for CD8 T cells. The overall response to SARS-CoV-2 was defined as the sum of the background-subtracted responses to each of the individual pools. The total number of CD4 T cells must have exceeded 10,000 and the total number of CD8 T cells must have exceeded 5,000 to determine for the assay data to be included in the analysis. To assess positivity for a peptide pool within a T cell subset, a two-by-two contingency table was constructed comparing the SARS-CoV-2 peptide stimulated and negative control data. The four entries in each table were the number of cells positive for the cytokine of interest such as IFN-γ and/or IL-2 and the number of cells negative for IFN-γ and IL-2, for both the stimulated and the negative control data. If both negative control replicates were included, then the average number of total cells and the average number of positive cells were used. A one-sided Fisher's exact test was applied to the table, testing whether the number of cytokine-producing cells for the stimulated data was equal to that for the negative control data. Since multiple individual tests (for each peptide pool) were conducted simultaneously, a multiplicity adjustment was made to the individual peptide pool p-values using the Bonferroni-Holm adjustment method. If the adjusted p-value for a peptide pool was ≤0.00001, the response to the peptide pool for the T cell subset was considered positive. Because the sample sizes (i.e., total cell counts for the T cell subset) were large, e.g., as high as 100,000 cells, the Fisher’s exact test has high power to reject the null hypothesis for very small differences. Therefore, the adjusted p-value significance threshold was chosen stringently (≤ 0.00001).

## **Supplementary Table 1. Study Enrollment for All Study Groups**

| **Stage** | **Study Population** | **Group** | **Sample Size** | **Vaccination(s)** | **Actual Number of Participants Enrolled** | **Interval Between Doses (Days)** |
| --- | --- | --- | --- | --- | --- | --- |
| 1 | Naïve  (18-60 yo) | 1 | 4 | 5 x 10^10^ vp ChAd-S (GRT-C907)/ 30 µg SAM-S (GRT-R908) | 4 | 28 |
|  |  | 3A | 3 | 30 µg SAM-S (GRT-R908)/ 30 µg SAM-S (GRT-R908) | 3 | 28 |
|  |  | 3B | 7 | 30 µg SAM-S (GRT-R908)/ 3 µg SAM-S (GRT-R908) | 7 | 84-129 |
|  |  | 4 | 3 | 10 µg SAM-S-TCE (GRT-R910)/ 3 µg SAM-S-TCE (GRT-R910) | 3 | 84-129 |
| 2 | SAM-S-TCE Boosts after EUA/licensed mRNA (Groups 5-7) and Ad26 (Group 8) COVID-19 Vaccines (18-60 yo) | 5 | 10 | 3 µg SAM-S-TCE (GRT-R910) | 10 | ≥112* |
|  |  | 6 | 10 | 6 µg SAM-S-TCE (GRT-R910) | 10 | ≥112 |
|  |  | 7 | 8-12 | 10 µg SAM-S-TCE (GRT-R910) | Did not enroll | ≥112 |
|  |  | 8 | 8-12 | 10 µg SAM-S-TCE (GRT-R910) /  10 µg SAM-S-TCE (GRT-R910) | Did not enroll | ≥112/56 |
|  | SAM-S-TCE Boosts after approved/licensed mRNA (Groups 9-11) and Ad26 (Group 12) COVID-19 Vaccines (>60 yo) | 9 | 8 | 3 µg SAM-S-TCE (GRT-R910) | 8 | ≥112 |
|  |  | 10 | 8-12 | 6 µg SAM-S-TCE (GRT-R910) | 10 | ≥112 |
|  |  | 11 | 8-12 | 10 µg SAM-S-TCE (GRT-R910) | 10 | ≥112 |
|  |  | 12 | 8-12 | 10 µg SAM-S-TCE (GRT-R910) /  10 µg SAM-S-TCE (GRT-R910) | Did not enroll | ≥112/56 |
|  | ChAd-S-TCE Boosts after approved/licensed mRNA COVID-19 Vaccines (>60 yo) | 13 | 7-10 | 5 x 10^10^ vp ChAd-S-TCE  (GRT-C909) | 7 | ≥112 |
|  |  | 14 | 7-10 | 1 x 10^11^ vp ChAd-S-TCE  (GRT-C909) | 7 | ≥112 |
|  |  | 15 | 7-10 | 5 x 10^11^ vp ChAd-S-TCE  (GRT-C909) | 2 | ≥112 |

*Interval for Stage 2 of ≥112 days is the time required between last COVID vaccination and study vaccine

## **Supplementary Table 2. Reagents for Intracellular Cytokine Staining Assays**

| **Detector** | **Fluorochrome** | **Specificity** | **Clone** | **Purpose** |
| --- | --- | --- | --- | --- |
| B515 | FITC | Perforin | B-D48 | Function |
| B610 | BB630 | IL-5 | TRFK5 | Function |
| B610 | BB630 | IL-13 | JES10-5A2 | Function |
| B660 | BB660 | Ki67 | B56 | Activation |
| B710 | BB700 | IL-4 | MP4-25D2 | Function |
| G575 | PE | CRTh2 | BM16 | T helper class |
| G610 | PE-Dazzle594 | CD32 | FUN-2 | Fcγ receptor |
| G660 | Pe-Cy5 | CXCR3 (CD183) | 1C6/CXCR3 | T helper class |
| G710 | PE-Cy5.5 | FOXP3 | PCH101 | Treg |
| G780 | PE-Cy7 | IL-17a | BL168 | Function |
| R660 | APC | IL-2 | MQ1-17H12 | Function |
| R710 | Alexa 700 | Granzyme B | GB11 | Function |
| R780 | APC-Fire750 | CD3 | UCHT1 | T cell lineage |
| U395 | BUV395 | TNF | MAb11 | Function |
| U450 | UViD | Viability | N/A | Viability |
| U500 | BUV496 | CD45RA | HI100 | Differentiation |
| U570 | BUV563 | CD19 | SJ25C1 | B cells |
| U660 | BUV661 | CD14 | MΦP9 | Monocytes |
| U730 | BUV737 | CD154 | TRAP1 | Function |
| U780 | BUV805 | CD8 | SK1 | T cell lineage |
| V450 | V450 | IFNγ | B27 | Function |
| V510 | BV480 | CD4 | SK3 | Function |
| V570 | BV570 | CD16 | 3G8 | NK, NKT |
| V610 | BV605 | CCR7 | G034H7 | Differentiation |
| V655 | BV650 | CD25 | M-A251 | Treg, activation |
| V710 | BV711 | CD64 | 10.1 | Fcγ receptor |
| V750 | BV750 | CD56 | 5.1H11 | NK, NKT |
| V780 | BV786 | CCR6 (CD196) | 11A9 | T helper class |

## **Supplementary Table 3. Numbers of participants who tested positive for Nucleocapsid (N)-antibody during the course of the study**

| **Planned Time Point** | **Statistic** | **Group 5: 3 µg GRT-R910 (N=10)** | **Group 6: 6 µg GRT-R910 (N=10)** | **Group 9: 3 µg GRT-R910 (N=8)** | **Group 10: 6 µg GRT-R910 (N=10)** | **Group 11: 10 µg GRT-R910 (N=10)** |
| --- | --- | --- | --- | --- | --- | --- |
| **Day 1** | **Number N-Antibody Positive** | 0 | 0 | 0 | 2 | 2 |
|  | **% Positive** | 0 | 0 | 0 | 20 | 20 |
| **Day 85** | **Number N-Antibody Positive** | 0 | 0 | 2 | 3 | 2 |
|  | **% Positive** | 0 | 0 | 25 | 30 | 20 |
| **Day 181** | **Number N-Antibody Positive** | 3 | 4 | 4 | 4 | 2 |
|  | **% Positive** | 30 | 40 | 50 | 40 | 20 |

## **Supplementary Table 4. New onset chronic medical conditions (NOCMC) occurring during the study**

| **NOCMC** | **Number of days after study vaccine** | **Severity** | **Relationship** | **Outcome** |
| --- | --- | --- | --- | --- |
| **Group 5: 3 µg GRT-R910 in persons aged 18-60 years** | | | | |
| Uterine fibroid | 27 | Mild | Not related | Not recovered/not resolved |
| Ovarian cyst | 27 | Mild | Not related | Not recovered/not resolved |
| **Group 6: 6 µg GRT-R910 in persons aged 18-60 years** | | | | |
| Bipolar disorder | 156 | Moderate | Not related | Recovering/resolving |
| Raynaud’s phenomenon | 90 | Mild | Not related | Not recovered/not resolved |
| Asthma | 268 | Mild | Not related | Recovered/resolved |
| **Group 9: 3 µg GRT-R910 in persons aged > 60 years** | | | | |
| Type 2 diabetes mellitus | 237 | Moderate | Not related | Recovered/resolved |
| **Group 10: 6 µg GRT-R910 in persons aged > 60 years** | | | | |
| Hyperlipidemia | 74 | Mild | Not related | Recovering/resolving |
| High cholesterol | 268 | Moderate | Not related | Recovering/resolving |

## **Supplementary Table 5. Summary of pseudovirus neutralizing antibody test ID_50_ responses against SARS-CoV-2 BA.1, per-protocol population**

##

| **Time Point** | **Statistic** | **Group 5:  3 µg GRT-R910 (N=10)** | **Group 6:  6 µg GRT-R910 (N=10)** | **Group 9:  3 µg GRT-R910 (N=8)** | **Group 10:  6 µg GRT-R910 (N=10)** | **Group 11:  10 µg GRT-R910 (N=10)** |
| --- | --- | --- | --- | --- | --- | --- |
| **Day 1 (Pre-Vaccination)** | n | 10 | 10 | 8 | 10 | 10 |
|  | GMT (95% CI) | 17.8 (10.5, 30.3) | 33.4 (14.6, 76.4) | 11.9 (7.2, 19.5) | 81.1 (15.5, 423.4) | 87.9 (46.8, 165.2) |
|  | Seropositive (95% CI) | 80 (44, 97) | 80 (44, 97) | 75 (35, 97) | 80 (44, 97) | 100 (69, 100) |
| **Day 15 Post Vaccination** | n | 10 | 9 | 8 | 10 | 9 |
|  | GMT (95% CI) | 85.7 (35.5, 206.7) | 113.8 (33.0, 392.6) | 77.1 (25.4, 233.9) | 141.3 (33.8, 590.7) | 150.2 (76.5, 294.6) |
|  | GMR_D614G_ (95% CI) | 0.2 (0.1, 0.4) | 0.1 (0.04, 0.3) | 0.1 (0.06, 0.2) | 0.2 (0.1, 0.3) | 0.2 (0.1, 0.3) |
|  | GMFR (95% CI) | 4.8 (1.8, 13.1) | 4.2 (1.3, 13.4) | 6.5 (2.0, 20.9) | 1.7 (0.8, 3.6) | 2.2 (1.2, 4.2) |
|  | Seropositive (95% CI) | 100 (69, 100) | 89 (52, >99) | 100 (63, 100) | 90 (55, >99) | 100 (66, 100) |
| **Day 29 Post Vaccination** | n | 9 | 10 | 8 | 9 | 10 |
|  | GMT (95% CI) | 173.4 (60.3, 499.1) | 238.4 (50.3, 1129.7) | 109.2 (40.5, 294.2) | 171.0 (39.9, 731.9) | 253.2 (136.9, 468.4) |
|  | GMR_D614G_ (95% CI) | 0.3 (0.2, 0.6) | 0.2 (0.06, 0.4) | 0.1 (0.08, 0.2) | 0.1 (0.1, 0.3) | 0.2 (0.12, 0.36) |
|  | GMFR (95% CI) | 10.2 (2.9, 35.3) | 7.1 (2.0, 25.8) | 9.2 (3.0, 28.0) | 2.5 (1.00, 6.2) | 2.9 (1.4, 6.1) |
|  | Seropositive (95% CI) | 100 (66, 100) | 90 (55, >99) | 100 (63, 100) | 100 (66, 100) | 100 (69, 100) |
| **Day 85 Post Vaccination** | n | 10 | 10 | 6 | 9 | 9 |
|  | GMT (95% CI) | 138.1 (52.1, 366.1) | 244.4 (47.9, 1246.8) | 129.1 (63.8, 261.3) | 490.3 (177.8, 1352.2) | 316.7 (172.5, 581.5) |
|  | GMR_D614G_ (95% CI) | 0.25 (0.1, 0.5) | 0.2 (0.1, 0.5) | 0.2 (0.1, 0.3) | 0.3 (0.1, 0.5) | 0.18 (0.1, 0.3) |
|  | GMFR (95% CI) | 7.8 (2.5, 24.5) | 7.3 (1.9, 28.8) | 11.0 (3.5, 35.0) | 4.4 (1.4, 14.1) | 3.3 (1.6, 7.1) |
|  | Seropositive (95% CI) | 100 (69, 100) | 90 (55, >99) | 100 (54, 100) | 100 (66, 100) | 100 (66, 100) |
| **Day 181 Post Vaccination** | n | 5 | 5 | 4 | 6 | 10 |
|  | GMT (95% CI) | 154.4 (64.4, 369.9) | 85.0 (17.7, 407.8) | 157.0 (40.6, 606.7) | 472.6 (118.9, 1877.9) | 294.2 (145.1, 596.7) |
|  | GMR_D614G_ (95% CI) | 0.2 (0.1, 0.3) | 0.2 (0.06, 0.5) | 0.3 (0.08, 1.0) | 0.3 (0.1, 0.6) | 0.2 (0.2, 0.4) |
|  | GMFR (95% CI) | 8.1 (1.6, 41.4) | 3.17 (0.2, 45.3) | 15.3 (1.8, 130.1) | 2.1 (0.6, 7.3) | 3.4 (1.5, 7.4) |
|  | Seropositive (95% CI) | 100 (48, 100) | 100 (48, 100) | 100 (40, 100) | 100 (54, 100) | 100 (69, 100) |
| **Day 366 Post Vaccination** | n | 2 | 1 | 2 | 1 | 6 |
|  | GMT (95% CI) | 2492.9 (1586.6, 3917.0) | 1643.6 (NE) | 71.2 (28.0, 181.2) | 26.1 (NE) | 541.9 (139.83, 2100.3) |
|  | GMR_D614G_ (95% CI) | 0.7 (0.2, 2.3) | 0.4 (NE) | 0.4 (0.01, 16.5) | 0.04 (NE) | 0.4 (0.2, 0.7) |
|  | GMFR (95% CI) | 75.2 (67.2, 84.2) | 44.6 (NE) | 7.5 (0.01, 10199.8) | 1.6 (NE) | 4.9 (0.9, 27.3) |
|  | Seropositive (95% CI) | 100 (16, 100) | 100 (3, 100) | 100 (16, 100) | 100 (3, 100) | 100 (54, 100) |
| Notes: N = Number of participants enrolled. n = Number of participants in the Per Protocol population with available results. NE = Not Estimable. GMT = Geometric Mean Titer, GMFR = Geometric Mean Fold Rise, GMR_D614G_ = Geometric Mean Ratio to D614G. | | | | | | |

## **Supplementary Table 6. Summary of pseudovirus neutralizing test ID_50_ responses against SARS-CoV-2 BA.4/5, per-protocol population**

| Time Point | Statistic | Group 5:  3 µg GRT-R910 (N=10) | Group 6:  6 µg GRT-R910 (N=10) | Group 9:  3 µg GRT-R910 (N=8) | Group 10:  6 µg GRT-R910 (N=10) | Group 11:  10 µg GRT-R910 (N=10) |
| --- | --- | --- | --- | --- | --- | --- |
| **Day 1 (Pre-Vaccination)** | n | 10 | 10 | 8 | 10 | 10 |
|  | GMT (95% CI) | 12.5 (7.3, 21.5) | 23.9 (11.2, 51.2) | 45.8 (18.6, 113.1) | 94.1 (24.5, 360.8) | 38.4 (15.6, 94.6) |
|  | Seropositive (95% CI) | 70 (35, 93) | 80 (44, 97) | 100 (63, 100) | 90 (55, >99) | 90 (55, >99) |
| **Day 15 Post Vaccination** | n | 10 | 9 | 8 | 10 | 9 |
|  | GMT (95% CI) | 54.1 (22.1, 132.1) | 59.4 (19.5, 181.2) | 154.9 (69.5, 345.2) | 94.8682 (31.8, 282.7) | 80.3 (35.1, 183.4) |
|  | GMR_D614G_ (95% CI) | 0.1 (0.1, 0.3) | 0.1 (0.0, 0.1) | 0.2 (0.1, 0.4) | 0.1 (0.1, 0.2) | 0.1 (0.1, 0.2) |
|  | GMFR (95% CI) | 4.3 (1.9, 9.9) | 3.0 (1.2, 7.8) | 3.4 (1.0, 11.1) | 1.0 (0.6, 1.7) | 2.8 (1.3, 6.0) |
|  | Seropositive (95% CI) | 90 (55, >99) | 89 (52, >99) | 100 (63, 100) | 90 (55, >99) | 100 (66, 100) |
| **Day 29 Post Vaccination** | n | 9 | 10 | 8 | 9 | 10 |
|  | GMT (95% CI) | 88.4 (30.2, 259.1) | 116.9 (28.5, 479.7) | 280.2 (103.5, 758.5) | 142.8 (40.9, 498.5) | 165.1 (80.5, 338.5) |
|  | GMR_D614G_ (95% CI) | 0.2 (0.1, 0.3) | 0.1 (0.04, 0.2) | 0.3 (0.1, 0.8) | 0.1 (0.1, 0.2) | 0.1 (0.1, 0.2) |
|  | GMFR (95% CI) | 7.7 (2.7, 22.1) | 4.9 (1.7, 14.5) | 6.1 (1.5, 25.6) | 1.5 (0.7, 3.3) | 4.3 (1.9, 9.8) |
|  | Seropositive (95% CI) | 89 (52, >99) | 90 (55, >99) | 100 (63, 100) | 89 (52, >99) | 100 (69, 100) |
| **Day 85 Post Vaccination** | n | 10 | 10 | 6 | 9 | 9 |
|  | GMT (95% CI) | 76.0 (29.2, 197.7) | 129.9 (32.8, 513.5) | 135.7 (43.4, 423.9) | 230.8 (90.9, 586.1) | 203.7 (84.4, 491.8) |
|  | GMR_D614G_ (95% CI) | 0.1 (0.1, 0.2) | 0.1 (0.1, 0.2) | 0.2 (0.1, 0.8) | 0.1 (0.06, 0.3) | 0.1 (0.1, 0.22) |
|  | GMFR (95% CI) | 6.1 (2.4, 15.5) | 5.4 (1.9, 15.5) | 2.0 (0.7, 5.5) | 1.8 (0.8, 4.2) | 4.9 (2.1, 11.4) |
|  | Seropositive (95% CI) | 90 (55, >99) | 90 (55, >99) | 100 (54, 100) | 100 (66, 100) | 100 (66, 100) |
| **Day 181 Post Vaccination** | n | 5 | 5 | 4 | 6 | 10 |
|  | GMT (95% CI) | 154.9 (63.7, 376.5) | 54.4 (13.7, 215.9) | 130.8 (8.6, 1978.6) | 244.5 (58.5, 1022.7) | 170.1 (67.3, 429.9) |
|  | GMR_D614G_ (95% CI) | 0.2 (0.1, 0.3) | 0.1 (0.04, 0.3) | 0.2 (0.02, 3.5) | 0.1 (0.04, 0.5) | 0.1 (0.1, 0.3) |
|  | GMFR (95% CI) | 9.7 (2.2, 43.7) | 3.2 (0.4, 29.9) | 1.4 (0.1, 15.1) | 1.3 (0.3, 6.0) | 4.4 (1.5, 13.0) |
|  | Seropositive (95% CI) | 100 (48, 100) | 100 (48, 100) | 100 (40, 100) | 100 (54, 100) | 100 (69, 100) |
| **Day 366 Post Vaccination** | n | 2 | 1 | 2 | 1 | 6 |
|  | GMT (95% CI) | 3013.8 (60.7, 149626.6) | 4147.0 (NE) | 246.7 (0.0, 66388624.4) | 35.6 (NE) | 538.4 (146.8, 1975.2) |
|  | GMR_D614G_ (95% CI) | 0.9 (0.1, 8.4) | 1.1 (NE) | 1.3 (0.0, 6047180.4) | 0.1 (NE) | 0.4 (0.2, 0.8) |
|  | GMFR (95% CI) | 152.4 (0.9, 25497.2) | 125.3 (NE) | 1.9 (0.0, 4366322.5) | 0.5 (NE) | 12.2 (2.2, 66.1) |
|  | Seropositive (95% CI) | 100 (16, 100) | 100 (3, 100) | 100 (16, 100) | 100 (3, 100) | 100 (54, 100) |
| Notes: N = Number of participants enrolled. n = Number of participants in the Per Protocol population with available results. NE = Not Estimable. GMT = Geometric Mean Titer, GMFR = Geometric Mean Fold Rise, GMR_D614G_ = Geometric Mean Ratio to D614G. | | | | | | |

## **Supplementary Table 7. Summary of focus reduction neutralization test ID_50_ against SARS-CoV-2 BA.1, per protocol population**

| **Time Point** | **Statistic** | **Group 5:  3 µg GRT-R910 (N=10)** | **Group 6:  6 µg GRT-R910 (N=10)** | **Group 9:  3 µg GRT-R910 (N=8)** | **Group 10:  6 µg GRT-R910 (N=10)** | **Group 11:  10 µg GRT-R910 (N=10)** |
| --- | --- | --- | --- | --- | --- | --- |
| **Day 1 (Pre-Vaccination)** | n | 10 | 10 | 8 | 10 | 10 |
|  | GMT (95% CI) | 10.0 (NE) | 16.7 (10.2, 27.3) | 10.0 (NE) | 39.7 (11.8, 133.9) | 29.5 (13.8, 63.4) |
|  | Seropositive (95% CI) | 0 (0, 31) | 40 (12, 74) | 0 (0, 37) | 50 (19, 81) | 60 (26, 88) |
| **Day 15 Post Vaccination** | n | 10 | 9 | 8 | 10 | 9 |
|  | GMT (95% CI) | 23.3 (12.2, 44.4) | 31.9 (13.9, 72.8) | 32.5 (12.1, 87.3) | 65.8 (15.4, 280.1) | 50.8 (21.8, 118.4) |
|  | GMR_D614G_ (95% CI) | 0.1 (0.1, 0.1) | 0.1 (0.0, 0.1) | 0.1 (0.0, 0.1) | 0.1 (0.01, 0.3) | 0.1 (0.0, 0.1) |
|  | GMFR (95% CI) | 2.3 (1.2, 4.4) | 2.1 (0.7, 6.2) | 3.3 (1.2, 8.7) | 1.7 (0.8, 3.3) | 2.1 (0.9, 5.0) |
|  | Seropositive (95% CI) | 50 (19, 81) | 67 (30, 93) | 75 (35, 97) | 60 (26, 88) | 78 (40, 97) |
| **Day 29 Post Vaccination** | n | 9 | 10 | 8 | 9 | 10 |
|  | GMT (95% CI) | 48.8 (17.3, 137.5) | 57.8 (23.3, 143.4) | 38.5 (15.2, 97.1) | 61.1 (20.2, 185.0) | 95.7 (41.8, 219.3) |
|  | GMR_D614G_ (95% CI) | 0.1 (0.1, 0.2) | 0.1 (0.1, 0.1) | 0.1 (0.0, 0.1) | 0.1 (0.1, 0.2) | 0.1 (0.0, 0.1) |
|  | GMFR (95% CI) | 4.9 (1.7, 13.8) | 3.5 (1.2, 9.7) | 3.9 (1.5, 9.7) | 1.6 (0.9, 3.2) | 3.2 (1.4, 7.5) |
|  | Seropositive (95% CI) | 67 (30, 93) | 80 (44, 97) | 75 (35, 97) | 78 (40, 97) | 90 (55, >99) |
| **Day 85 Post Vaccination** | n | 10 | 10 | 6 | 9 | 9 |
|  | GMT (95% CI) | 39.0 (15.1, 101.0) | 64.0 (23.7, 172.9) | 52.9 (19.7, 142.2) | 146.2 (26.3, 812.3) | 92.8 (45.1, 190.6) |
|  | GMR_D614G_ (95% CI) | 0.1 (0.1, 0.2) | 0.1 (0.1, 0.1) | 0.1 (0.0, 0.1) | 0.2 (0.0, 0.7) | 0.1 (0.1, 0.1) |
|  | GMFR (95% CI) | 3.9 (1.5, 10.1) | 3.8 (1.3, 11.3) | 5.3 (2.0, 14.2) | 3.2 (1.1, 8.9) | 2.8 (1.5, 5.2) |
|  | Seropositive (95% CI) | 60 (26, 88) | 80 (44, 97) | 83 (36, >99) | 89 (52, >99) | 89 (52, >99) |
| **Day 181 Post Vaccination** | n | 5 | 5 | 4 | 6 | 10 |
|  | GMT (95% CI) | 71.8 (21.9, 235.3) | 39.1 (10.8, 142.3) | 50.8 (23.3, 110.9) | 115.3 (23.6, 562.3) | 65.9 (30.6, 142.2) |
|  | GMR_D614G_ (95% CI) | 0.1 (0.1, 0.2) | 0.1 (0.1, 0.3) | 0.1 (0.0, 0.2) | 0.1 (0.0, 0.4) | 0.06 (0.0, 0.1) |
|  | GMFR (95% CI) | 7.2 (2.2, 23.5) | 1.9 (0.3, 14.6) | 5.1 (2.3, 11.1) | 1.5 (0.6, 3.8) | 2.23 (1.0, 4.8) |
|  | Seropositive (95% CI) | 80 (28, >99) | 60 (15, 95) | 100 (40, 100) | 83 (36, >99) | 90 (55, >99) |
| **Day 366 Post Vaccination** | n | 2 | 1 | 2 | 1 | 6 |
|  | GMT (95% CI) | 866.1 (0.1, 12610631.4) | 535.2350 (NE) | 39.3 (1.6, 979.8) | 10.0 (NE) | 134.3 (20.8, 869.7) |
|  | GMR_D614G_ (95% CI) | 0.4 (0.29, 0.51) | 0.20 (NE) | 0.1 (0.02, 0.8) | 0.01 (NE) | 0.1 (0.0, 0.3) |
|  | GMFR (95% CI) | 86.6 (0.01, 1261063.14) | 13.22 (NE) | 3.9 (0.16, 97.98) | 1.0 (NE) | 4.1 (0.3, 48.6) |
|  | Seropositive (95% CI) | 100 (16, 100) | 100 (3, 100) | 100 (16, 100) | 0 (0, 98) | 83 (36, >99) |
| Notes: N = Number of participants enrolled. n = Number of participants in the Per Protocol population with available results. NE = Not Estimable. GMT = Geometric Mean Titer, GMFR = Geometric Mean Fold Rise, GMR_D614G_ = Geometric Mean Ratio to D614G. | | | | |  |  |

## **Supplementary Table 8. Summary of focus reduction neutralization test ID_50_ against SARS-CoV-2 BA.4/5, per protocol population**

| **Time Point** | **Statistic** | **Group 5:  3 µg GRT-R910 (N=10)** | **Group 6:  6 µg GRT-R910 (N=10)** | **Group 9:  3 µg GRT-R910 (N=8)** | **Group 10:  6 µg GRT-R910 (N=10)** | **Group 11:  10 µg GRT-R910 (N=10)** |
| --- | --- | --- | --- | --- | --- | --- |
| **Day 1 (Pre-Vaccination)** | n | 10 | 10 | 8 | 10 | 10 |
|  | GMT (95% CI) | 10.0 (NE) | 13.7 (8.4, 22.4) | 11.3 (8.5, 14.8) | 29.4 (11.4, 75.7) | 16.5 (8.3, 32.9) |
|  | Seropositive (95% CI) | 0 (0, 31) | 20 (3, 56) | 13 (<1, 53) | 50 (19, 81) | 20 (3, 56) |
| **Day 15 Post Vaccination** | n | 10 | 9 | 8 | 10 | 9 |
|  | GMT (95% CI) | 21.8 (12.1, 39.3) | 23.9 (10.6, 53.8) | 33.3 (13.4, 83.3) | 35.3 (13.7, 91.14) | 28.9 (14.5, 57.4) |
|  | GMR_D614G_ (95% CI) | 0.1 (0.1, 0.1) | 0.1 (0.0, 0.1) | 0.1 (0.0, 0.1) | 0.1 (0.0, 0.1) | 0.0 (0.0, 0.1) |
|  | GMFR (95% CI) | 2.2 (1.2, 3.9) | 2.1(0.8, 5.4) | 3.0 (1.0, 8.6) | 1.2 (0.9, 1.7) | 2.3 (1.2, 4.4) |
|  | Seropositive (95% CI) | 50 (19, 81) | 44 (14, 79) | 63 (24, 91) | 60 (26, 88) | 67 (30, 93) |
| **Day 29 Post Vaccination** | n | 9 | 10 | 8 | 9 | 10 |
|  | GMT (95% CI) | 40.4 (15.2, 107.1) | 41.6 (16.9, 102.7) | 44.8 (16.6, 121.1) | 42.4 (13.9, 129.2) | 42.6 (21.5, 84.1) |
|  | GMR_D614G_ (95% CI) | 0.1 (0.1, 0.2) | 0.1 (0.0, 0.1) | 0.1 (0.0, 0.1) | 0.1 (0.0, 0.1) | 0.0 (0.0, 0.0) |
|  | GMFR (95% CI) | 4.0 (1.5, 10.7) | 3.0 (1.4, 6.8) | 4.0 (1.3, 12.7) | 1.5 (1.0, 2.3) | 2.6 (1.4, 4.8) |
|  | Seropositive (95% CI) | 67 (30, 93) | 70 (35, 93) | 75 (35, 97) | 67 (30, 93) | 80 (44, 97) |
| **Day 85 Post Vaccination** | n | 10 | 10 | 6 | 9 | 9 |
|  | GMT (95% CI) | 33.6 (13.8, 81.5) | 42.0 (17.5, 101.0) | 53.5 (19.7, 145.0) | 47.1 (18.2, 122.0) | 46.4 (21.3, 101.3) |
|  | GMR_D614G_ (95% CI) | 0.1 (0.0, 0.1) | 0.1 (0.0, 0.1) | 0.1 (0.0, 0.1) | 0.1 (0.0, 0.1) | 0.04 (0.0, 0.1) |
|  | GMFR (95% CI) | 3.4 (1.4, 8.2) | 3.1 (1.4, 6.6) | 4.6 (1.4, 14.9) | 1.4 (0.9, 2.2) | 2.7 (1.2, 5.9) |
|  | Seropositive (95% CI) | 60 (26, 88) | 70 (35, 93) | 83 (36, >99) | 78 (40, 97) | 78 (40, 97) |
| **Day 181 Post Vaccination** | n | 5 | 5 | 4 | 6 | 10 |
|  | GMT (95% CI) | 35.9 (18.6, 69.2) | 21.0 (5.7, 77.1) | 43.7 (7.3, 261.9) | 61.7 (12.3, 309.6) | 45.8 (19.5, 107.6) |
|  | GMR_D614G_ (95% CI) | 0.1 (0.0, 0.1) | 0.1 (0.0, 0.2) | 0.1 (0.0, 0.3) | 0.1 (0.0, 0.2) | 0.0 (0.0, 0.1) |
|  | GMFR (95% CI) | 3.6 (1.9, 6.9) | 1.7 (0.3, 8.9) | 4.4 (0.7, 26.2) | 1.2 (0.6, 2.3) | 2.8 (1.1, 7.2) |
|  | Seropositive (95% CI) | 80 (28, >99) | 40 (5, 85) | 75 (19, >99) | 83 (36, >99) | 70 (35, 93) |
| **Day 366 Post Vaccination** | n | 2 | 1 | 2 | 1 | 6 |
|  | GMT (95% CI) | 409.3 (5.0, 33644.3) | 553.8 (NE) | 36.5 (0.0, 1119461.2) | 10.0 (NE) | 88.1 (17.9, 434.1) |
|  | GMR_D614G_ (95% CI) | 0.2 (0.00, 24.3) | 0.2 (NE) | 0.1 (0.0, 14219.7) | 0.0 (NE) | 0.1 (0.0, 0.1) |
|  | GMFR (95% CI) | 40.9 (0.5, 3364.4) | 55.4 (NE) | 3.7 (0.0, 111946.1) | 1.0 (NE) | 5.6 (0.8, 38.9) |
|  | Seropositive (95% CI) | 100 (16, 100) | 100 (3, 100) | 50 (1, 99) | 0 (0, 98) | 83 (36, >99) |
| Notes: N = Number of participants enrolled. n = Number of participants in the Per Protocol population with available results. NE = Not Estimable. GMT = Geometric Mean Titer, GMFR = Geometric Mean Fold Rise, GMR_D614G_ = Geometric Mean Ratio to D614G. | | | | |  |  |

## **Supplementary Table 9. Summary of ELISA IgG antibody responses against SARS-CoV-2 S-2P, per-protocol population**

| Planned Time Point | Statistic | Group 5:  3 µg GRT-R910 (N=10) | Group 6:  6 µg GRT-R910 (N=10) | Group 9:  3 µg GRT-R190 (N=8) | Group 10:  6 µg GRT-R910 (N=10) | Group 11:  10 µg GRT-R910 (N=10) |
| --- | --- | --- | --- | --- | --- | --- |
| **Day 1 (Pre-Vaccination)** | n | 10 | 10 | 8 | 10 | 10 |
|  | GMT | 5.3 | 5.7 | 4.6 | 5.2 | 5.2 |
|  | 95% CI | 5.0, 5.6 | 5.2, 6.2 | 4.2, 5.0 | 4.7, 5.8 | 4.8, 5.6 |
| **Day 15 Post Vaccination** | n | 10 | 9 | 8 | 10 | 9 |
|  | GMT | 5.7 | 6.0 | 5.0 | 5.3 | 5.5 |
|  | 95% CI | 5.5, 6.0 | 5.6, 6.4 | 4.6, 5.5 | 4.8, 5.8 | 5.1, 5.9 |
| **Day 29 Post Vaccination** | n | 9 | 10 | 8 | 9 | 10 |
|  | GMT | 6.0 | 6.1 | 5.2 | 5.6 | 5.7 |
|  | 95% CI | 5.6, 6.4 | 5.7, 6.5 | 4.7, 5.7 | 5.1, 6.2 | 5.4, 5.9 |
| **Day 85 Post Vaccination** | n | 10 | 10 | 6 | 9 | 9 |
|  | GMT | 5.8 | 6.0 | 4.6 | 5.1 | 5.8 |
|  | 95% CI | 5.5, 6.1 | 5.6, 6.6 | 4.4, 4.9 | 4.8, 5.4 | 5.4, 6.2 |
| **Day 181 Post Vaccination** | n | 5 | 5 | 4 | 6 | 10 |
|  | GMT | 4.9 | 4.8 | 4.4 | 5.0 | 5.5 |
|  | 95% CI | 4.6, 5.3 | 4.5, 5.2 | 4.1, 4.7 | 4.7, 5.4 | 5.1, 5.9 |
| **Day 366 Post Vaccination** | n | 2 | 1 | 2 | 1 | 6 |
|  | GMT | 5.3 | 5.4 | 4.3 | 4.7 | 5.2 |
|  | 95% CI | 3.6, 7.8 | NE | 3.3, 5.5 | NE | 4.8, 5.7 |
| Notes: N = Number of participants enrolled. n = Number of participants in the Per Protocol population with available results. NE = Not Estimable. GMT = Geometric Mean Titer. | | | | | | |

## **Supplementary Table 10: Summary of ELISA IgG antibody responses against SARS-CoV-2 RBD, per-protocol population**

| **Planned Time Point** | **Statistic** | **Group 5:   3 µg GRT-R910  (N=10)** | **Group 6:   6 µg GRT-R910  (N=10)** | **Group 9:   3 µg GRT-R190  (N=8)** | **Group 10:   6 µg GRT-R910  (N=10)** | **Group 11:   10 µg GRT-R910  (N=10)** |
| --- | --- | --- | --- | --- | --- | --- |
| **Day 1**  **(Pre-Vaccination)** | n | 10 | 10 | 8 | 10 | 10 |
|  | GMT | 5.6 | 6.1 | 4.9 | 5.3 | 5.1 |
|  | 95% CI | 5.2, 6.0 | 5.7, 6.6 | 4.4, 5.4 | 4.6, 6.0 | 4.6, 5.7 |
| **Day 15 Post Vaccination** | n | 10 | 9 | 8 | 10 | 9 |
|  | GMT | 6.1 | 6.3 | 5.4 | 5.5 | 5.2 |
|  | 95% CI | 5.9, 6.3 | 6.0, 6.7 | 4.9, 5.9 | 5.0, 6.1 | 4.7, 5.8 |
| **Day 29 Post Vaccination** | n | 9 | 10 | 8 | 9 | 10 |
|  | GMT | 6.1 | 6.3 | 5.5 | 5.1 | 5.42 |
|  | 95% CI | 5.7, 6.6 | 5.9, 6.6 | 5.1, 6.0 | 4.8, 5.5 | 5.2, 5.7 |
| **Day 85 Post Vaccination** | n | 10 | 10 | 6 | 9 | 9 |
|  | GMT | 5.9 | 6.3 | 5.3 | 5.3 | 5.6 |
|  | 95% CI | 5.6, 6.3 | 5.8, 6.7 | 4.9, 5.7 | 4.9, 5.8 | 5.1, 6.1 |
| **Day 181 Post Vaccination** | n | 5 | 5 | 4 | 6 | 10 |
|  | GMT | 5.6 | 5.9 | 5.0 | 5.3 | 5.5 |
|  | 95% CI | 5.3, 6.0 | 5.2, 6.6 | 4.3, 5.7 | 5.0, 5.7 | 5.0, 6.0 |
| **Day 366 Post Vaccination** | n | 2 | 1 | 2 | 1 | 6 |
|  | GMT | 5.6 | 5.7 | 5.1 | 5.1 | 5.9 |
|  | 95% CI | 4.3, 7.3 | NE | 4.9, 5.2 | NE | 5.2, 6.7 |
| *Notes: N = Number of participants enrolled.*  *n = Number of participants in the Per Protocol population with available results.*  *NE = Not Estimable.*  *GMT = Geometric Mean Titer.* | | | | | | |

# **Supplemental Figures**

## **Supplemental Figure 1: Clinical Laboratory Results by Maximum Severity Post Baseline, 18‑60 years of age**


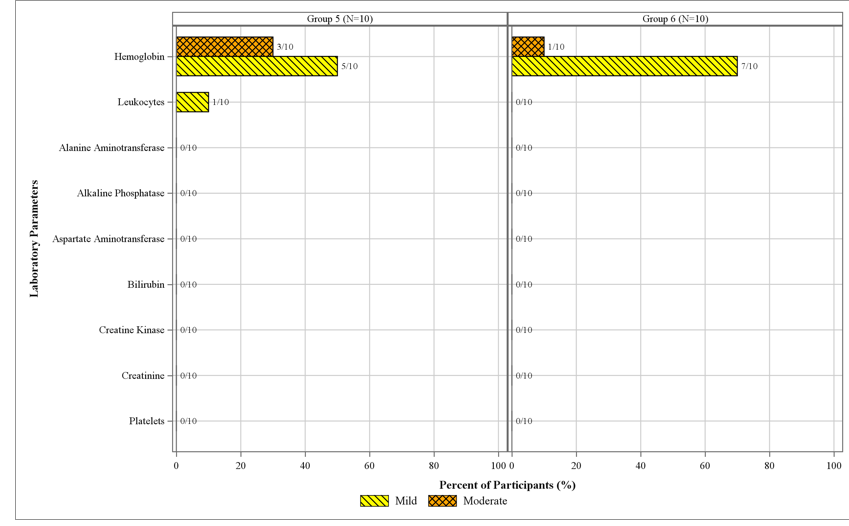


## **Supplemental Figure 2: Clinical Laboratory Results by Maximum Severity Post Baseline, >60 years of age**


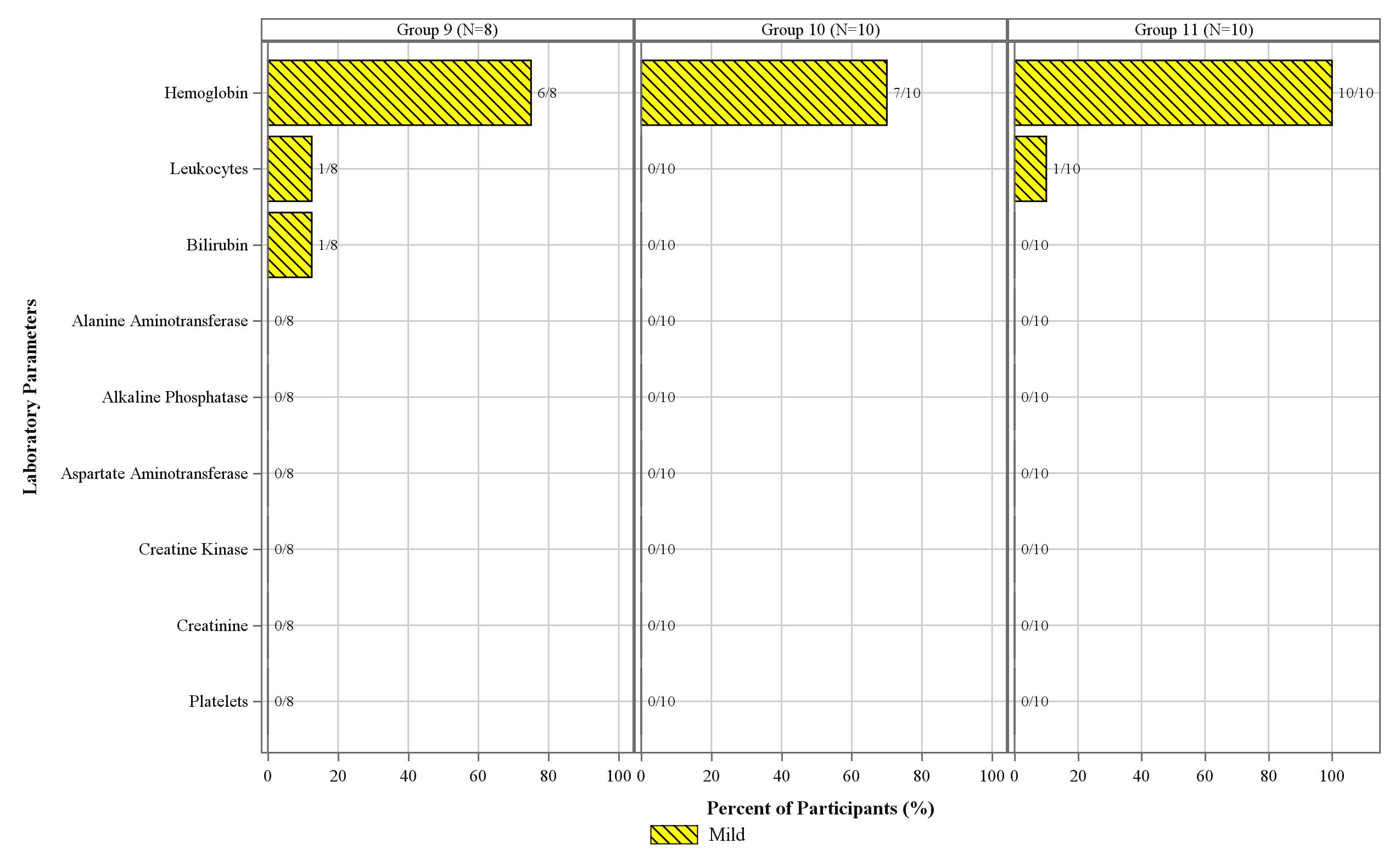


**Supplementary Figure 3. Focus reduction neutralization test ID50 against SARS-CoV-2 D614G, 18-60 years of age, per-protocol population**
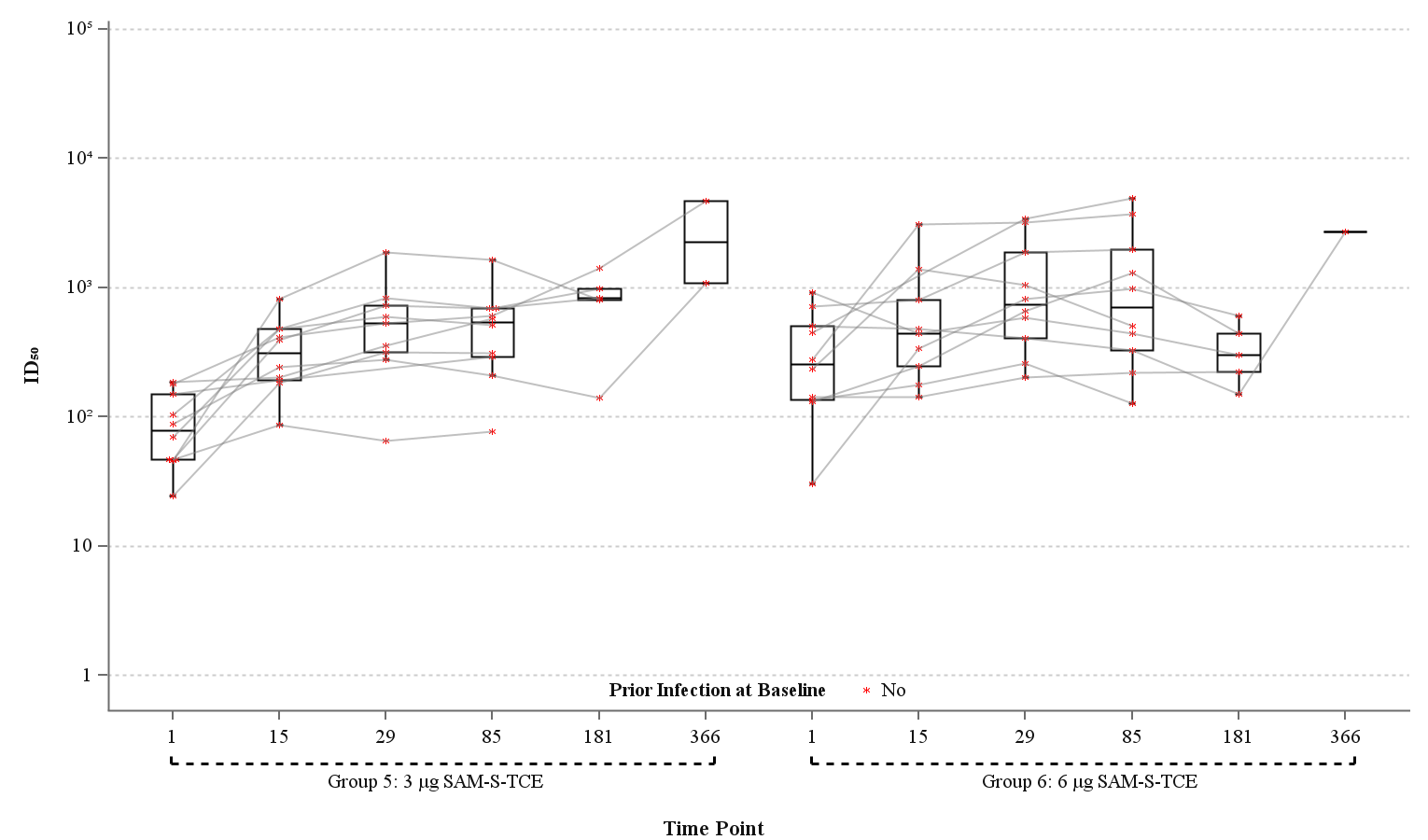


## **Supplementary Figure 4. Focus reduction neutralization test ID_50_ against SARS-CoV-2 D614G, >60 years of age, per-protocol population**


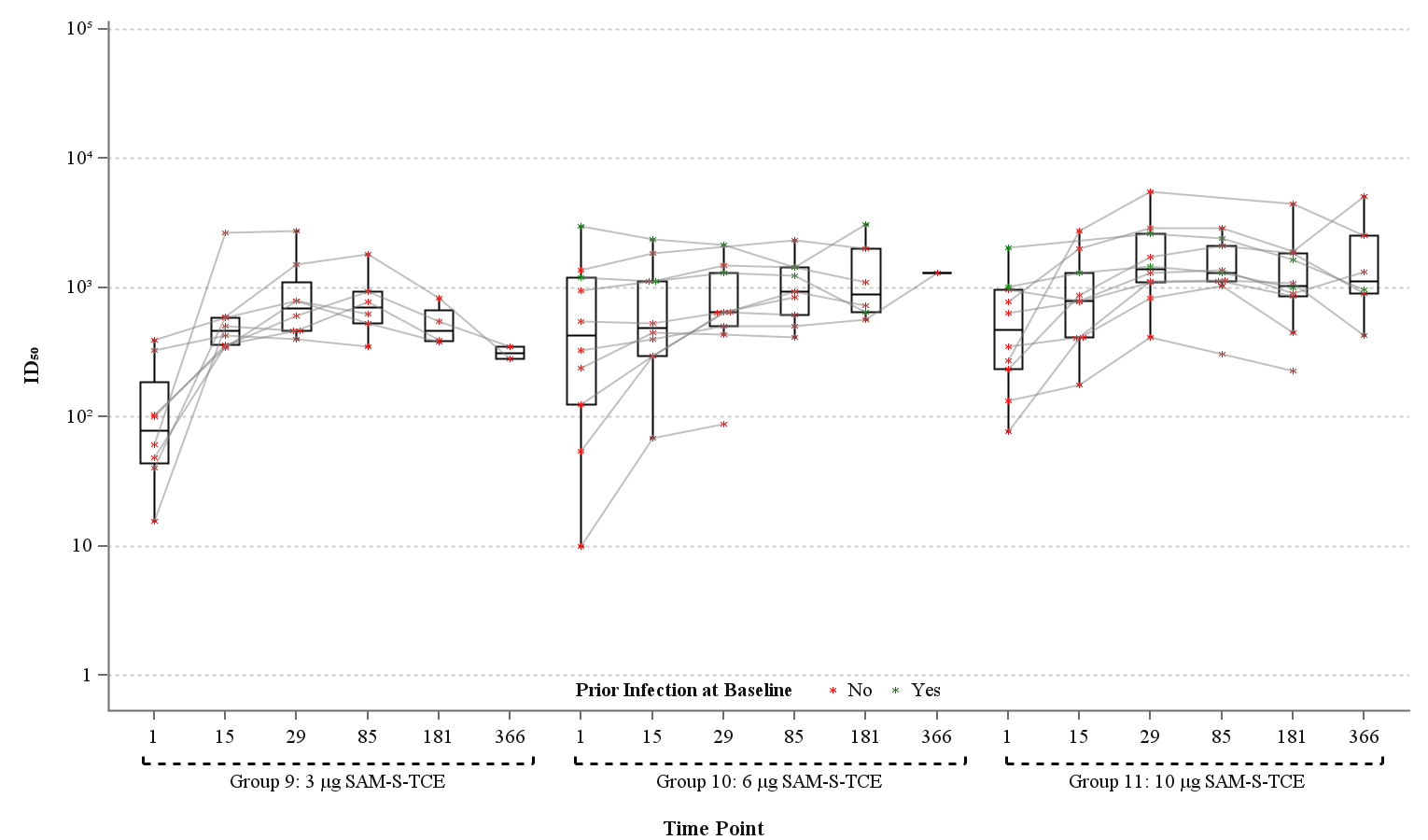


## **Supplemental Figure 5. Distribution of ELISA IgG Antibody Against SARS-CoV-2 S-2P, 18-60 years of age, per-protocol population**


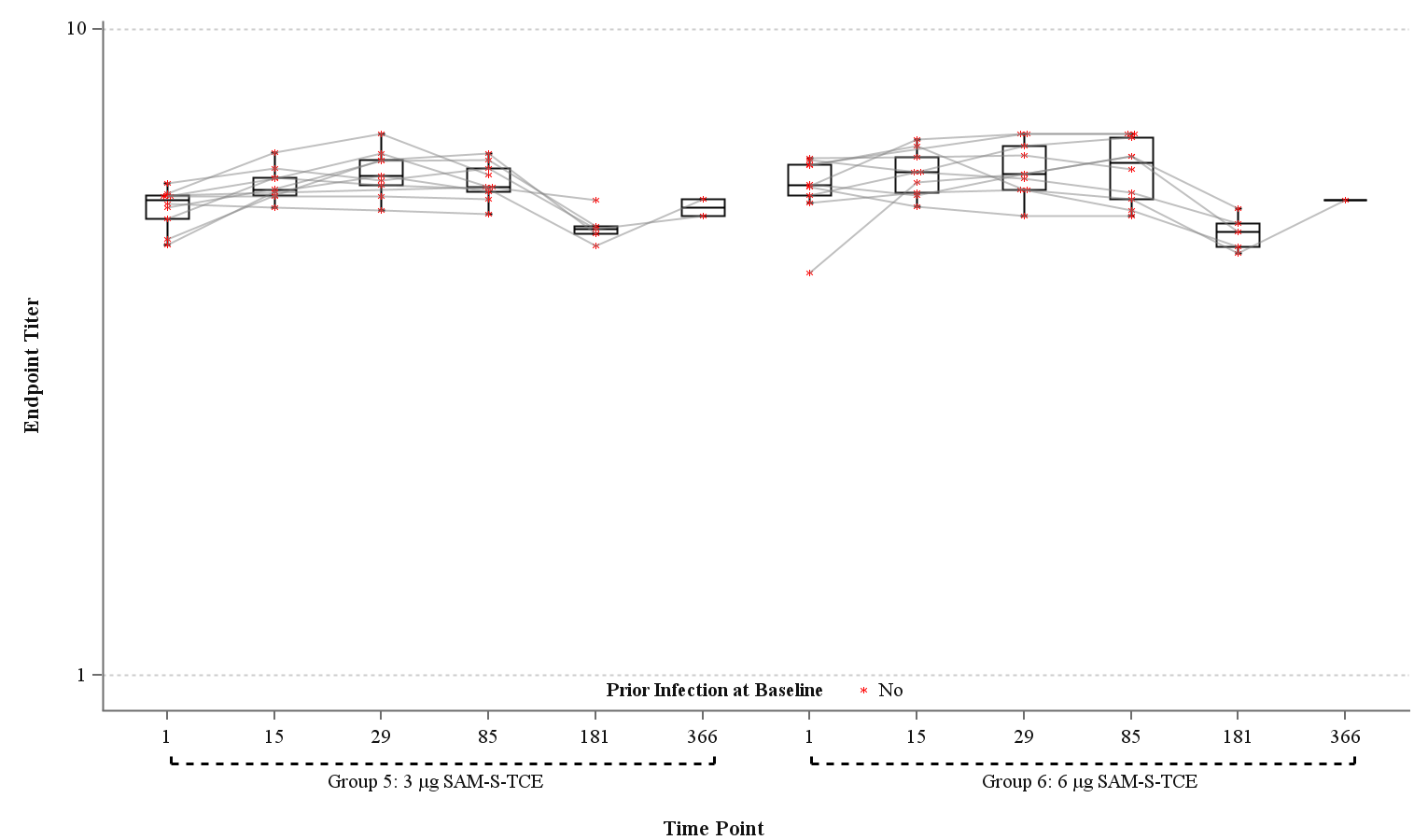


## **Supplemental Figure 6. Distribution of ELISA IgG antibody against SARS-CoV-2 S-2P, >60 years of age, per-protocol population**


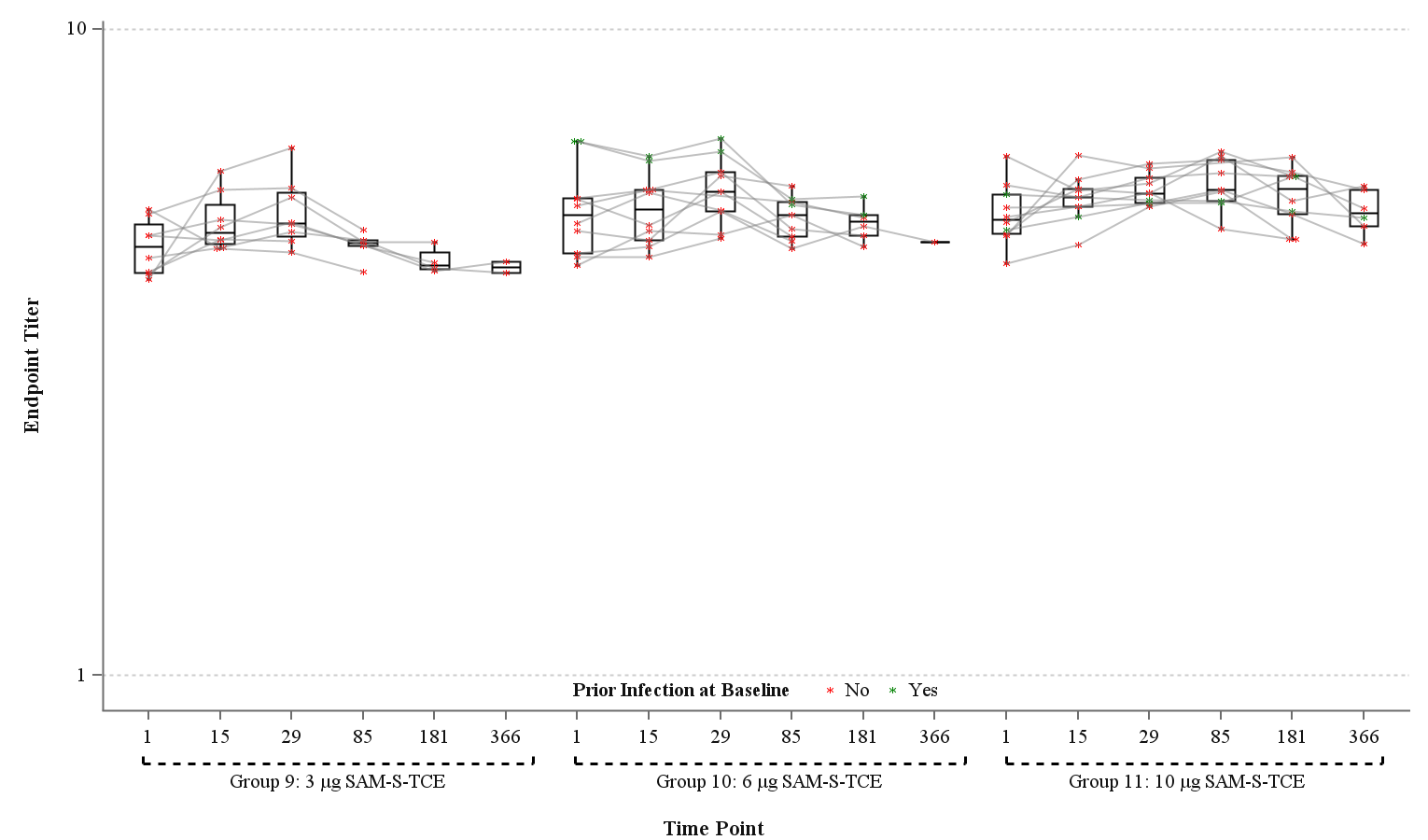


## **Supplemental Figure 7. Distribution of ELISA IgG antibody against SARS-CoV-2 RBD, 18-60 years of age, per-protocol population**


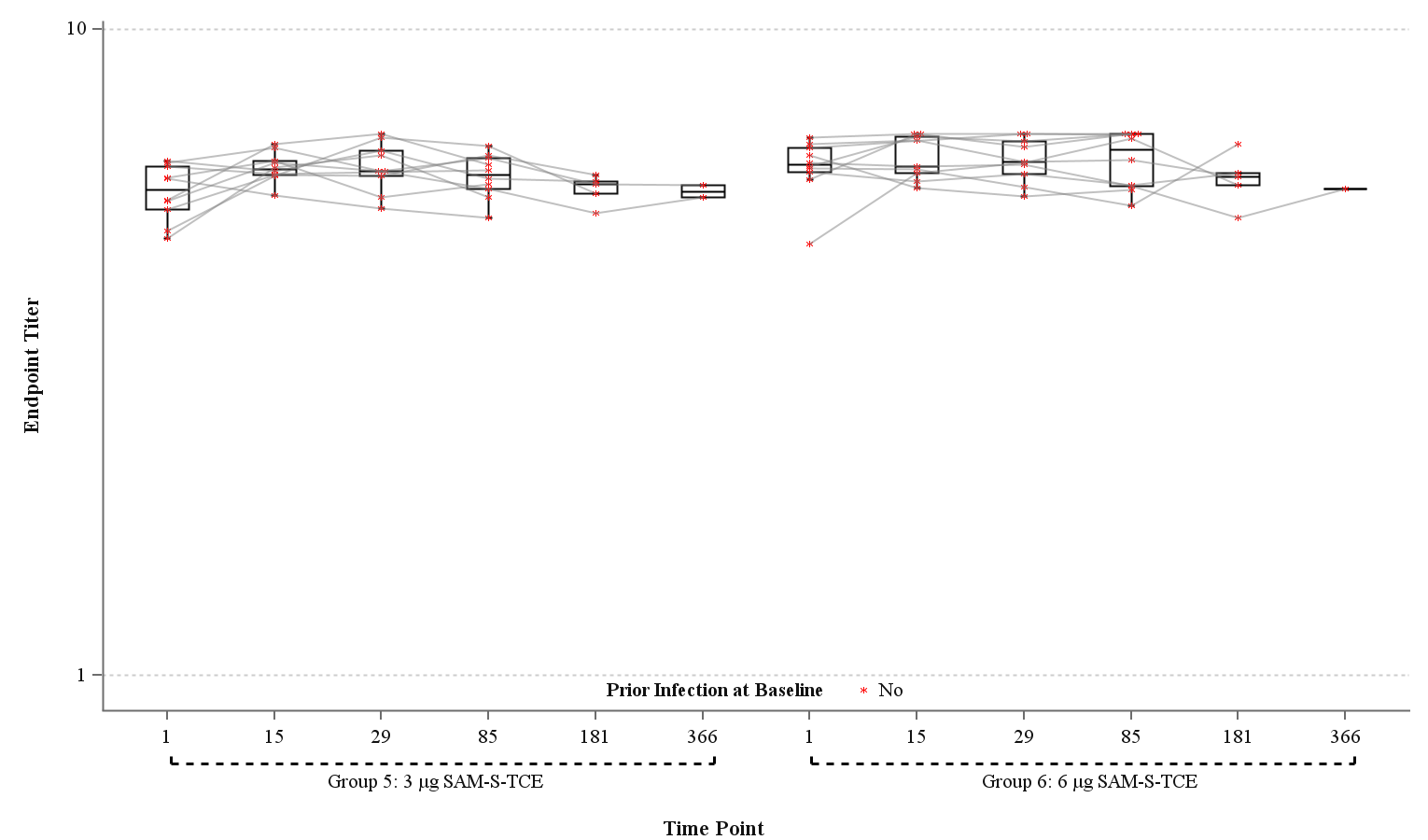


## **Supplemental Figure 8. Distribution of ELISA IgG antibody against SARS-CoV-2 RBD, >60 years of age, per-protocol population**


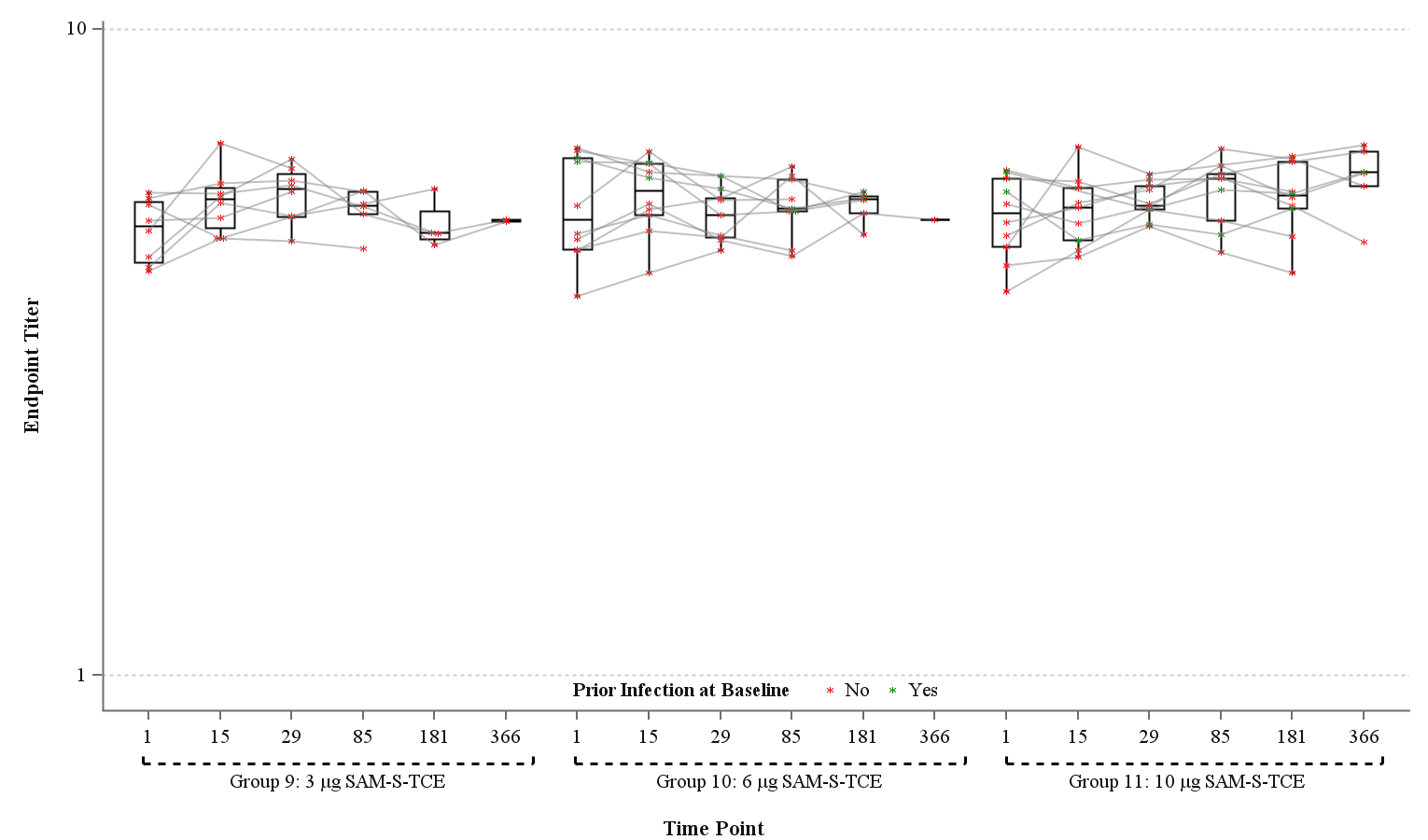


## **Supplementary Figure 9. Distribution of T Cells expressing IFNγ as measured by the ELISpot assay in spot forming units (SFU) per million, stimulated with Spike, 18-60 years of age, per-protocol population**


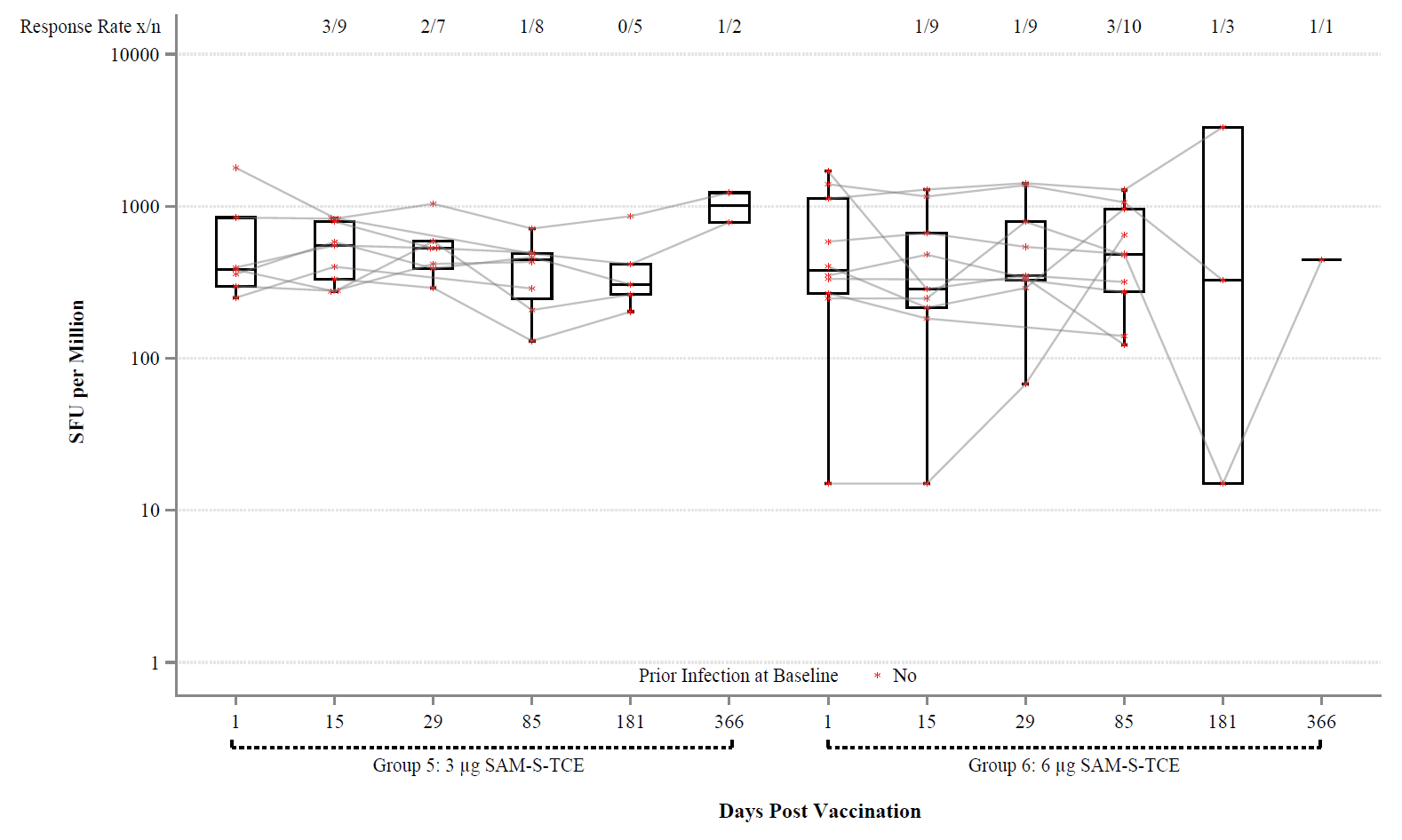


## **Supplementary Figure 10. Distribution of T Cells expressing IFNγ as measured by the ELISpot assay in spot forming units (SFU) per million, stimulated with Spike, >60 years of age, per-protocol population**


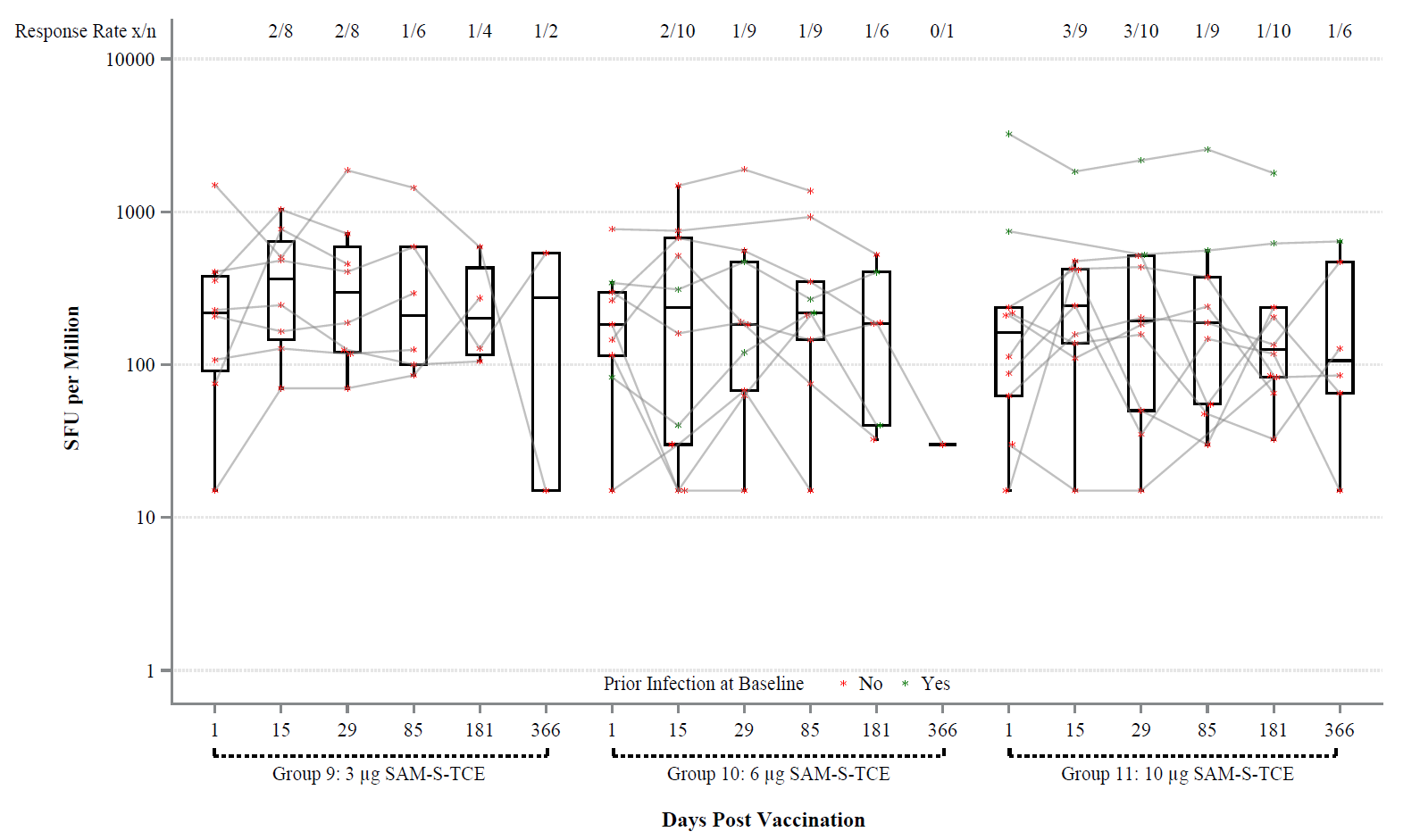


## **Supplementary Figure 11. Distribution of T Cells expressing IFNγ as measured by the ELISpot assay in spot forming units (SFU) per million, stimulation with 15mer OLP (overlapping peptides) spanning membrane, nucleocapsid, and open reading frame, 18-60 years of age, per-protocol population**


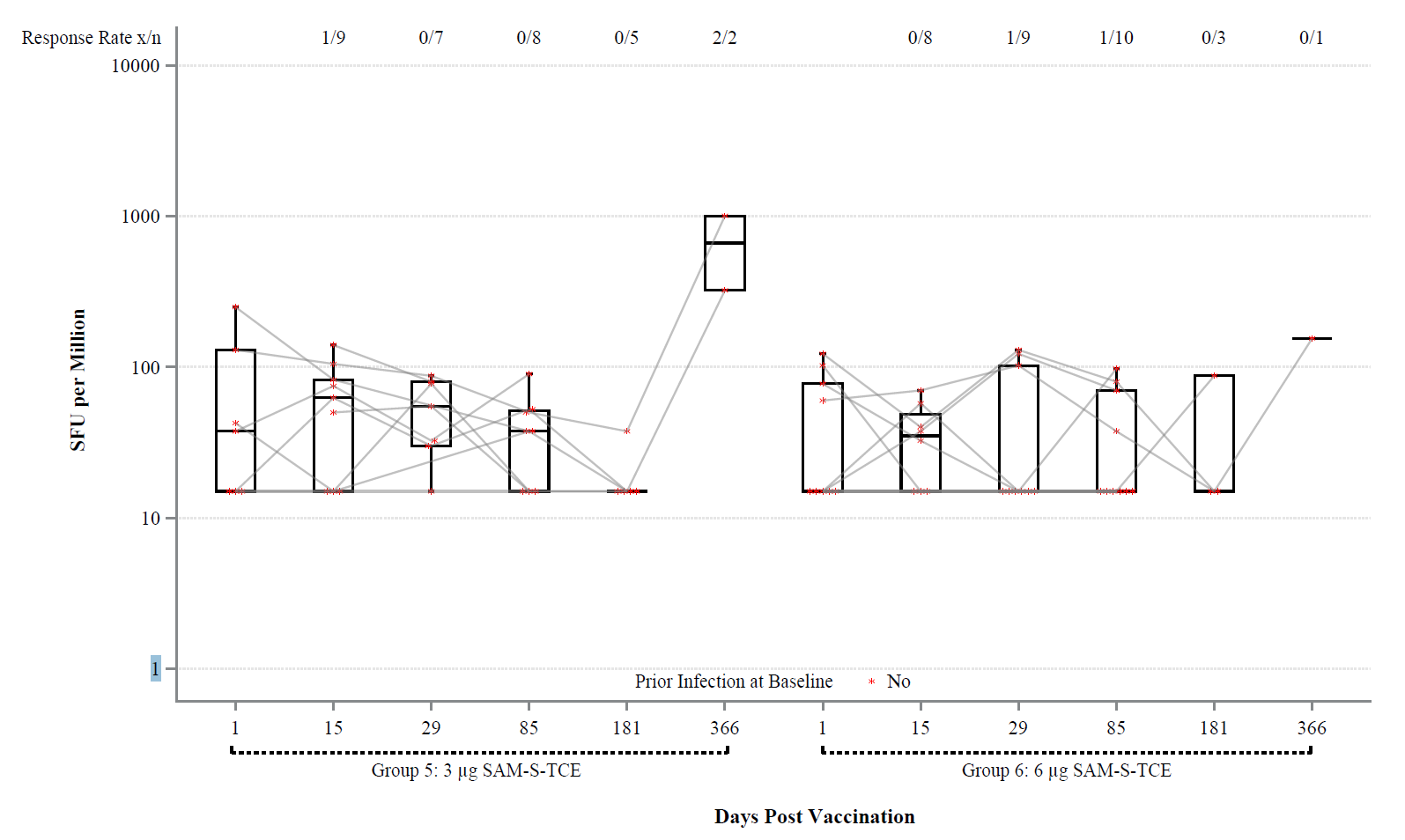


## **Supplementary Figure 12. Distribution of T Cells expressing IFNγ as measured by the ELISpot assay in spot forming units (SFU) per million, stimulation with 15mer OLP (overlapping peptides) spanning membrane, nucleocapsid, and open reading frame, >60 years of age, per-protocol population**


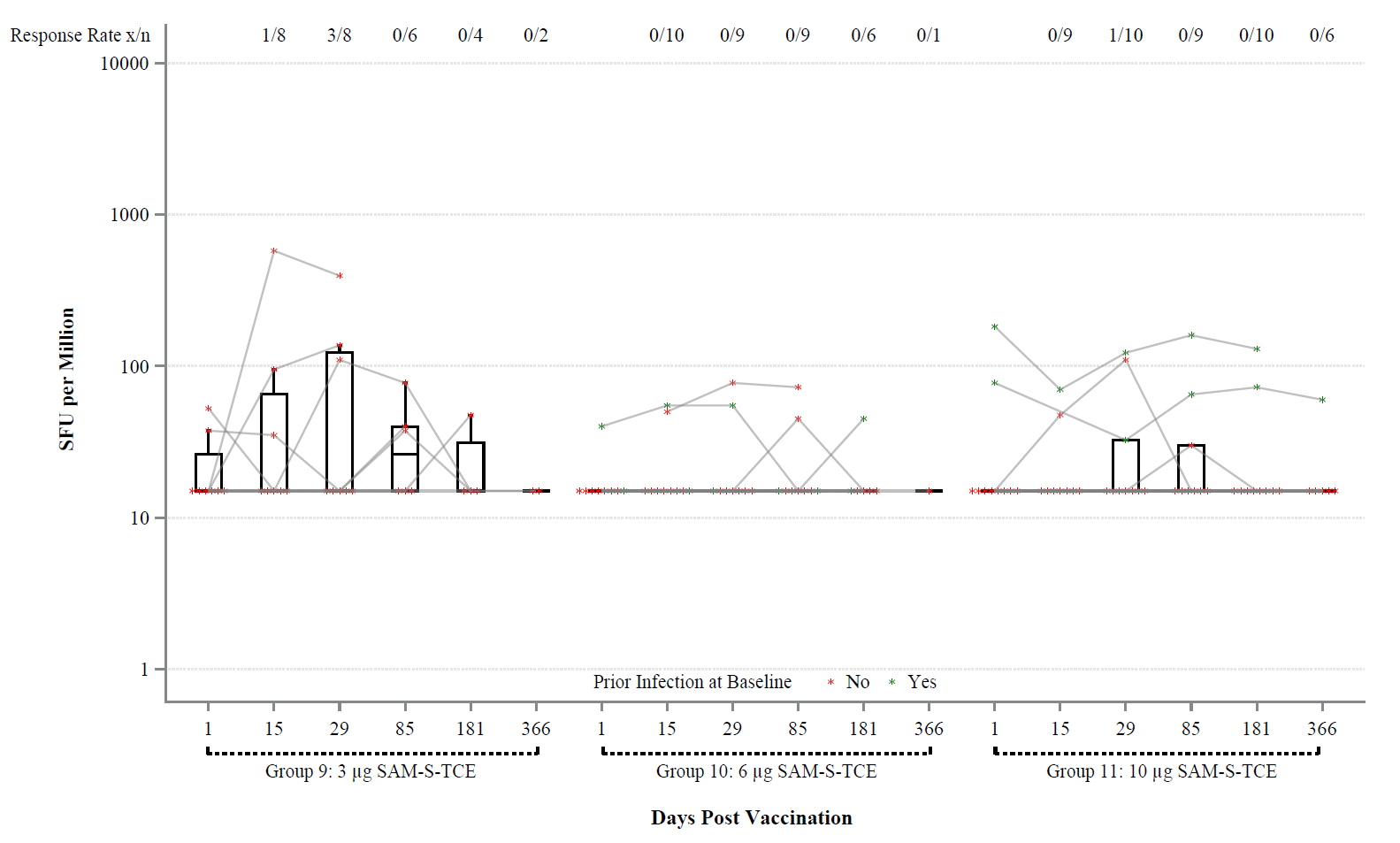


## **Supplementary Figure 13. Distribution of CD4 T Cells Expressing IFNγ and/or IL-2, stimulation with Spike peptides, 18-60 years of age, per-protocol population as measured by intracellular cytokine staining**


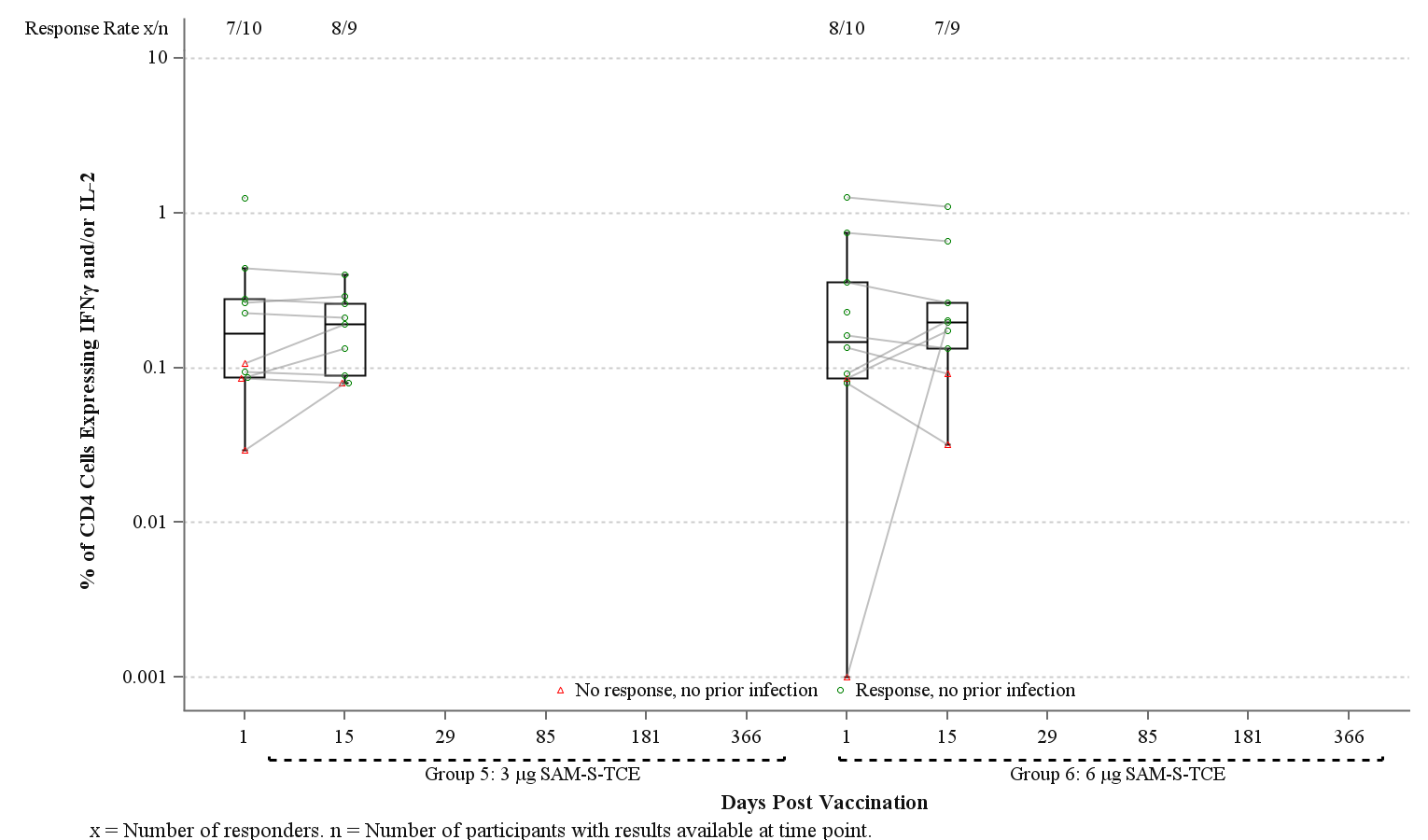


## **Supplementary Figure 14. Distribution of CD4 T Cells Expressing IFNγ and/or IL-2, stimulation with Spike peptides, >60 years of age, per-protocol population as measured by intracellular cytokine staining**


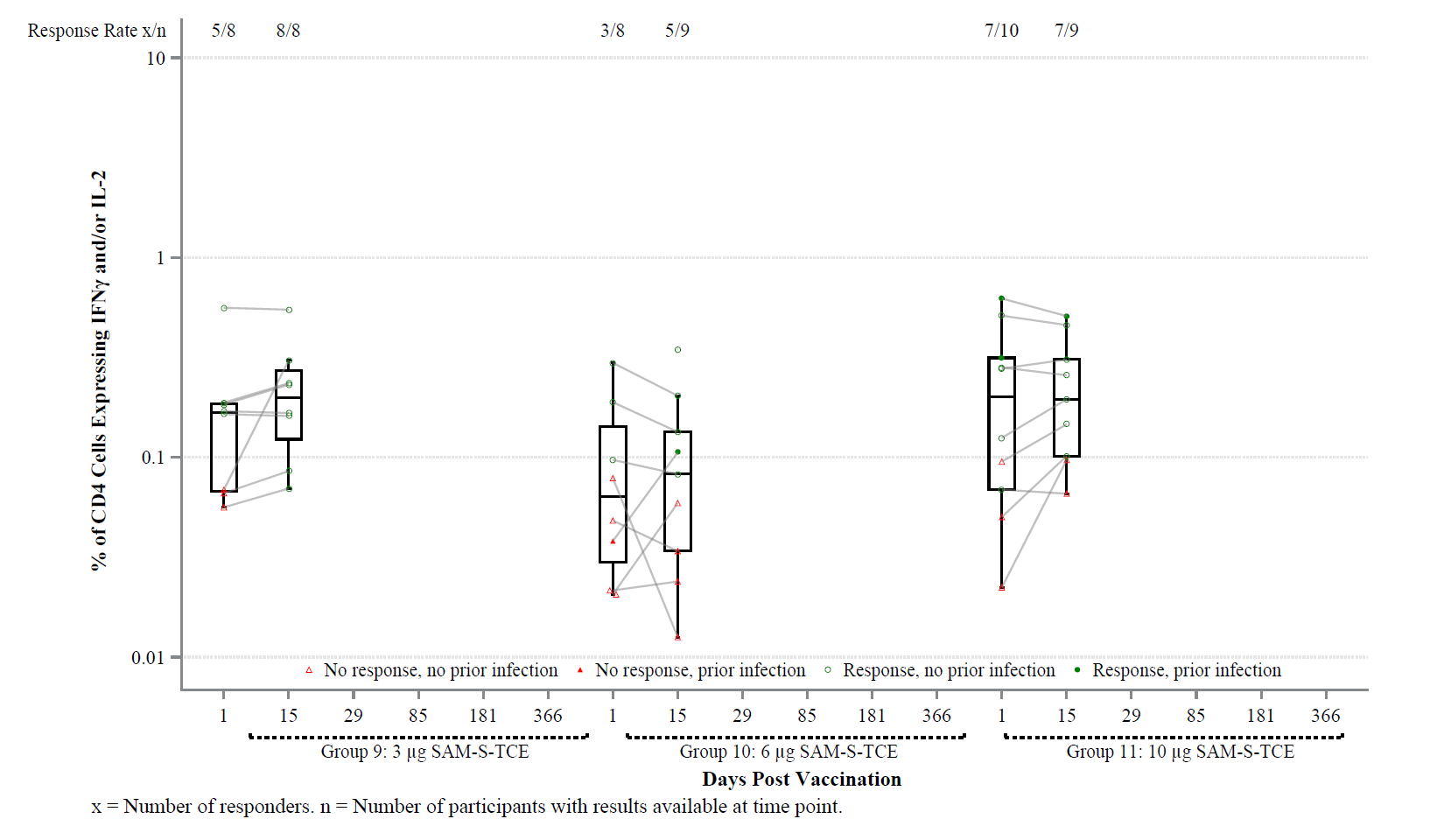


## **Supplemental Figure 15. Distribution of CD4 T cells expressing IFNγ and/or IL-2, stimulation with any TCE, 18-60 years of age, per-protocol population as measured by intracellular cytokine staining**


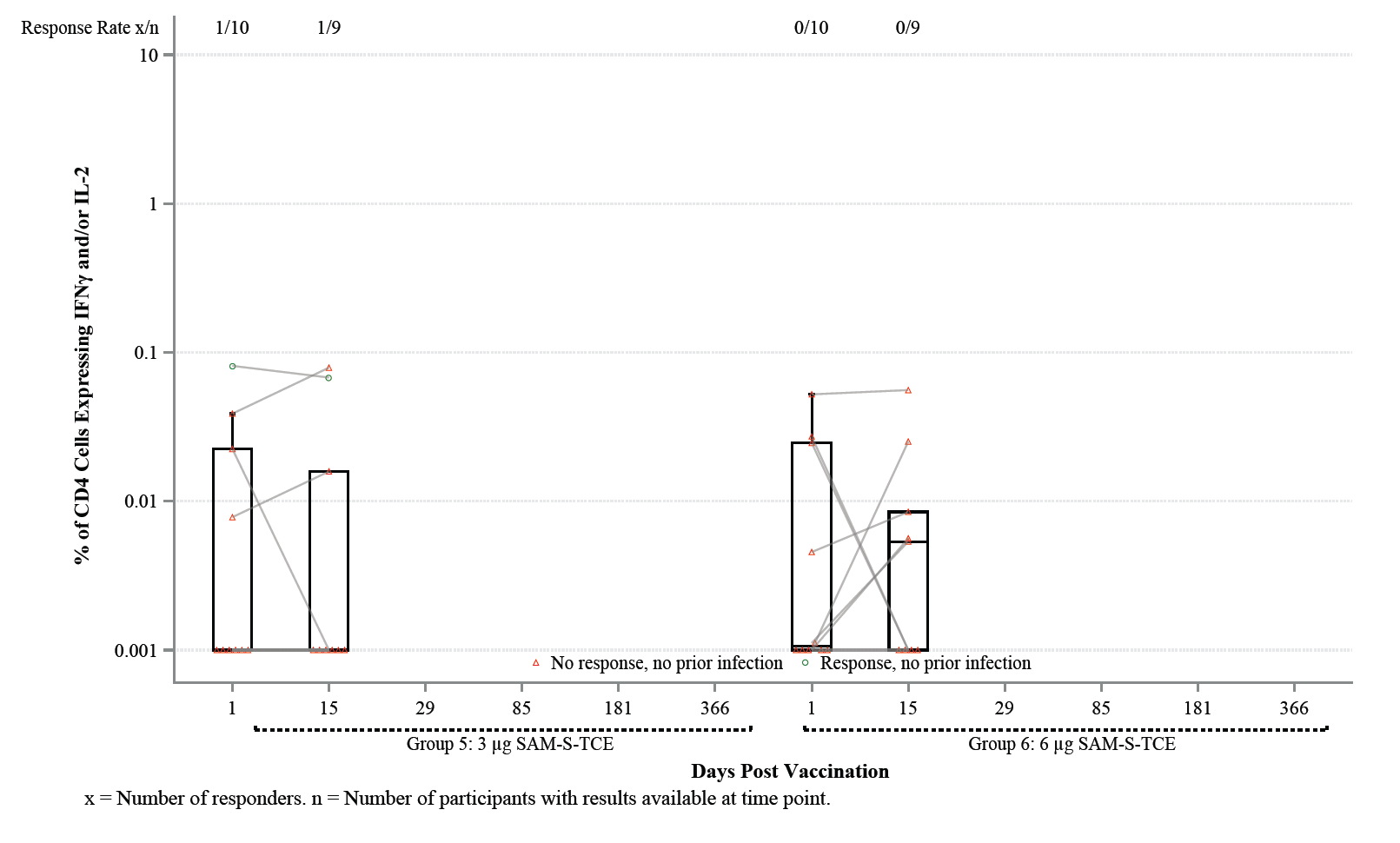


## **Supplemental Figure 16. Distribution of CD4 T cells expressing IFNγ and/or IL-2, stimulation with any TCE, >60 years of age, per-protocol population as measured by intracellular cytokine staining**


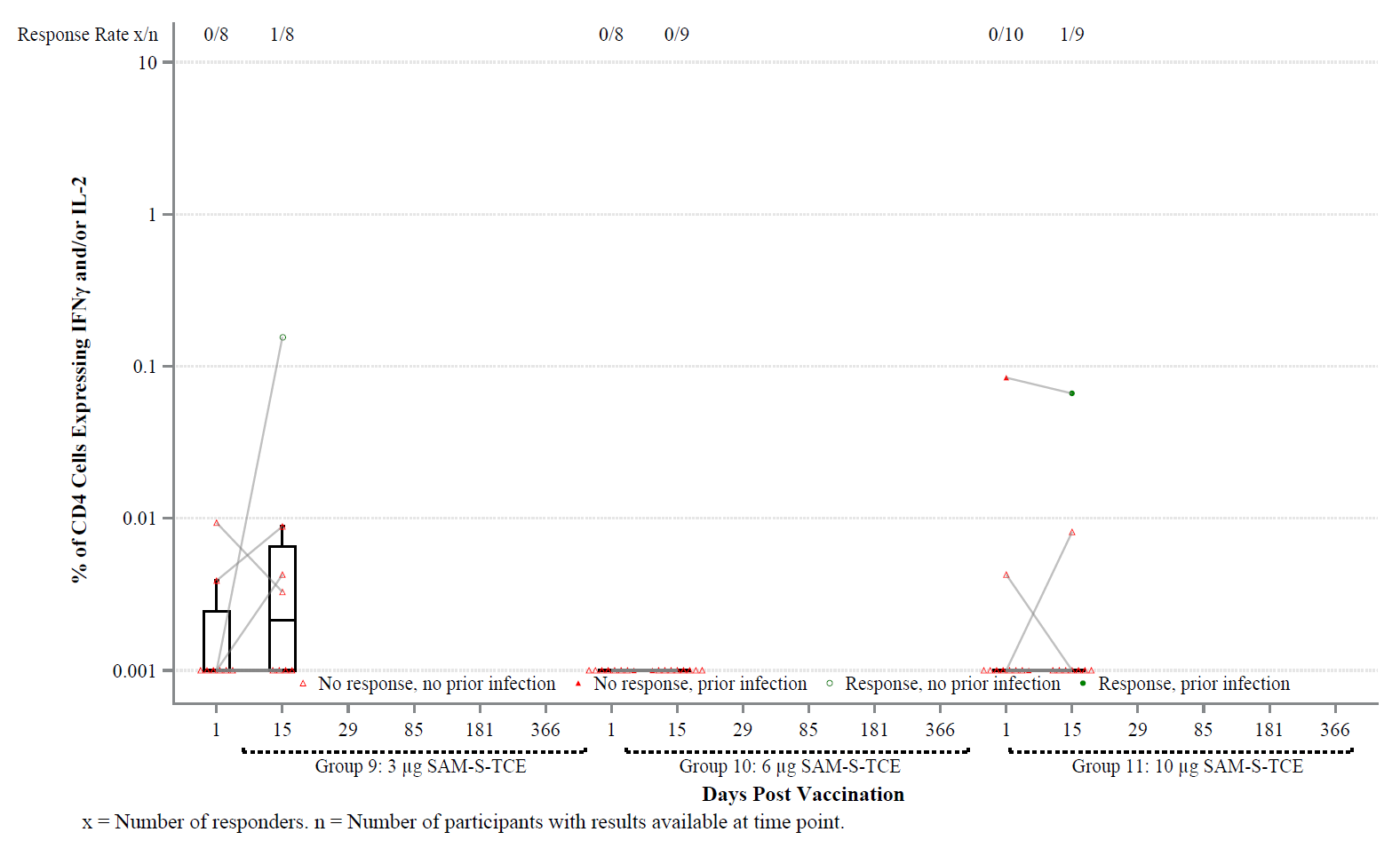


## **Supplemental Figure 17. Distribution of CD8 T cells expressing IFNγ and/or IL-2, stimulation with any TCE, 18-60 years of age, per-protocol population as measured by intracellular cytokine staining**


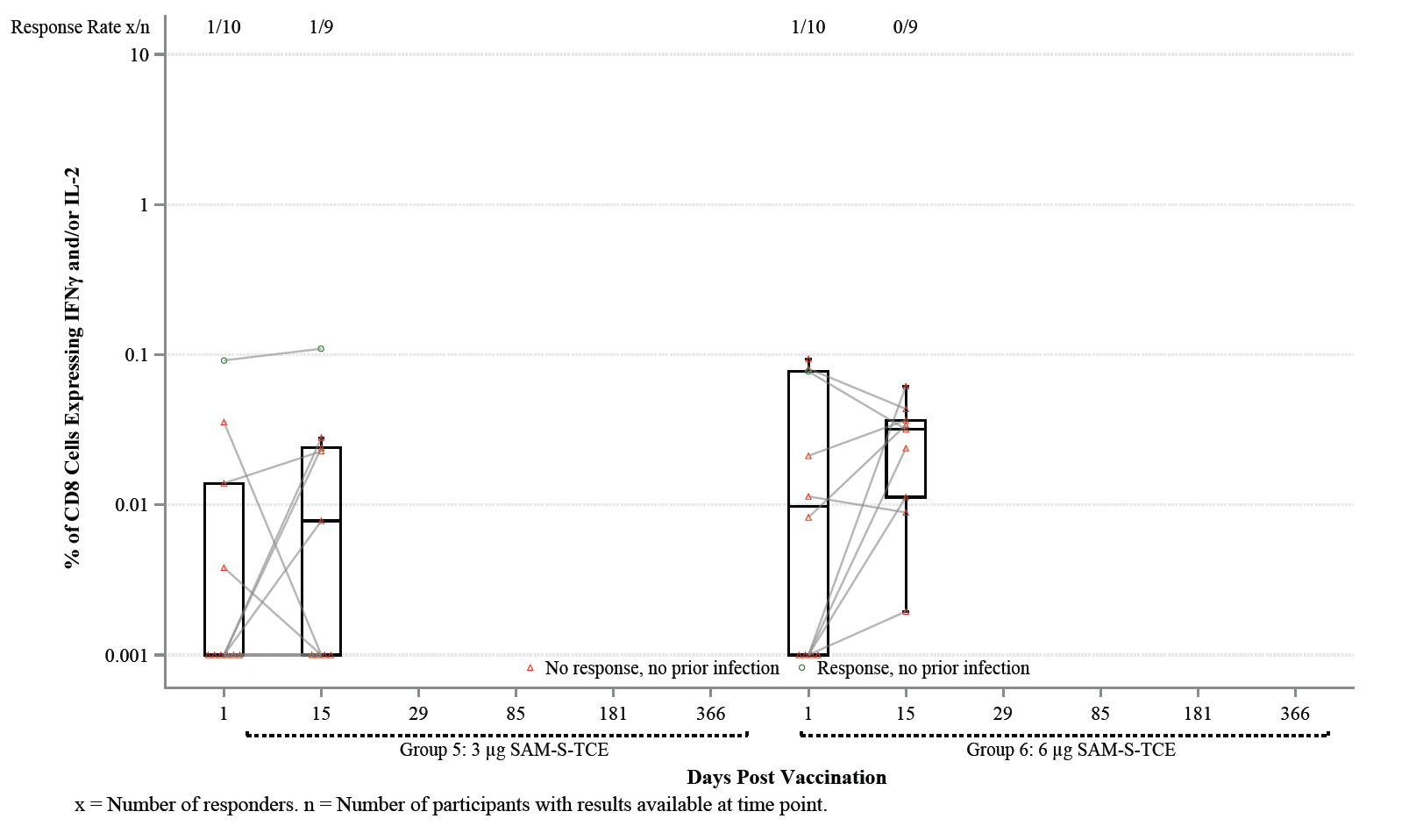


## **Supplemental Figure 18. Distribution of CD8 T cells expressing IFNγ and/or IL-2, stimulation with any TCE, >60 years of age, per-protocol population as measured by intracellular cytokine staining**


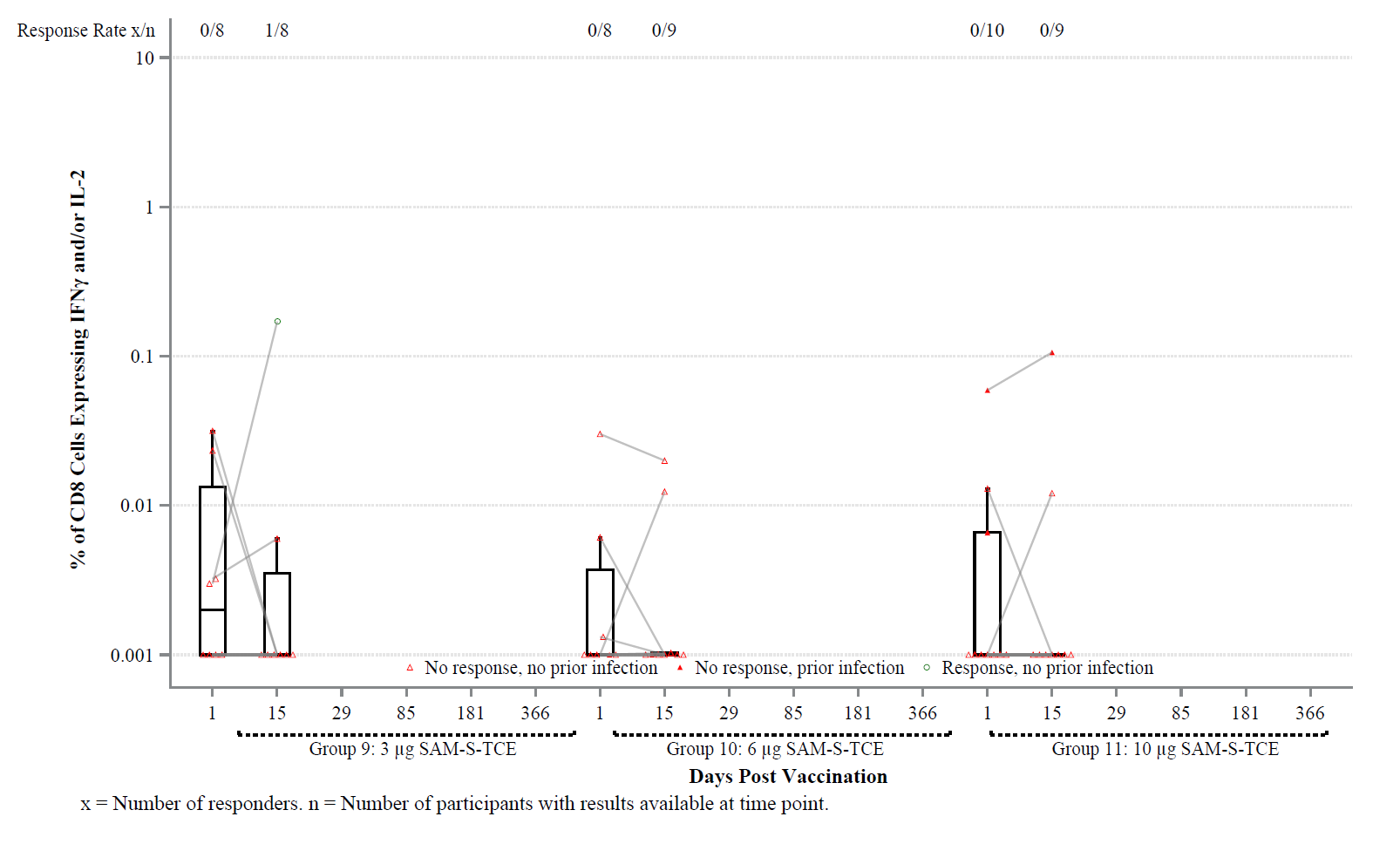


## **Supplementary Figure 19. Distribution of CD4 T cells expressing IL-4 or IL-5 or IL-13 and CD154, stimulation with Spike peptides, 18-60 years of age, per-protocol population as measured by intracellular cytokine staining**

**
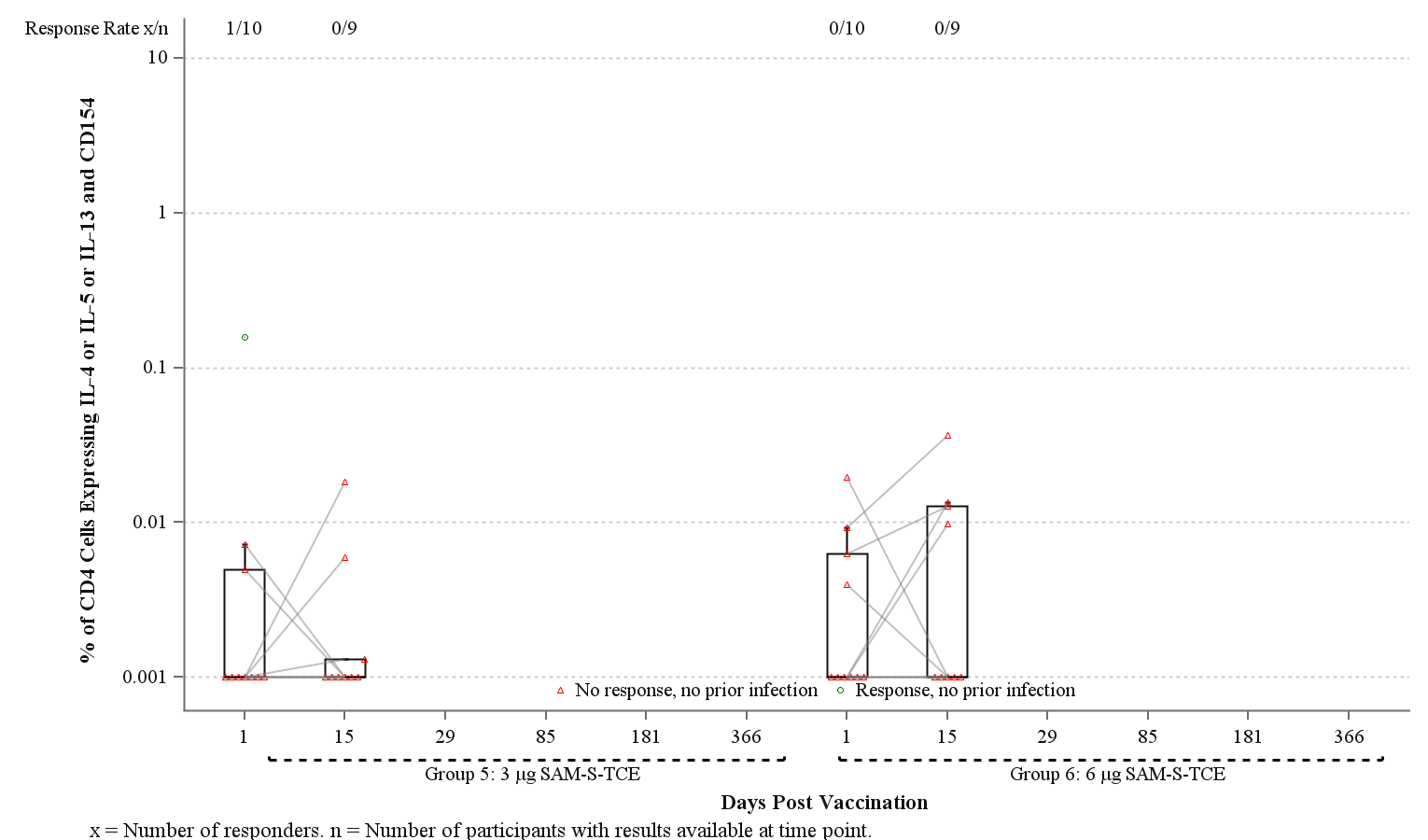
**

## **Supplementary Figure 20. Distribution of CD4 T cells expressing IL-4 or IL-5 or IL-13 and CD154, stimulation with Spike peptides, >60 years of age, per-protocol population as measured by intracellular cytokine staining**


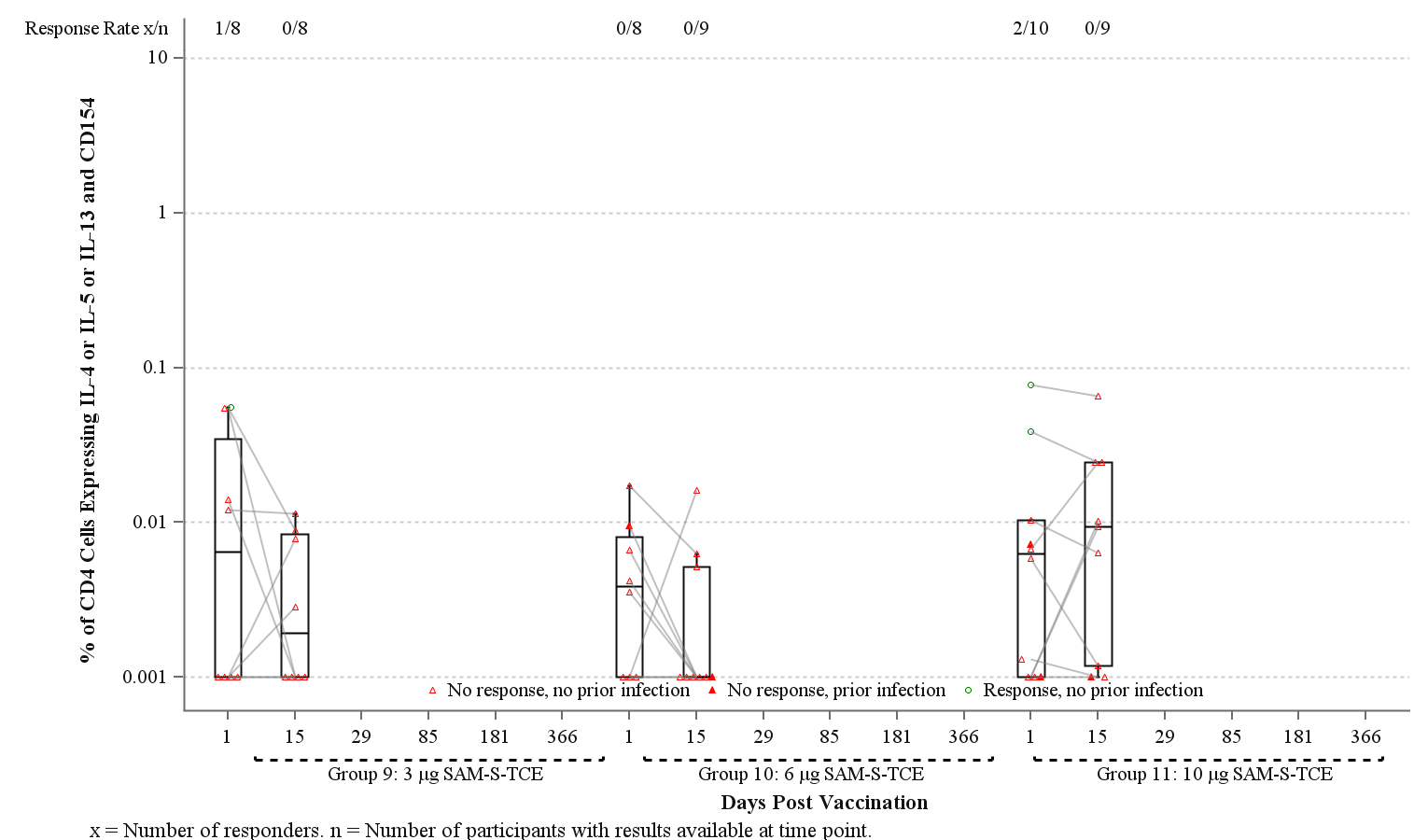


# **Supplementary References**

[1] Larsen SE, Berube BJ, Pecor T, Cross E, Brown BP, Williams BD, et al. Qualification of ELISA and neutralization methodologies to measure SARS-CoV-2 humoral immunity using human clinical samples. J Immunol Methods. 2021;499:113160. 10.1016/j.jim.2021.113160

[2] Shen X, Tang H, McDanal C, Wagh K, Fischer W, Theiler J, et al. SARS-CoV-2 variant B.1.1.7 is susceptible to neutralizing antibodies elicited by ancestral spike vaccines. Cell Host Microbe. 2021;29:529-39 e3. 10.1016/j.chom.2021.03.002

[3] Edara VV, Norwood C, Floyd K, Lai L, Davis-Gardner ME, Hudson WH, et al. Infection- and vaccine-induced antibody binding and neutralization of the B.1.351 SARS-CoV-2 variant. Cell Host Microbe. 2021;29:516-21 e3. 10.1016/j.chom.2021.03.009

[4] Edara VV, Hudson WH, Xie X, Ahmed R, Suthar MS. Neutralizing Antibodies Against SARS-CoV-2 Variants After Infection and Vaccination. JAMA. 2021. 10.1001/jama.2021.4388

[5] Xie X, Muruato A, Lokugamage KG, Narayanan K, Zhang X, Zou J, et al. An Infectious cDNA Clone of SARS-CoV-2. Cell Host Microbe. 2020;27:841-8 e3. 10.1016/j.chom.2020.04.004

[6] Edara VV, Pinsky BA, Suthar MS, Lai L, Davis-Gardner ME, Floyd K, et al. Infection and Vaccine-Induced Neutralizing-Antibody Responses to the SARS-CoV-2 B.1.617 Variants. N Engl J Med. 2021;385:664-6. 10.1056/NEJMc2107799

[7] Vanderheiden A, Edara VV, Floyd K, Kauffman RC, Mantus G, Anderson E, et al. Development of a Rapid Focus Reduction Neutralization Test Assay for Measuring SARS-CoV-2 Neutralizing Antibodies. Curr Protoc Immunol. 2020;131:e116. 10.1002/cpim.116

[8] Katzelnick LC, Coello Escoto A, McElvany BD, Chavez C, Salje H, Luo W, et al. Viridot: An automated virus plaque (immunofocus) counter for the measurement of serological neutralizing responses with application to dengue virus. PLoS Negl Trop Dis. 2018;12:e0006862. 10.1371/journal.pntd.0006862

[9] Dintwe O, Rohith S, Schwedhelm KV, McElrath MJ, Andersen-Nissen E, De Rosa SC. OMIP-056: Evaluation of Human Conventional T Cells, Donor-Unrestricted T Cells, and NK Cells Including Memory Phenotype by Intracellular Cytokine Staining. Cytometry A. 2019;95:722-5. 10.1002/cyto.a.23753

[10] Horton H, Thomas EP, Stucky JA, Frank I, Moodie Z, Huang Y, et al. Optimization and validation of an 8-color intracellular cytokine staining (ICS) assay to quantify antigen-specific T cells induced by vaccination. J Immunol Methods. 2007;323:39-54. 10.1016/j.jim.2007.03.002
